# A hydrogenotrophic *Sulfurimonas* is globally abundant in deep-sea oxygen-saturated hydrothermal plumes

**DOI:** 10.1038/s41564-023-01342-w

**Published:** 2023-03-09

**Authors:** Massimiliano Molari, Christiane Hassenrueck, Rafael Laso-Pérez, Gunter Wegener, Pierre Offre, Stefano Scilipoti, Antje Boetius

**Affiliations:** 1 Max Planck Institute for Marine Microbiology, Bremen, Germany; 2 Alfred Wegener Institute for Polar and Marine Research, Bremerhaven, Germany; 3 Leibniz Institute for Baltic Sea Research Warnemünde (IOW), Rostock, Germany; 4 MARUM Center for Marine Environmental Sciences, University of Bremen, Bremen, Germany; 5 Department of Marine Microbiology and Biogeochemistry, NIOZ, Royal Netherlands Institute for Sea Research, Den Burg, the Netherlands; 6 Center for Electromicrobiology, Department of Biology, Aarhus University, Aarhus, Denmark; 7 Present Address: Systems Biology Department, Centro Nacional de Biotecnología (CNB-CSIC), Madrid, Spain

**Keywords:** Microbial ecology, Water microbiology, Biogeochemistry

## Abstract

Members of the bacterial genus *Sulfurimonas* (phylum Campylobacterota) dominate microbial communities in marine redoxclines and are important for sulfur and nitrogen cycling. Here we used metagenomics and metabolic analyses to characterize a *Sulfurimonas* from the Gakkel Ridge in the Central Arctic Ocean and Southwest Indian Ridge, showing that this species is ubiquitous in non-buoyant hydrothermal plumes at Mid Ocean Ridges across the global ocean. One *Sulfurimonas* species, ^U^*Sulfurimonas pluma*, was found to be globally abundant and active in cold (<0−4 °C), oxygen-saturated and hydrogen-rich hydrothermal plumes. Compared with other *Sulfurimonas* species, ^U^*S. pluma* has a reduced genome (>17%) and genomic signatures of an aerobic chemolithotrophic metabolism using hydrogen as an energy source, including acquisition of A2-type oxidase and loss of nitrate and nitrite reductases. The dominance and unique niche of ^U^*S. pluma* in hydrothermal plumes suggest an unappreciated biogeochemical role for *Sulfurimonas* in the deep ocean.

## Abstract (web summary)

An uncultivated, aerobic chemolithotrophic *Sulfurimonas* species with a reduced genome is abundant across diverse, hydrogen-rich hydrothermal plumes in the deep ocean.

## Main

The genus *Sulfurimonas* belongs to the phylum Campylobacterota (former class Epsilonproteobacteria). It was originally proposed after the isolation of *Sulfurimonas autotrophica* from sediments collected at a deep-sea hydrothermal vent^1^. Since then, 12 distinct *Sulfurimonas* species have been isolated from oxygen-deficient environments^2–11^. Bassed on 16S rRNA gene sequences, this mesophilic and chemolithoautotrophic bacterial genus is ubiquitous and a dominant member of microbial communities inhabiting redoxcline environments^12^, including sulfidic environments at deep-sea hydrothermal vents^13–17^. The described members of the genus *Sulfurimonas* occupy habitats defined by moderate temperatures, elevated hydrogen sulfide concentrations and low oxygen concentrations (<40 µM) compared with the habitats of other hydrothermal Campylobacterota members (that is, *Sulfuruvum*^16^) and marine sulfur oxidizers (that is, SUP05^18,19^). Yet, abundant *Sulfurimonas* 16S rRNA gene sequences have also been reported in non-buoyant stage of hydrothermal plumes^14,20–24^. Hydrothermal plumes occur wherever hot anoxic hydrothermal fluids emitted from the seabed mix with cold oxygenated seawater. They can rise to hundreds of metres off the seafloor and disperse thousands of kilometres away from their source^25^. At the non-buoyant stage, hydrothermal plumes consist mostly of cold and oxygen-saturated seawater with highly dilute admixtures of hydrothermal fluid (<0.01%)^25,26^. For this reason, non-buoyant hydrothermal plumes have not been considered a permanent niche and habitat for *Sulfurimonas*. The repeated detection of *Sulfurimonas* sequences in such plumes was explained by passive transport from seafloor and subseafloor environments^26^. However, no study has directly tested whether non-buoyant plumes provide a suitable environment for growth of specific members of *Sulfurimonas*. The hydrothermal plumes contain substantial amounts of inorganic reduced gases (H_2_S, CH_4_ and H_2_) and metals (Fe, Mn, Cu, Zn and Co)^27^, which have considerable impact on ocean chemistry^28^. Thus, the identification and elucidation of the physiology of microorganisms growing in the plume are crucial to understanding the ocean’s biogeochemistry.

In this study, we investigated the distribution and function of *Sulfurimonas* in the hydrothermal plumes. We studied its ribotypes, genotypes and metabolism in two vent plumes of Gakkel Ridge and in one plume of the Southwest Indian Ridge (SWIR), and compared these with publicly available data from other vent plumes of Mid Ocean Ridges and other environments hosting *Sulfurimonas* sp. Our hypothesis is that non-buoyant hydrothermal plumes are a suitable environment for specific members of *Sulfurimonas*.

## Results

### An uncharacterized *Sulfurimonas* in hydrothermal plumes

**Extended Data Fig. 1:**
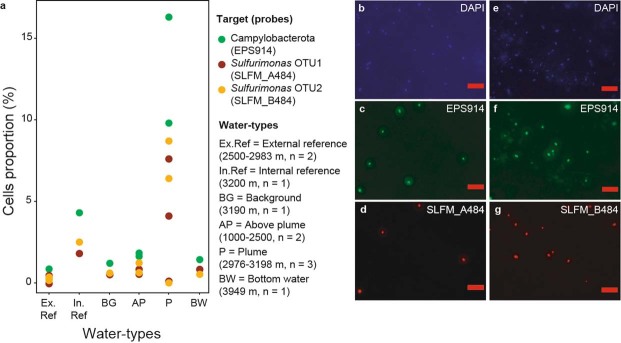
Abundance of Campylobacterota and hydrothermal plume *Sulfurimonas* sp. cells at Arctic sites. **a**, Proportion of Campylobacterota and hydrothermal plume *Sulfurimonas* sp. to DAPI-stained cell counts in different water types at Aurora vent and reference sites (water depth and number of samples are reported in parenthesis); **b** − **g**, fluorescence micrographs of microbes in the Aurora plume (station PS86-55) stained with DAPI (blue; **b** and **e**) and simultaneously (double CARD-FISH) with specific probes for Campylobacterota (green; **c** and **f**) and for Arctic *Sulfurimonas* OTU1 (red; **d**) and OTU2 (red; **g**). Panels b–g represents observations made from independent samples (n = 3). Scale bars 10 μm (b–g). Source data

**Extended Data Fig. 2:**
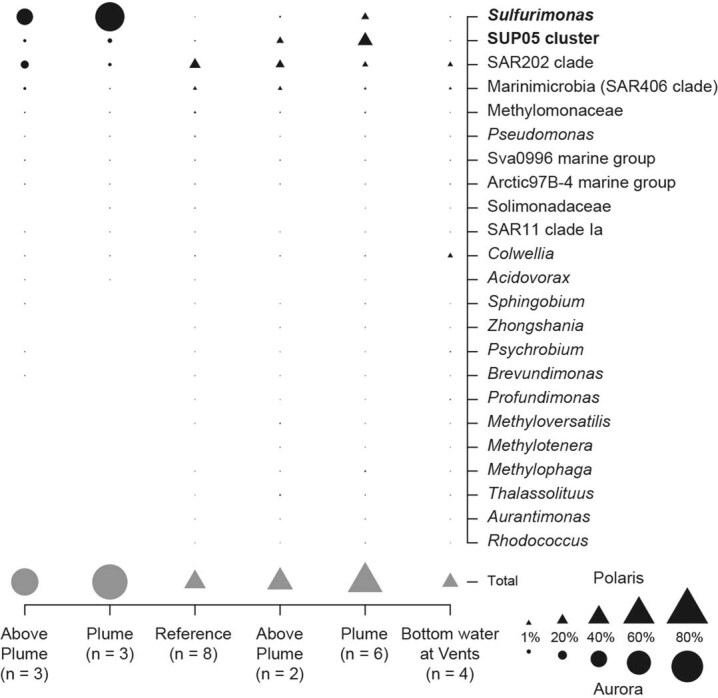
Active bacterial community structure at Aurora and Polaris water types. Dominant active bacterial taxa (cut-off ≥1.5 %) as detected by 16 S rRNA sequencing (cDNA). Average of sequences proportion is reported. The most dominant and active chemolithotrophs are in bold. Number of observations (n) is reported in parentheses. Source data

We investigated microbial community compositions in deep-sea water samples from the Gakkel Ridge and the Southwest Indian Ridge (SWIR). Campylobacterota accounted for 70−79% and 9−19% of all 16S rRNA sequences of non-buoyant hydrothermal plumes of Aurora (3,360−3,575 m depth) and Polaris (2,574−2,846 m depth) mounds^29^, respectively, and up to 16% of total microbial cells (Extended Data Fig. 1a). Almost all Campylobacterota-affiliated 16S rRNA sequences (>99%) in the non-buoyant hydrothermal plumes of Gakkel Ridge and in seawater from a ridge valley of the SWIR belonged to the genus *Sulfurimonas* (Supplementary Table 1 and Extended Data Fig. 2). In addition, more than 97% of the *Sulfurimonas* sequences of these three remote sites on ultraslow spreading ridges belonged to two closely related operational taxonomic units (OTU1 and OTU2), with a similarity of 99.5%. Fluorescence in situ hybridization using both a Campylobacterota-specific rRNA probe and tailored highly specific probes for the two detected *Sulfurimonas* OTUs confirmed these results (Extended Data Fig. 1b–f).

On the basis of environmental DNA retrieved from hydrothermal plume samples from Gakkel Ridge, we obtained two near-complete (93.6 − 99.95% completeness) and high-quality draft^30^ *Sulfurimonas* metagenome-assembled genomes (MAG-1 and MAG-2; Supplementary Table 2). These two MAGs have an average nucleotide identity (ANI) of 98.9%, confirming that the Gakkel Ridge plumes host two closely related strains of the same *Sulfurimonas* species. The 16S rRNA gene sequences from the MAGs are 99.5−100% identical with the dominant OTUs of the 16S rRNA gene amplicons, pointing toward the prevalence of the same *Sulfurimonas* strains in the Gakkel Ridge and the SWIR water samples, which are more than 15,000 km apart and in different deep-water current systems.

**Fig. 1:**
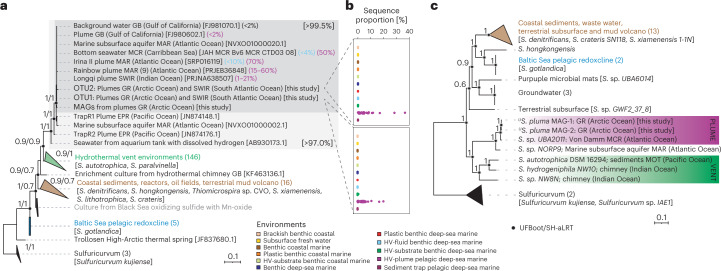
Phylogeny and environmental distribution of hydrothermal plume *Sulfurimonas* sp. **a**, Rooted phylogenetic tree of 16S rRNA gene sequences of *Sulfurimonas* species (*S*.) and closest relatives, including isolates and environmental sequences, with *Sulfuricurvum* as outgroup. The integer numbers and the percentage in parentheses indicate the number of sequences in a given branch and the contribution of *Sulfurimonas* sequences to the total number of sequences in Illumina amplicon sequencing datasets, respectively. In square brackets, the percentage of 16S rRNA gene identity is reported for the plume *Sulfurimonas* cluster. **b**, The two plots show the contribution of the hydrothermal plume *Sulfurimonas* ecotypes (see Extended Data Fig. 3 for details) to the total number of sequences. **c**, Rooted outgroup phylogenetic tree based on concatenated SCG = 258 of *Sulfurimonas* and *Sulfuricurvum* using partition substitution models. Hydrothermal vent (HV) environments include: chimney, sediments, fluids and animal body/nest. The scale bar represents the expected number of changes per nucleotide position. UFBoot and SH-aLRT values are based on 1,000 replicates. Best substitution model for 16S rRNA gene tree: TVMe+I + G4. GB: Guaymas Basin; MAR: Mid Atlantic Ridge; MCR: Mid Cayman Rise; GR: Gakkel Ridge; SWIR: Southwest Indian Ridge; EPR: East Pacific Rise.

The *Sulfurimonas* species that is most closely related to the hydrothermal plume ribotype and genotype is *Sulfurimonas autotrophica*, with 94% 16S rRNA gene identity and an ANI of only 74.2%. *S. autotrophica* has been isolated from Pacific Ocean hydrothermal sediments^1^. Yet, phylogenetic analyses based on 16S rRNA genes assigned the plume-hosted *Sulfurimonas* sequences to an independent clade which included neither *S. autotrophica* nor *Sulfurimonas* sequences from other hydrothermal vent environments (Fig. 1a). Instead, our *Sulfurimonas* phylotypes form a well-supported clade together with sequences derived from hydrothermal plumes in the Atlantic Ocean (Mid Atlantic Ridge, Mid Cayman Ridge), in the Gulf of California (Guaymas Basin), in the Pacific Ocean (East Pacific Rise) and from an oxic subsurface aquifer (Mid Atlantic Ridge). This phylogenetic placement is supported by phylogenomic trees based on concatenated single-copy genes (SCGs) (Fig. 1c). The comparative analysis of *Sulfurimonas* genomes identified 7,569 clusters of orthologous genes. About 13% of these represent the core genome of this genus (Extended Data Fig. 4a). Similar results were recently reported in a more comprehensive pangenomic survey of the genus *Sulfurimonas*^8^. Altogether, these findings support the occurrence of a previously uncharacterized *Sulfurimonas* taxon in non-buoyant hydrothermal plumes. According to the standards for microbial uncultivated species^31,32^, we propose to name this uncultivated (U) taxon ‘^U^*Sulfurimonas pluma*’.

### ^U^*S. pluma* inhabits oxygen-saturated hydrothermal plumes

**Extended Data Fig. 3:**
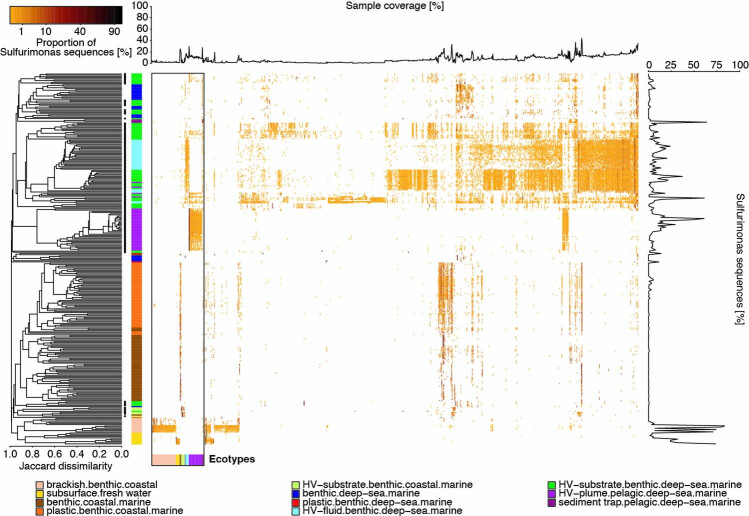
Heatmap of the distribution of *Sulfurimonas* oligotypes across samples. Samples are ordered based on average linkage clustering of Jaccard dissimilarities (left panel). Asterisks indicate samples of hydrothermal origin. Top panel: percentage of samples an oligotype occurs in (sample coverage). Right panel: percentage of *Sulfurimonas* sequences of total sequences per sample. Bottom panel: Ecotypes per environmental category (brackish pelagic and benthic, as well as pelagic deep-sea and hydrothermal plume were pooled). Source data

To identify the environmental factors shaping the habitat of the here described ^U^*S. pluma* taxon, we compared the hypervariable V3−V4 region of the 16S rRNA genes of *Sulfurimonas* obtained from the Arctic and from SWIR to those found in other environments (Extended Data Fig. 5), and assigned environmental characteristics (Supplementary Table 3). The studied 308 samples contained 1,389 *Sulfurimonas* oligotypes (amplicon sequence variants distinguished on the basis of information entropy determined for each nucleotide position^33^; Supplementary Fig. 1 and Table 4). On the basis of the presence and proportion of oligotypes within and among the environmental categories, they formed 149 ecotypes, 38 of which were specific to hydrothermal plumes (Extended Data Fig. 3). Two of these oligotypes represented between 28% and 97% of the *Sulfurimonas* sequences in the hydrothermal plume samples. These oligotypes were identical to the dominant OTUs in Gakkel and SWIR plumes (OTU1 and OTU2). These results confirm the existence of non-buoyant plume-specific *Sulfurimonas* ecotypes that are very rare in other hydrothermal environments (sequence proportions <1%; Fig. 1b). The ^U^*S. pluma* cluster of closely related sequences (>99.5% identity) dominated hydrothermal plumes across the ridge systems of the Central Arctic, Atlantic and Indian/Southern Oceans (Fig. 1a). The same ribotype was also found in the plume and the surrounding water column of the Guaymas Basin in the Gulf of California^34^, but with low proportions to the total bacterial community (Fig. 1a).

**Extended Data Fig. 4:**
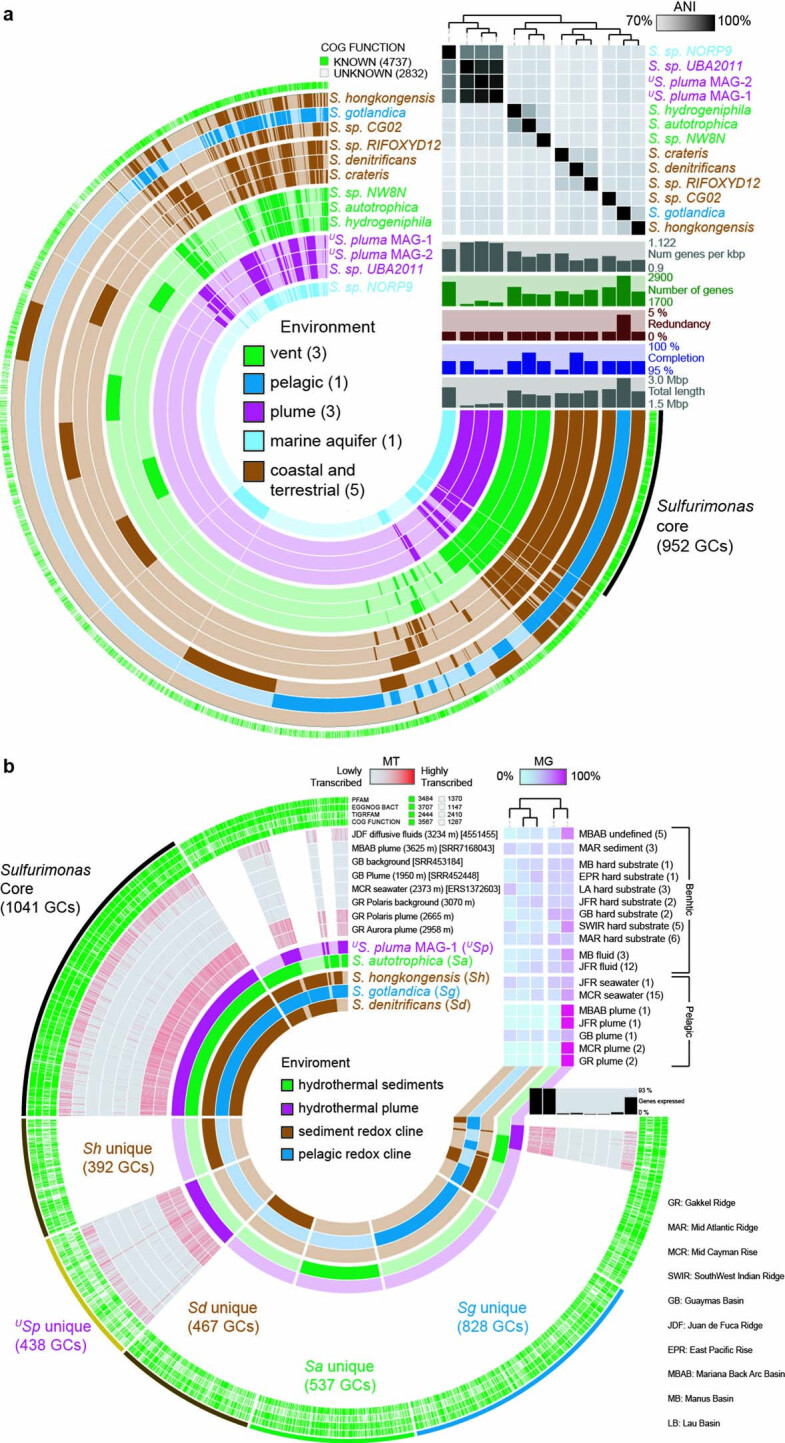
Pangenomic analyses of hydrothermal plume ^U^*Sulfurimonas pluma*. Pangenome analysis of *Sulfurimonas* isolate genomes and MAGs from different environments. Starting from the centre, the first 13 circles show the occurrence of gene clusters in a given *Sulfurimonas* genome. The external circle reports the gene clusters in which at least one gene was functionally annotated to COGs. The 7569 gene clusters (GCs) are organized based on their distribution across the genomes. The genomes are organized based on gene clusters they share (using Euclidian distance and Ward clustering). The barplots show some general features of each genome, the heatmap shows the ANI between genomes. **b**, Metapangenome analysis of *Sulfurimonas* isolate genomes (*S. denitrificans*, *S. hongkongebsis*, *S. gotlantica*, *S. autotrophica*) and hydrothermal plume ^*U*^*S. pluma*. Starting from the centre: the first 5 circles show the occurrence of gene clusters in a given *Sulfurimonas* genomes; then the following 8 circles report the expression of gene clusters of ^*U*^*S. pluma* in hydrothermal plume, seawater and fluid as inferred from metatransciptomic (MT) read recruitment (only representatives for available ridge systems are reported); finally, the external circles report the gene clusters in which at least one gene was functionally annotated (green and grey for known and unknown functions, respectively). The 4854 gene clusters (GCs) and the genomes are organized based on their distribution across the genomes and on gene clusters they share (using Euclidian distance and Ward clustering), respectively. The barplot shows the percentage of genes expressed, the heatmap shows the percentage of *Sulfurimonas* reads recruited by five genomes (inner circle) from metagenomes (MG; average value is reported for habitats having more than one metagenome available per ridge system; the number of metagenomes is reported in parenthesis). In both panels the dendrograms represents the hierarchical clustering of genomes based on the occurrence of gene clusters.

**Extended Data Fig. 5:**
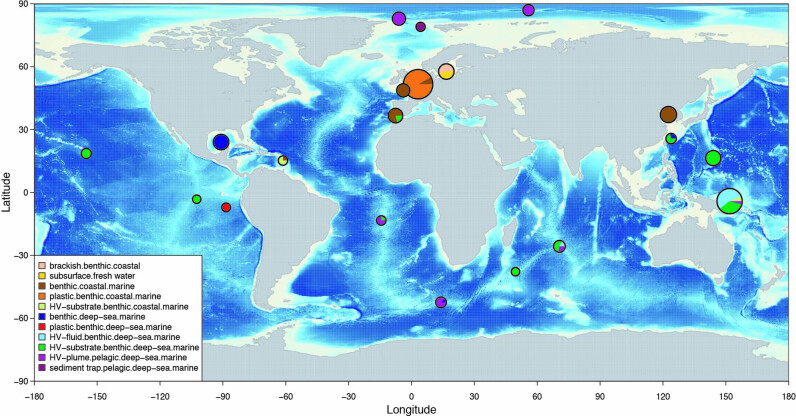
Samples’ location for oligotyping. World map showing the geographic origin, and environment of the samples used for the analysis of *Sulfurimonas* oligotypes. The point size is proportional to the number of samples collected at each location. HV: hydrothermal vent.

The specificity of ^U^*S. pluma* to hydrothermal plumes is also supported by recruitment analysis of metagenomic and metatranscriptomic reads from different hydrothermal environments. MAG-1 of ^U^*S. pluma* recruited preferentially metagenomic reads from hydrothermal plumes rather than from benthic hydrothermal environments (Extended Data Fig. 4b). ^U^*S. pluma* MAG-1 recruited transcripts from all seawater metatranscriptomes, yet the transcription of more than 60% of all its gene clusters was limited to samples from Gakkel Ridge non-buoyant plumes and from a diffusive fluid sample from Juan de Fuca Ridge (Extended Data Fig. 4b). The recruitment of few metagenomic and metatranscriptomic reads from Guaymas Basin hydrothermal plumes is consistent with previous studies reporting low proportions of *Sulfurimonas* sequences in the plume^34,35^ (Fig. 1a). As to the plume samples from Beebe vent at Mid Cayman Rise, the recorded water depth (4,900 m) suggests that water samples were collected in the deepest part of the rising plume, where the microbial community is still mostly influenced by benthic communities, yet comprises few microbes growing within the hydrothermal non-buoyant plume^36,37^.

### ^U^*S. pluma* lost typical *Sulfurimonas* genes

The ^U^*S. pluma* MAGs have a size of 1.68−1.77 Mbp, which is considerably smaller than the genomes of other *Sulfurimonas* strains, including *S. gotlandica* GD1 isolated from a pelagic redox environment (Extended Data Fig. 4a). Despite their small size, the MAGs of ^U^*S. pluma* are as complete as the closed genome of *S. autotrophica*, based both on reference SCGs and machine learning algorithm methods (Supplementary Table 2). The smaller size is partly explained by the high gene density (that is, number of genes per kbp) of ^U^*S. pluma* MAGs, which exceeds those of all other *Sulfurimonas* genomes (Extended Data Fig. 4a). This suggests partial deletion of non-coding DNA sequences, resulting in a streamlined genome. Nonetheless, the ^U^*S. pluma* MAGs lack several genes that code for core and apparent niche functions of isolated *Sulfurimonas* strains, such as for sulfide oxidation and nitrate/nitrite reduction^8,12^ (Table 1 and Supplementary Table 5). We investigated whether misassembly or binning errors caused the absence of these genes. None of the 16 individual genomic bins that were the basis for the consensus ^U^*S. pluma* MAGs contained the missing functional genes. Furthermore, contigs obtained from the individual assemblies (*n* = 12) and the co-assembly (*n* = 1) of Gakkel Ridge seawater metagenomes did not contain additional *Sulfurimonas/*Campylobacterota genes that did not bin to the ^U^*S. pluma* MAGs. The alignment of ^U^*S. pluma* MAGs with the *S. autotrophica* genome showed that only single genes or operons were missing in conserved genomic regions located in the largest contigs (>40 kpb). These results excluded that the observed genome reduction was an artefact of assembly and binning procedures.

**Table 1:** Genomic comparison of ^U^*S. pluma* with isolated *Sulfurimonas* strains

	Sd	Sh	Sg	Sa	Sp1	(Putative) characteristics
Genome size (Mbp)	2.20	2.30	2.95	2.15	1.77	The Sp1 genome is 17% to 40% reduced compared with other *Sulfurimonas* strains.
Hydrogen oxidation						
(NiFe)-hydrogenase	+	+	+	+	+	Sp1 encodes only the oxygen-sensitive type (group 1b).
Dissimilatory sulfur oxidation						
Flavocytochrome c sulfide dehydrogenase	+	+	+	−	+	In Sp1 putative primary enzyme of sulfide oxidation pathway.
Sulfide:quinone reductase	+	+	+	+	(+)	Canonical enzyme for sulfide oxidation in *Sulfurimonas*. Sp1 contains only type VI (missing types I–V).
Dissimilatory nitrogen reduction						
Nitrate reductase	+	+	+	+	−	Niche-specific enzymes for *Sulfurimonas*.
Nitrite reductase	+	+	+	+	−	
Nitric oxide reductase	+	+	+	+	−	
Carbon fixation						
Oxoglutarate:ferredoxin oxidoreductase	+	+	+	+	+	Sp1 encodes a five-subunit version of these enzymes compared with typical four-subunit enzyme, potentially resulting in a higher oxygen tolerance of the enzyme.
Pyruvate:ferredoxin oxidoreductase	+	+	+	+	+	
Oxygen reduction						
cbb3-type cytochrome c oxidase	+	+	+	+	+	Canonical enzyme for *Sulfurimonas*. High affinity for oxygen, thus optimized for microaerobic environments.
caa3-type cytochrome c oxidase	−	−	−	−	+	Canonical enzyme for aerobic bacteria.
Oxidative stress						
Superoxide dismutase	+	+	+	+	−	Primary antioxidant system in aerobic organisms.
Periplasmic superoxide reductase	−	−	−	−	+	Putative role in scavenging the periplasmic superoxide.

Sd: *S. denitrificans*; Sh: *S. hongkongensis*; Sg: *S. gotlandica*; Sa: *S. autotrophica*; Sp1: ^U^*S. pluma* MAG-1. For details, see Supplementary Table 5.

### ^U^*S. pluma* is a hydrogen oxidizer

**Fig. 2:**
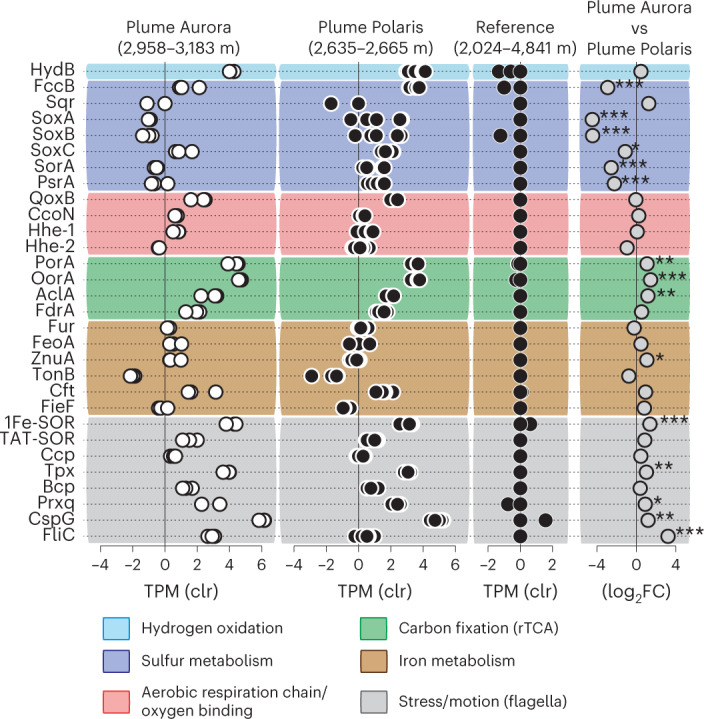
Transcriptome of ^U^*S. pluma*. The expression of marker genes for the main metabolic pathways of *Sulfurimonas* sp. from Gakkel Ridge plumes (Aurora and Polaris) and reference seawater. The gene expression is centred log-ratio transformed (clr). Differential expression of genes between the plumes of Aurora (*n* = 3) and Polaris (*n* = 6) is reported as log2-fold-change (log_2_FC). The pairwise statistical test is based on quantile-adjusted conditional maximum-likelihood (qCML) method and the likely value was adjusted by applying FDR. *P_adjust_ < 0.05; **P_adjust_ < 0.01; ***P_adjust_ < 0.001. hydB: (NiFe)-hydrogenase Group 1b, large subunit; fccB: flavocytochrome c sulfide dehydrogenase; sqr: sulfide:quinone reductase; soxA, soxB, soxC: sulfur oxidation proteins; sorA: sulfite dehydrogenase; psrA: polysulfide reductase, subunit I; qoxB: cytochrome c oxidase, caa3-type, subunit I; ccoN: cytochrome c oxidase, cbb3-type, subunit I; hhe-1 and hhe-2: bacteriohemerythrins; porA: pyruvate:ferredoxin oxireductase, subunit I; oorA: oxoglutarate:ferredoxin oxireductase, subunit I; aclA: ATP-dependent citrate lyase, subunit I; fdrA: fumarate reductase, subunit I; fur: iron uptake regulation; feoA: Fe^2+^ uptake; znuA: Mn^2+^/Zn^2+^ uptake; tonB: siderophore transport; cft: ferritin; 1Fe-SOR and TAT-SOR: superoxide reductases; ccp: cytochrome c peroxidase; tpx, bcp and prxq: peroxiredoxins; cspG: cold shock-like protein; fliC: flagellin; rTCA: reductive tricarboxylic acid cycle. Source data

The MAGs of ^U^*S pluma* encode a membrane-bound (NiFe)-hydrogenase, which is the highest expressed catabolic gene in all metatranscriptomes of both Gakkel Ridge plumes (Fig. 2). (NiFe)-hydrogenases are ubiquitous in *Sulfurimonas* genomes, yet an expression of the encoding genes by *Sulfurimonas* in hydrothermal plumes has not been reported before (Supplementary Note 1). The fact that the hydrogenase was >13 to >500 times higher expressed than genes for sulfur oxidation suggests that hydrogen is a critical energy source to sustain the growth of ^U^*S. pluma* in the Aurora plume (Fig. 2), where it was most abundant and active (Supplementary Table 1 and Extended Data Fig. 2). Laboratory experiments with cultures of *S. denitrificans* also found that this species grows more efficiently when supplied with hydrogen than with thiosulfate as electron donor^38^, suggesting that hydrogen can be an important energy substrate for the genus *Sulfurimonas*.

**Fig. 3:**
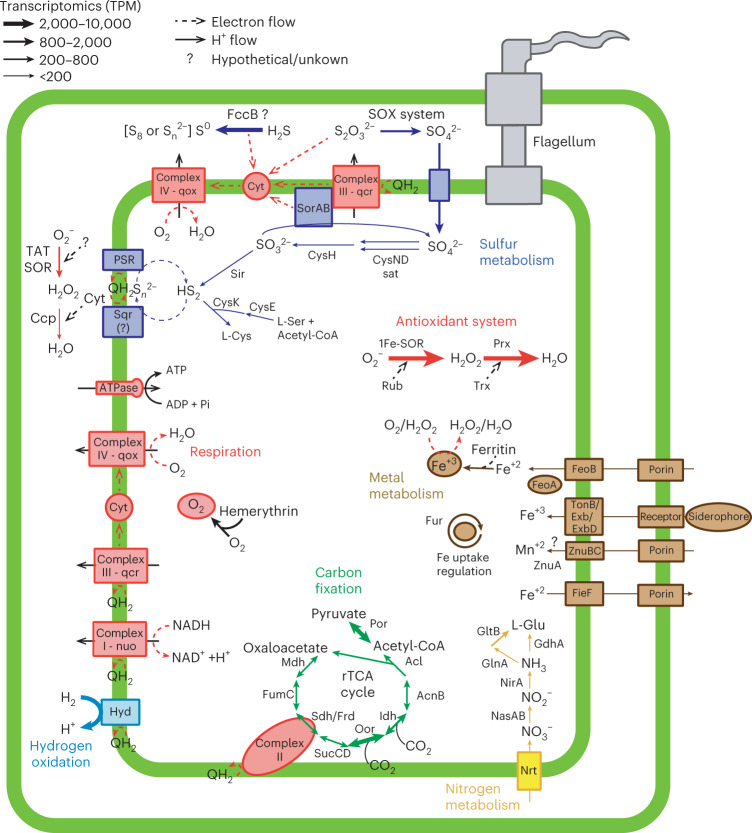
Metabolic map of ^U^*S. pluma*. Metabolic scheme and gene transcription levels of genes involved in aerobic chemolithoautotrophy of ^U^*S. pluma* MAG-1. The average gene expression of Aurora and Polaris plumes is reported as TPM. For enzymes with multiple subunits, the transcription of the catalytic subunit is reported. Steps with more than one arrow indicate that several operons encoding different enzymes catalysing that reaction are present in the genome. Source data

**Extended Data Fig. 6:**
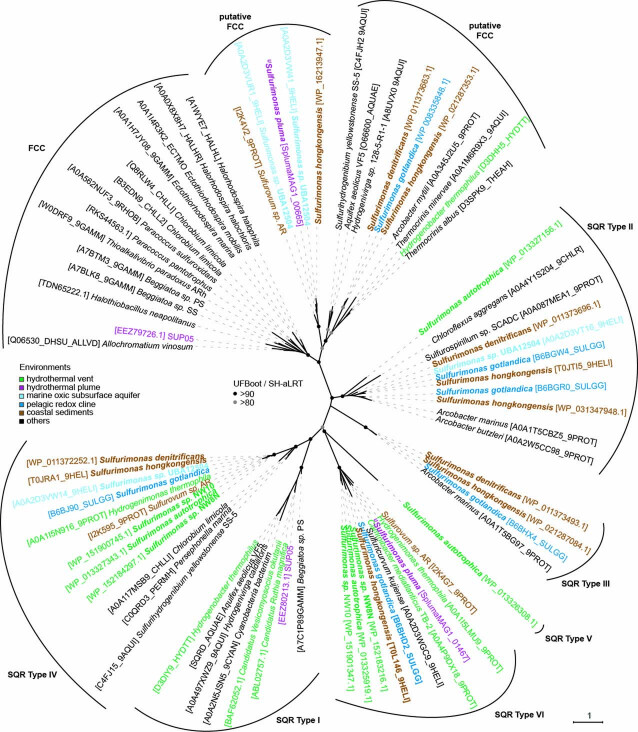
Synteny analysis of operon encoding the OOR and POR enzymes. Gene cluster organization of the five subunits of 2-oxaglutarate-ferrodoxin oxidoreductase and pyruvate-ferrodoxin oxidoreductase in the genomes of ^*U*^*Sulfurimonas pluma*, *Sulfurovum AR*, and Aquificales’ representatives.

The genome of ^U^*S. pluma* contains all genes for the oxidation of zero-valent sulfur (S^0^), or thiosulfate as typically described for the genus *Sulfurimonas*, but it misses the genes for sulfide:quinone reductases (*sqr*), a functional-core gene for this genus (Fig. 3 and Supplementary Table 5). Notably, ^U^*S. pluma* encodes a non-canonical flavocytochrome c sulfide dehydrogenase (*fcc*) that might replace *sqr* in sulfide oxidation (Extended Data Fig. 6 and Supplementary Note 2). At both Gakkel Ridge plumes, the expression of genes for canonical sulfur oxidation was substantially lower compared with that of membrane-bound (NiFe)-hydrogenase (Fig. 2).

In previous studies, reduced manganese and iron species were suggested to be additional potential electron donors for microorganisms in hydrothermal plumes^25^. In the MAGs of ^U^*S. pluma*, we found no evidence for known genes encoding enzymes for extracellular Fe (II) and Mn (II) oxidation, such as outer-membrane embedded cytochrome c (Cyc2 or Cyt572) and porin–cytochrome c protein complexes^39,40^. However, ^U^*S. pluma* encodes and expresses different genes for the uptake and control of the intercellular availability of iron and manganese (Fig. 3). Among them, the ^U^*S. pluma* genomes encode for an iron storage protein such as ferritin, which is absent in the genomes of *S. gotlandica* and *S. autotrophica* (Supplementary Table 5). Ferritin is an intracellular protein that oxidizes excess ferrous iron and can store up to 2,000 non-reactive ferric iron atoms^41^. During this reaction, ferretins also consume cytoplasmic O_2_ and remove H_2_O_2_^42^ (Fig. 3). The high transcription of the ferritin gene in the hydrothermal plume metatranscriptomes of the Gakkel Ridge (Fig. 2) suggests a potential role in mitigating oxidative stress and in iron storage. Future studies should focus on the iron cycling pathways of ^U^*S. pluma* because they may be important elements of the ‘microbial Fe pump’ between hydrothermal vents and the ocean^26^.

### Adaptations to high oxygen concentrations

^U^*S. pluma* differs from cultivated *Sulfurimonas* strains in the lack of genes for nitrate and nitrite reductases and by coding for caa3-type (A2-type) cytochrome c oxidase (Table 1). All other *Sulfurimonas* sp. genomes encode only cbb3-type oxidase^12^, which has a high affinity for oxygen and allows growth at microaerobic conditions^42^. Yet, at higher oxygen concentrations (that is, >20%), the cbb3-type oxidase becomes inefficient, resulting in impaired growth^9,12^. In fact, the cultured *Sulfurimonas* strains grow optimally at an O_2_ concentration of 1–8%, and become inactive at O_2_ concentrations higher than 20%^1–5,9,11^. Moreover, previous studies found *Sulfurimonas* predominantly in environments subject to strong fluctuations in O_2_ concentrations (that is, benthic and pelagic redoxclines^12^; Supplementary Table 3). The cold polar waters studied here are oxygen-saturated and the diluted hydrothermal fluids do not substantially lower their oxygen contents. Hence, ^U^*S. pluma* is permanently exposed to high oxygen concentrations (ca. 300 µM; Supplementary Table 3). We hypothesize that the acquisition of caa3-type (A2-type) cytochrome c oxidase allows an efficient respiration of ^U^*S. pluma* in this fully oxic environments. This cytochrome c oxidase is present in many aerobic bacteria and it has strong homology to the mitochondrial cytochrome oxidase (A1-type)^43^. Of note, within the phylum of Campylobacterota, we found all four subunits of caa3-type oxidase in the genome of *Sulfurovum* sp. AR derived from aerobic Arctic sediments^44^. This oxidase has an amino acid identity of 70% to that of ^U^*S. pluma*. However, this caa3-type oxidase cannot be misassembled in the ^U^*S. pluma* MAGs because *Sulfurovum* sequences are rare in the Gakkel seawater (Supplementary Table 1), and the synteny analysis of contigs encoding for this enzyme points toward an acquisition by horizontal gene transfer (Supplementary Fig. 2).

**Fig. 4:**
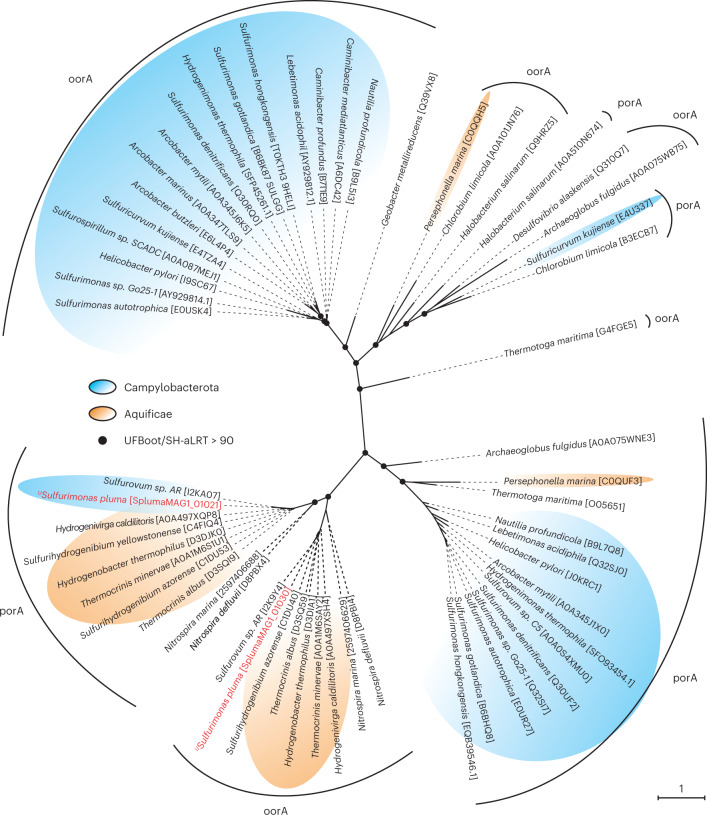
Phylogenetic tree of rTCA cycle ferredoxin oxidoreductases. Phylogenetic relationships between the alpha subunit of 2-oxoglutarate:ferredoxin oxidoreductase (oorA) and the alpha subunit of pyruvate:ferredoxin oxidoreductase (porA). The text in red shows oorA and porA genes of ^U^*S.* *pluma*. The scale bar represents the expected number of changes per amino acid position. UFBoot and SH-aLRT values are based on 1,000 replicates. Best substitution model: LG + R4.

^U^*S. pluma* contains multiple oxygen-sensitive enzymes, electron carriers and enzymes that form reactive oxygen species, hence it requires solutions to reduce intracellular O_2_ concentrations. One of the most effective solutions is aerobic respiration. The cbb3-type and caa3-type are both highly expressed in the plumes (Fig. 2), suggesting that under sustained growth, their activity can be combined to efficiently reduce intracellular O_2_ concentrations and/or simultaneously use multiple electron sources for energy conservation.

**Extended Data Fig. 7:**
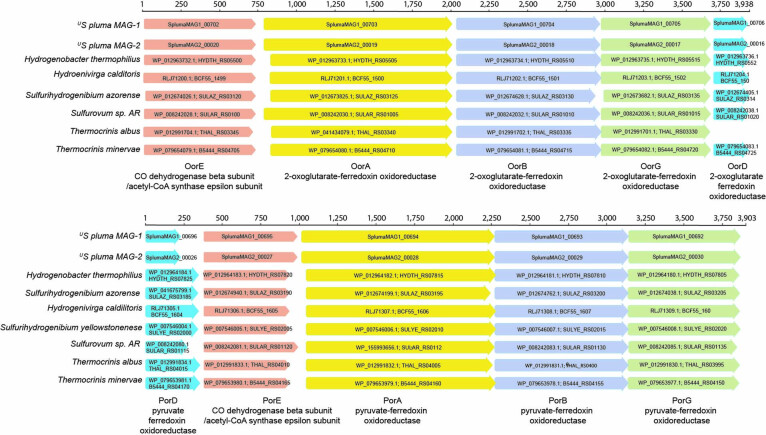
Phylogenetic tree of sulfide oxidizing enzymes. Phylogenetic relationships between the beta subunit of Flavocytochrome c sulfide dehydrogenase (FCC) and Sulfide:quinone oxidoreductase (SQR). The scale bar represents the expected number of changes per amino acids position. UFBoot and SH-aLRT values are based on 1000 replicates. Best substitution model: LG + R4. *Sulfurimonas* species are in bold.

The MAGs of ^U^*S. pluma* contain all genes of the reverse tricarboxylic acid (rTCA) cycle (Fig. 2), which is the common autotrophic carbon fixation pathway of all cultured *Sulfurimonas* species^12^. Its key genes 2-oxaglutarate:ferredoxin oxidoreductase (OOR), pyruvate:ferredoxin oxidoreductase (POR) and ATP-dependent citrate lyase (ACL) were highly expressed, suggesting the rTCA cycle as the main pathway for autotrophic growth (Fig. 3). However, carbon fixation via rTCA usually tolerates only trace amounts of oxygen because OOR and POR are oxygen sensitive^45^. Our study suggests that ^U^*S. pluma* is capable of using rTCA under fully and permanently oxic conditions. Fixing carbon via the rTCA pathway under these conditions requires specific adaptations of the genes involved as recently reported for a marine nitrite-oxidizing bacterium^46^. Indeed, the gene operon encoding the OOR and POR enzymes in ^U^*S. pluma* has five subunits instead of the four subunits present in other *Sulfurimonas* strains (Table 1). The phylogenetic and synteny analysis of this operon showed that the catalytic subunits of OOR (*oorA*) and POR (*porA*) are most closely related with members of the Aquificales and that the operon has the same organization as the members of this bacterial order (Fig. 4 and Extended Data Fig. 7). The Aquificales member *Hydrogenobacter thermophilus* encodes two OOR with either two or five subunits. Under anoxic conditions, it synthesizes the two-subunit form, but when exposed to oxygen, it switches to the five-subunit form. This strategy allows carbon fixation via the rTCA cycle even at 40% oxygen^47,48^. This five-subunit OOR has a lower specific activity compared with two-subunit anaerobic enzymes^47^, which probably requires production of large amounts of this oxygen-tolerant form^49^. Accordingly, the ^U^*S. pluma* genes encoding for OOR and POR subunits were among the most highly expressed genes in the plumes (Fig. 2).

Highly expressed bacterial hemerythrin-like genes may help the activity of oxidases (Fig. 3). Hemerythrins are non-heme intracellular oxygen-binding proteins. The two hemerythrin-like sequences found in genomes of ^U^*S. pluma* have one single hemerythrin domain, similar to those found in other *Sulfurimonas* species (Table 1) and other Campylobacterota and Aquificae^50^. Putative functions of bacterial hemerythrins include oxygen storage, oxygen detection or oxygen detoxification^50^. In motile microaerophilic or anaerobic bacteria, it has been proposed that hemerythrins act as an oxygen sensor for aerotaxis^51,52^, yet there are evidences for oxygen detoxification in microaerophilic Campylobacterota species^52^ and the enhancement of oxygen respiration in methane oxidizers^53^. ^U^*S. pluma* is probably motile, as indicated by the presence and expression of genes for the flagellum (Fig. 3), thus we cannot exclude a sensing role of its hemerythrins.

A further strategy to reduce intracellular O_2_ concentration in hydrothermal plume ^U^*S. pluma* is the loss of superoxide dismutases (Table 1), an enzyme that catalyses the transformation of superoxide (O_2_^−^) into molecular oxygen and hydrogen peroxide^54^. This is the primary antioxidant system in aerobic organisms, and it is also present in all cultivated members of *Sulfurimonas* (Table 1). In ^U^*S. pluma*, the antioxidant system includes superoxide reductase (SOR) and peroxidases (Table 1), which sequentially reduce O_2_^−^ to H_2_O_2_ and then to H_2_O using electrons from other reduced substrates, respectively^54^ (Fig. 3). This system contributes to the decrease in intracellular O_2_ concentration and also consumes reductants in the cytosol, thereby lowering the formation of harmful reactive oxygen species^54^. In addition to the canonical/cytoplasmic core gene for 1Fe-SOR, ^U^*S. pluma* showed high transcription of a gene encoding for a 1Fe-SOR with translocation signal peptide (TAT-1Fe-SOR; Fig. 3), which is not present in any other *Sulfurimonas* genomes (Table 1). The presence of a TAT motif suggests a periplasmic location for this enzyme, and therefore a putative role in scavenging the periplasmic superoxide. This is further evidence for a lifestyle adapted to the oxygen-saturated non-buoyant plumes of deep hydrothermal vents and chemically similar aquatic systems.

## Discussion

In this comparative study of Arctic Ocean and global deep-sea microbiota, we identified an uncharacterized aerobic *Sulfurimonas* species inhabiting non-buoyant hydrothermal plumes and fluids, and subsurface aquifers. The genome of ^U^*S. pluma* shows substantial genome reduction (17−40%), including the loss of genus-specific functional genes (that is, for denitrification) and acquisition of genes allowing growth in pelagic oxygen-saturated environments.

Previous studies described SUP05 as a widespread and dominant chemolithoautotroph in hydrothermal plumes, using both sulfur compounds and hydrogen as energy source^25,55^. The ability of ^U^*S. pluma* and SUP05 to rely on the same substrates for energy to grow and their variable co-occurrence in hydrothermal plumes (Table 2) suggest that they could be competitors, as described for marine pelagic redox gradients^19^. Due to the low amounts of substrates (that is, tens to hundreds of nM) and the lack of a redoxcline in non-buoyant hydrothermal plumes, the mechanisms controlling niche partitioning between *Sulfurimonas* and SUP05 in this environment might be different from those proposed for pelagic redoxclines of the Baltic Sea^19^. Our results suggest hydrogen as an essential energy substrate for the growth of ^U^*S. pluma* in the studied hydrothermal plumes. This agrees with its dominance in other hydrogen- and metal-rich non-buoyant hydrothermal plumes (Table 2). Of note, ‘*Candidatus* Sulfurimonas marisnigri’ could grow at atmospheric oxygen concentrations, but only when MnO_2_ was supplied as a terminal electron acceptor^10^. It remains to be clarified whether metals and other hydrothermal compounds favour ^U^*S. pluma* growth and contribute to niche differentiation, and how ^U^*S. pluma* adapts to growth at temperatures close to the freezing point.

**Table 2:** Comparison of hydrothermal systems and vent fluid types for non-buoyant hydrothermal plumes hosting chemolithotrophic ^U^*S. pluma* and SUP05 cluster (family Thioglobaceae)

Ridge system	Vent field	Vent site	Rock hosting system	Fluid type	^U^*S. pluma*	SUP05	Reference
					%	%	
Mid Atlantic Ridge	Logatchev	Irina I	Ultramafic	Hydrogen-metal-rich	up to 70	<10	^22,135^
	Rainbow	−	Ultramafic	Hydrogen-metal-rich	up to 60	<10	^23,136,137^
SouthWest Indian Ridge	Longqi	DFF1, DFF3, DFF5, DFF6	Basalt	Sulfide-metal-rich	up to 21	<5	^24,138^
Guaymas Basin	−	−	Basalt and organic-rich sediments	Methane-ammonia-rich	<2	30	^34,55^
Gakkel Ridge	Aurora	−	Basaltic/ultramafic	Hydrogen-metal-rich	up to 79^a^	<10^a^	this study^29,58^
	Polaris	−	Basaltic	Hydrogen-rich	<18^a^	up to 40^a^	this study^59^
Kermadec Arc	Macauley, Brothers	−	Submarine volcano	Sulfide-metal-rich	<4	up to 80	^56^

^a^Based on 16S rRNA sequences from metatranscriptomes (Extended Data Fig. 2).

Percentages refer to the proportion of 16S rRNA gene sequences in sample from non-buoyant hydrothermal plumes.

The global presence of a hydrogenotrophic *Sulfurimonas* species in transient environments such as non-buoyant hydrothermal plumes opens new paradigms in the microbial ecology of this and other aquatic habitats. So far, it has been postulated that microbes growing in the plume, such as the sulfur-oxidizing Gammaproteobacteria of the SUP05 clade and mixotrophic SAR324 deltaproteobacteria, are derived primarily from ambient seawater^26,56^. These microorganisms are also abundant and active in other marine pelagic environments (for example, surface and deep ocean, oxygen minimum zones), suggesting that their habitat is not exclusively hydrothermal plume but is widespread in the oceans^25^. Our results showed that non-buoyant hydrothermal plumes are a suitable environment for the growth of microorganisms typically inhabiting hydrothermal vents such as *Sulfurimonas*. We suggest that the hydrothermal plume does not act exclusively as a vector for dispersing microorganisms from benthic hydrothermal environments, but it might also support ecological connections between pelagic and seafloor/subsurface habitats. The phylogenetic analysis suggests that the ^U^*S. pluma* lineage could have been derived from a hydrothermal vent-associated ancestor (probably by sympatric speciation), which acquired higher oxygen tolerance and then spread across the oceans. However, it remains to be further investigated whether ^U^*S. pluma* originates from vent-associated environments or from background seawater. On one hand, the presence of a very similar ribotype (>99.5% 16S rRNA gene sequence similarity) in hydrothermal plumes across the globe (Fig. 1) suggests that the *Sulfurimonas* cluster, including ^U^*S. pluma*, is part of the ocean microbial seed bank, and therefore that background seawater might be the source of ^U^*S. pluma*. On the other hand, it may be that ^U^*S. pluma* enters into the hydrothermal plumes from populations living on seafloor vent-associated environments, which due to oxygen tolerance have a higher dispersal potential than benthic *Sulfurimonas* species, resulting in higher global connectivity^17^. Future studies on uncultivated *Sulfurimonas* species described here will be needed to verify these hypotheses, and to shed light on environmental and ecological forces that shape the connections and composition of microbial communities between different environments such as subsurface aquifers, diffusive flow and hydrothermal plumes.

## Methods

### Sample collection

Water samples were collected in the valley of the Southwest Indian Ridge (SWIR segment 10°–17° E; Atlantic sector), and at the Aurora and Polaris vent sites of the Gakkel Ridge (Central Arctic Ocean) during RV *Polarstern* expeditions PS81 (9 November–16 December 2013)^57^, PS86 (7 July–3 August 2014)^58^ and PS101 (9 September–23 October 2016)^59^, respectively. A conductivity-temperature-depth (CTD) rosette equipped with 24 12-l Niskin bottles was used to collect water samples for DNA extraction at all three sites, and for cell counts at Aurora and Polaris sites. Microbial biomass inside and outside the hydrothermal plumes at the Aurora and Polaris sites was collected by filtering in situ a large volume of seawater (ca. 200 l per sample) using in situ pumps (WTS-LV04; McLane) attached to the CTD-frame and CTD-wire and equipped with polycarbonate filters (142 mm diameter, 0.2 µm pore size; Millipore). The pumps were programmed to operate at maximum pump rate for 90 min. The hydrothermal plume signal was recorded by oxygen, sulfide, turbidity and Koichi-type redox sensors mounted on the CTD, by miniature autonomous plume recorders (MAPR supplied by the PMEL Earth-Ocean observation programme of the National Oceanic and Atmospheric Administration (NOAA)) and by custom-made sensors for temperature, redox, pH and H_2_S mounted on an ocean floor observation system. The typical physico-chemical signatures for hydrothermal vent plumes were not found in the water column of the SWIR segment investigated, yet turbidity and redox anomalies were recorded in bottom water^60^. Non-buoyant hydrothermal plumes were identified both at Aurora^58^ and Polaris^59^ locations. Both plumes contained CH_4_, H_2_ and H_2_S, the major difference being the high abundance of particles (that is, polymetallic sulfides) in the Aurora plume^58^ compared with the Polaris plume^59^. At these sites, seawater samples were collected in the hydrothermal plume and in the surrounding water: above the plume, below the plume, bottom water and background water (that is, seawater without physico-chemical signatures for hydrothermal plume). Seawater samples were also collected at reference stations (that is, not affected by hydrothermal plume) located 2 km (inside the ridge; ‘internal reference’) and 56 km (outside the ridge; ‘external reference’) away from the Aurora field, and 16 km (inside the ridge; ‘internal reference’) and 190 km (outside the ridge; ‘external reference’) away from the Polaris field. The stations are listed in Supplementary Table 6.

### Fluorescence in situ hybridization (FISH)

Seawater samples (ca. 500 ml) were fixed with formaldehyde (2% final concentration) for 8 h at 4 °C and filtered onto 0.22 μm polycarbonate filters (47 mm diameter; Millipore). The filters were washed with sterile-filtered seawater and with 70% ethanol in MQ-water, dried and stored at −20 °C until further processing. Catalysed reporter deposition FISH (CARD-FISH) was performed according to ref. ^61^. For the hybridization of ribosomal RNA, the oligonucleotide EPSY914 5′–GGTCCCCGTCTATTCCTT–3′ (35% formamide^62^; synthetized by Biomers) was used to target members of the *Campylobacterota* phylum, and the oligonucleotide NON338 5’–ACTCCTACGGGAGGCAGC–3’ (synthetized by Biomers) was used as negative control (35% formamide)^63^. All hybridizations were conducted for 2.5 h at 46 °C. Cells were imaged at ×1,000 magnification with an epifluorescence microscope (Axiophot II Imaging, Zeiss). Cells were counter-stained with 4′,6-diamidino-2-phenylindole (DAPI; Sigma-Aldrich) and for each filter, a minimum of 800−1,000 DAPI-stained cells from 20−30 different fields of view were counted.

Oligonucleotide probes specific for ^U^*S. pluma* were designed on the basis of sequences retrieved through amplicon sequencing of the hypervariable V3−V4 region of the 16S rRNA gene of samples collected at the Aurora vent site and using the ARB software package^64^. The Probe-Match function of ARB was used to test in silico the coverage and specificity of the probes against the SILVA rRNA reference database (release 132)^65^ complemented with 16S rRNA gene sequences from this study. Almost all the *Sulfurimonas* sequences clustered into two groups (within similarity >99%), representing the *Sulfurimonas* OTU1 and OTU2 identified by the analysis of 16S rRNA gene amplicon sequences (described in the section ‘Illumina 16S rRNA gene sequencing’). We designed specific probes for OTU1 (SLFM-A484 5’–GCTTATTCATAGGCTACC–3’; 15% formamide) and OTU2 (SLFM-B484 5’–GCTTATTCATATGCTACC–3’; 20% formamide), both synthetized by Biomers. Due to the high similarity between these two oligonucleotides (one mismatch for G and T), each probe was used in a mix together with the other (non-labelled) probe as competitor oligonucleotide. To check the coverage and specificity of ^U^*S. pluma*’s probes in the environmental samples, double CARD-FISH hybridizations were carried out using the *Campylobacterota* probe (EPSY914) as a positive control.

### DNA and RNA extraction

Seawater samples for 16S rRNA gene analysis were filtered immediately after the retrieval of the Niskin bottles. The filtration was carried out in a temperature-controlled room (2 °C) in the dark and did not exceed 1−1.5 h. Three liters of seawater were filtered onto a 0.22 μm polycarbonate filter (47 mm diameter; Millipore, Merck) during PS81 and PS86 using a vacuum pump (N 022 AN.18; KNF), and 10 liters through a 0.22 μm Sterivex filter (Millipore, Merck) during PS101 using a peristaltic pump (Masterflex; Cole Parmer). All filters were stored and transported at a temperature between −20 and −80 °C. Genomic bacterial DNA was isolated in a combined chemical and mechanical lysis procedure using the PowerWater DNA Isolation kit (MO BIO Laboratories). Before DNA isolation, the Sterivex cartridges of the 0.22 μm membranes were cracked open to place the filters in the kit-supplied bead beating tubes. The isolation was continued according to the manufacturer’s instructions, and genomic DNA was stored at −20 °C.

DNA and RNA for metagenomic and metatranscriptomic analyses, respectively, were extracted from the microbial biomass retrieved with the in situ pumps. After the recovery of the pumps, filters were immediately cut into 6 pieces, transferred to a screw-cap tube, frozen in liquid nitrogen for 5 min, and then stored and transported at −80 °C. RNA was extracted from three sections of filters in an RNase-free tube with the mirVana mRNA Isolation kit (Ambion). RNA extracts were treated with the TURBO DNA-free kit (Ambion) to remove co-extracted DNA, and purified and concentrated using the RNeasy MinElute kit (Qiagen). RNA extracted from each filter section was pooled together and stored at −80 °C. The genomic DNA was extracted from three pieces of the filter using the PowerWater DNA Isolation kit (MO BIO Laboratories) following the manufacturer’s instructions. The extracted genomic DNA from each filter was pooled and stored at −20 °C.

### Illumina 16S rRNA gene sequencing

The hypervariable V3−V4 region of the bacterial 16S rRNA gene was amplified using bacterial primers S-D-Bact-0341-b-S-17 (5′-CCTACGGGNGGCWGCAG-3′) and S-D-Bact-0785-a-A-21 (5′-GACTACHVGGGTATCTAATCC-3′)^66^. Sequences were obtained on the Illumina MiSeq platform in a 2 × 300 bp paired-end run aiming for >50,000 reads per sample (CeBiTec), following the standard instructions of the 16S metagenomic sequencing library preparation protocol (Illumina). The workflow and scripts used in this study for the quality cleaning, merging, clustering and annotation of the sequences can be found in ref. ^67^. Briefly, only reverse and forward reads with quality score higher than 20 (applying a sliding window of 4) were merged, clustering of sequences into OTUs was done using the programme swarm (v2.2.2)^68^, and the taxonomic classification was based on the SILVA rRNA reference database (release 132)^65^.

### Metatranscriptomes

RNA quantification, library preparation and sequencing were carried out at CeBiTec. Total RNA quantity and integrity were assessed using the Aligent 2100 bioanalyzer with the Prokaryote total RNA Pico assay (Agilent RNA 6000 Pico kit). Only RNA samples with an integrity number >7 were used for sequencing. The TruSeq Stranded Total RNA kit (Illumina) was used for RNA library preparation. The rRNA depletion step was omitted. Of the total RNA, 80 ng (in 5 μl volume) was combined with 13 μl of ‘Fragment, Prime and Finish mix’ for the RNA fragmentation step according to the Illumina TruSeq stranded mRNA sample preparation guide. Subsequent steps were performed as described in the sample preparation guide. The library was sequenced on a HiSeq1500 platform (Illumina) in a 1 × 150 bp single-end run generating >20 million reads per sample. The resulting reads were pre-processed, including removal of adaptors and quality trimming (slidingwindow:4:21 minlen:100) using bbduk v34 from the BBMAP package^69^ and Trimmomatic software v0.35^70^, respectively. The trimmed reads were sorted into ribosomal RNA (rRNA) and non-ribosomal RNA (non-rRNA) reads using SortMeRNA software v2.0^71^. A random subset of 1 million rRNA reads per sample was taxonomically classified with phyloFlash software v3.0 beta 1^72^ based on the SILVA database (release 132)^65^.

### Oligotyping of *Sulfurimonas* 16S rRNA amplicon sequences

In addition to the data presented in this study, *Sulfurimonas* V3−V4 16S sequences were obtained from previous studies conducted in environments likely to host *Sulfurimonas* sp. bacteria (Supplementary Table 7). We searched the European Nucleotide Archive (ENA) and PubMed using the following search strategies: (1) The accession numbers of all amplicon datasets generated on the Illumina platform (paired-end) from ENA were extracted, filtering the results for ecological metagenomes from coastal and marine regions likely to contain *Sulfurimonas* sp. (that is, redoxcline environments; accessed 5 March 2020). (2) PubMed was searched using the keywords ‘Sulfurimonas, bacteria, 16S sequencing, Illumina’, filtering the results on the basis of the amplified region. (3) Further unpublished datasets were directly obtained from the authors (Supplementary Table 7).

Given the inconsistent state of the sequences obtained from public archives or the authors directly, several bioinformatic pre-processing steps were necessary to ensure a uniform data set as input for oligotyping. If required, sequences were demultiplexed and primer-clipped using cutadapt v1.9.1^73^. Paired-end sequences were merged with PEAR v0.9.6^74^ and quality trimmed with Trimmomatic v0.35^70^ using a sliding window of 4 bp with an average quality of 15. Only sequences between 380 bp and 450 bp that did not contain any ambiguous bases were retained. If minor modifications affecting at maximum 2 bp of the V3−V4 primers used here were employed in the previous studies, the resulting amplicons were trimmed to matching start and end positions, assuming that any primer bias regarding *Sulfurimonas* would be negligible.

Before oligotyping via minimum entropy decomposition (MED)^33^, non-*Sulfurimonas* sequences were filtered out using a blastn search against a custom *Sulfurimonas* database consisting of 246 curated full-length 16S *Sulfurimonas* sequences obtained from the SILVA reference database (v138)^65^ and assembled from the metagenomes presented in this study. Sequences with an alignment identity of at least 87%, an alignment length of at least 380 bp, a query start at or before position 10, a query end at or after position 380, a subject start between 280 and 380, and a subject end at or after position 690 were retained. An additional taxonomic cross-check was conducted with SINA v1.2.11^75^ using the full SILVA reference database (v138)^65^. To reduce computational requirements, sequences were first clustered with Swarm v2.2.2^68^ and only the seed sequence of each swarm was taxonomically classified. Only sequences associated with swarms whose seed representative was classified as *Sulfurimonas* at a minimum alignment quality of 90 were used for oligotyping.

MED uses the information content (entropy) at each base position in a sequence alignment to partition the data set into oligotypes^33^. It is therefore able to resolve even single nucleotide differences between closely related sequences if these are likely to originate from a biological signal and not from random noise. Here, MED was used to identify oligotypes among the *Sulfurimonas* sequences generated from samples collected worldwide. MED (decompose v2.1) was run with the following parameters using default values if not otherwise indicated: minimum entropy of 0.0965 (-m), one discriminant for decomposition (-d), a minimum substantive abundance of 50 (-M) and with outlier relocation (-R). After MED, the oligotype representative sequences were again aligned against the SILVA reference database (v138)^65^ and only oligotypes classified as *Sulfurimonas* were retained for the subsequent analysis.

Before we further explored the diversity of *Sulfurimonas* 16S amplicons and attempted the detection of environment-specific *Sulfurimonas* ecotypes, we excluded samples where *Sulfurimonas* amplicons were likely to constitute a contamination from adjacent environments, that is, we assumed that in substrate-associated and water samples, proportions of *Sulfurimonas* 16S sequences lower than 0.1% and 1%, respectively, constituted a contamination. Additionally, samples with less than 1,000 sequences in total were excluded from subsequent analyses to avoid bias caused by the low sequencing depth. A total of 308 samples with 2,082,428 sequences represented in 1,389 *Sulfurimonas* oligotypes were retained for further analysis. A full list of samples, their total and *Sulfurimonas* sequence numbers, and number of *Sulfurimonas* oligotypes is included in Supplementary Table 4.

To identify ecotypes, samples were categorized by salinity (marine, brackish, fresh water), zone (pelagic, benthic, subsurface), water depth (coastal, deep-sea), hydrothermal vent influence and artificial environment. On the basis of these categories, the samples were grouped in 11 environments (Supplementary Table 8).

Ecotypes were defined as oligotypes occurring in at least 50% of the samples of a specific group, with 90% of the values in any of the outgroups being lower than half of the 10% quantile of the non-zero sequence proportions in the ingroup. Gamma diversity of *Sulfurimonas* oligotypes was assessed as rarefaction curve of oligotype numbers depending on sampling effort. For each possible number of samples, 100 samples were randomly selected to determine oligotype numbers.

### Metagenome sequencing, assembly and binning

Paired-end libraries were prepared with the TruSeq DNA PCR-Free Sample Prep kit (Illumina) and sequencing of libraries was performed on a MiSeq instrument (Illumina; 2 × 300 bp paired reads) using the v3 sequencing chemistry (CeBiTec). Reads had adapters and contaminants removed with the bbduk tool of the BBMap suite (k = 27; mink = 12)^69^ and trimmed with Trimmomatic (slidingwindow:4:20; minlen:100; v0.35)^70^. Forward and reverse reads from 12 metagenomes (Supplementary Table 6) were de novo assembled following two strategies: (1) co-assembly with MEGAHIT (‘–min-contig-len 1000’; v1.1.2)^76^ and (2) individual assembllies with metaSPAdes (‘–meta’) within the SPAdes suite (‘-k 21,33,55,77,99,127’; v3.9.0)^77^. Reads were mapped and indexed using bwa (v0.7.12)^78^ and SAMtools (v1.5)^79^, and the contigs were binned with CONCOCT (v1.1.0)^80^. A total of 661 bins were de-replicated using dRep (v2.3.2)^81^ on the basis of Mash distance^82^ and genome-wide ANI^83^. Only 7% of the bins passed length (>50 kpb), completeness (>75%) and redundancy (<25%) filtering, and a total of 19 de-replicated bins (ANI > 99%) were obtained. *Sulfurimonas* bins were identified and refined using Anvi’o interactive interface (v6.2)^84^ after the Anvi’o contig database was built to calculate *k*-mer frequencies to identify open reading frames using Prodigal (v2.6.3)^85^ and single-copy genes using HMMER (v3.2.1)^86^, and to classify the bins on the basis of single-copy gene taxonomy of GTDB^87^ using DIAMOND (v0.9.14)^88^. Sequences of 16S rRNA genes were extracted with RNAmmer (v1.2)^89^. Refined *Sulfurimonas* bins were repeatedly re-assembled using BBmap (99% similarity) and SPAdes, removing contigs smaller than 1 kb after each re-assembly step to extend contigs and reduce the size of genome gaps. Completeness and redundancy of the final *Sulfurimonas* MAGs were evaluated using CheckM (v1.2.1; based on 104 bacterial single-copy genes)^90^, CheckM2 (v0.1.3; based on machine learning algorithm)^91^ and BUSCO (v5.2.2; based on 628 Campylobacterales single-copy genes)^92^. The number of transfer RNAs was identified using ARAGORN (v1.2.36)^93^. We obtained two almost complete *Sulfurimonas* MAGs, named MAG-1 and MAG-2 (Supplementary Table 2). These two MAGs represent consensus MAGs, which are based on 16 individual bins produced from different environmental samples. Proteins from the final *Sulfurimonas* MAGs were predicted and annotated using Prokka (v1.11)^94^. The Prokka-predicted proteins were additionally annotated with Pfam (release 30)^95^ and TIGRFAM (release 14)^96^ profiles using HMMER searches (v3.1b2)^86^ and by the identification of KEGG Orthology numbers with the GhostKOALA webserver^97^. The proteins were also assigned to clusters of orthologous groups (COGs)^98^ using the software COGsoft (v4.19.2012)^99^ and transmembrane motifs were identified using TMHMM (v2.0)^100^. On the basis of the various annotation tools, the annotation of proteins of specific interest was manually refined. The sequences of hydrogenases were classified using HydDB^101^. Iron-related genes were identified using FeGenie’s tool and database^102^. RedoxyBase^103^ and SORGOds^104^ were used to identify classes of peroxidase and types of superoxide reductase, respectively.

The quality-filtered short metatranscriptomic reads were mapped to annotated MAG-1 and quantified using the programme kallisto (v0.43.1)^105^. To compare expression levels between genes, gene expression is reported as transcripts per million (TPM)^106^, while the effective counts were used for the differential expression of MAG-1 genes between Aurora and Polaris plumes.

### Gene prediction from metagenomic assemblies

The genes were predicted on contigs using Prodigal (v2.6.3)^85^ and the proteins clustered using MMseq2 (v13.45111;–min-seq-id 0.95 -c 0.95–cov-mode 1–cluster-mode 2)^107^. Proteins with length <50 amino acids were removed (ca. 15% of total proteins). The functional annotation of proteins was conducted using KEGG^108^ (release 101.0; with diamond v2.0.15^88^), Pfam (v35.0^109^; with hmmsearch HMMER v3.3.2^86^) and NCycDB^110^, a curated database for nitrogen cycling genes (with diamond v2.0.15^88^). Taxonomic affiliation of the contigs was done with CAT (v5.2.3)^111^ using GTDB (release 207)^87^ as reference database. The metagenomic and metatranscriptomic reads were mapped to the gene sequences using bwa-mem2 (v2.2.1)^112^ and converted to counts per gene with htseq-count script from HTSeq (v2.0.2)^113^. On average, 80% and 87% of reads from Polaris and Aurora metagenomes, respectively, were retrieved by the catalogue of genes.

### Phylogeny

The backbone phylogenetic tree for 16S rRNA gene sequences was calculated using *Sulfurimonas* sequences from SILVA SSU r138 RefNR (*n* = 19), *Thiomicrospira* sp*.* CVO (original name for a *Sulfurimonas denitrificans* strain) from SILVA SSU r138 RefNR (U46506)*, Sulfurimonas crateris* (MK859925), Gakkel Ridge plume *Sulfurimonas* MAGs (this study) and closest related 16S rRNA gene sequences to ^U^*S. pluma* MAGs (blastn hits filtered by identity >98 and coverage >97%: JN874148.1 and JN874176.1; GeneBank nucleotide; accessed May 2020). The sequences of *Sulfuricurvum kujiense* from SILVA SSU r138 RefNR (*n* = 3) were used as outgroup. Sequences were aligned with MAFFT using the L-INS-i method with default settings^114^, and the alignment was cleaned with BMGE with default setting^115^. Both programmes were used on the Galaxy platform^116^. A maximum-likelihood-based tree was constructed using W-IQ-TREE^117^, first searching for the best substitution model^118^ before evaluating branch support using 1,000 ultrafast boostrap (UFBoot) and SH-aLRT branch test replicates. Evolutionary placement algorithm (EPA) in RAxML (v8.2.4)^119^ was applied to add 253 partial *Sulfurimonas* 16S rRNA gene sequences (250−1,400 bp retrieved from GenBank nucleotide database; data accessed May 2020) to the tree without changing its topology. Further partial 16S rRNA gene sequences of *Sulfurimonas* sp. obtained from previous next-generation sequencing studies conducted in deep-sea hydrothermal fluids (JAH_MCR_Bv6_MCR_CTD03_08; JAH_AXV_Bv6v4_FS788; downloaded from vamps.mbl.edu) and plumes (PRJEB36848; SRP016119; PRJNA638507) were likewise added to the tree.

Phylogenetic trees for functional genes (that is, alpha subunit of 2-oxoglutarate:ferredoxin oxidoreductase, alpha subunit of pyruvate:ferredoxin oxidoreductase, beta subunit of flavocytochrome c sulfide dehydrogenase and sulfide:quinone oxidoreductase) were constructed following the workflow described for the backbone 16S rRNA gene tree, with amino acids as coding sequence and MAFFT alignment method set to ‘auto’. Sequences were obtained from GenBank nucleotide and UniProt databases (data accessed May 2020). The sulfide:quinone oxidoreductase sequence of ^U^*S. pluma* was added to the backbone tree with EPA. All phylogenetic trees were visualized and refined with iTOL^120^.

### Comparative genomic analysis

We used Anvi’o v6.2^84^ as the main tool to carry out comparative genomic analysis, and the overall workflows applied in this section can be found at http://merenlab.org/2016/11/08/pangenomics-v2/, http://merenlab.org/2017/06/07/phylogenomics/ and http://merenlab.org/data/prochlorococcus-metapangenome/.

### Pangenomic analysis

We retrieved 26 *Sulfurimonas* and 2 *Sulfuricurvum* genomes (completeness >85% and redundancy <5%) from GenBank (accessed January 2020). Supplementary Table 9a reports information for each isolate genome and MAG. DNA and amino acid sequences of the genomes, including ^U^*S. pluma* MAG-1 and MAG-2, were stored in an Anvi’o’s genome database (programme ‘anvi-gen-genomes-storage’). From the genome database, we computed the pangenome to identify the gene clusters (programme ‘anvi-pan-genome’) representing sequences of one or more predicted open reading frames (Prodigal v2.6.3)^85^ grouped together on the basis of their homology at the translated DNA sequence level. For multiple sequences alignments, Anvi’o used MUSCLE (v3.8.1551)^121^, the MCL algorithm to identify clusters in amino acid sequence similarity^122^ and the programme ‘anvi-run-ncbi-cogs’ to annotate genes with functions by searching them against the COG database (October 2019 release)^98^ using blastp v2.9.0+^123^. ANI was computed for all *Sulfurimonas* species and MAGs representative for different environments (that is, hydrothermal vent and plume, marine pelagic, marine oxic aquifer, costal and terrestrial) with the anvi’o programme ‘anvi-compute-genome-similarity’.

### Phylogenomic analysis

We visualized the distribution of gene clusters across genomes with ‘anvi-display-pan’ and manually selected 258 SCGs present in all genomes. Concatenated amino acid sequences and partition file for SCG were extracted with ‘anvi-get-sequences-for-gene-clusters’ and used for phylogenomic analysis. The phylogenomic tree was built with IQ-Tree (v2.0)^124^, first identifying the partition substitution models^125^ and then constructing a consensus tree using a maximum-likelihood approach with 1,000 UFBoot branch test replicates.

### Metapangenomic analysis

Here we refer to ‘metapangenome’ as defined in ref. ^126^, that is, ‘the outcome of the analysis of pangenomes in conjunction with the environment where the abundance and prevalence of gene clusters and genomes are recovered through shotgun metagenomes’. We have also extended the analysis to include gene cluster expression as retrieved through shotgun metatranscriptomes. The Anvi’o pangenome was computed as described above but including only *Sulfurimonas* isolate genomes and ^U^*S. pluma* MAG-1, and using the programme ‘anvi-import-functions’ to import annotations from other databases, including Pfam^95^, eggNOG^127^, TIGRFAM^96^ and the script by E. Graham (https://github.com/edgraham/GhostKoalaParser) to import GhostKOALA/KEGG annotations^97^. To investigate the environmental distribution and gene transcription patterns of ^U^*S. pluma* MAG-1, metagenomic and metatranscriptomic reads from hydrothermal plumes and benthic vent environments were mapped to MAG-1 and reads recruitment information was imported to the Anvi’o pangenome. To test the specificity of read recruitments, the genomes from isolated *Sulfurimonas* species were also included in the metapangenomic analysis. We downloaded hydrothermal vent (that is, plume, seawater, fluid, chimney, mineral deposit and sediment; *n* = 63) and background seawater (*n* = 3) metagenomes from ENA (data accessed February 2020; Supplementary Table 9b). Hydrothermal plume (*n* = 2), seawater (*n* = 6), fluid (*n* = 6) and background seawater (*n* = 2) metatranscriptomes were downloaded from ENA (data accessed May 2020) and the metagenomics RAST server (MG-RAST; data accessed May 2020; Supplementary Table 9b). Low-quality reads were removed from metagenomes and metatranscriptomes with Trimmomatic (slidingwindow:4:20 and minlen:65; v0.35)^70^. The quality-filtered short metagenomic reads were mapped to the concatenated *Sulfurimonas* genomes and ^U^*S. pluma* MAG-1 with Bowtie2 (default setting and ‘–no-unal’ flag; v2.3.2)^128^. An Anvi’o database was created for storing contigs information (DNA sequence, GC content, tetranucleotide frequency and open reading frames) of *Sulfurimonas* genomes and ^U^*S. pluma* MAG-1 with the programme ‘anvi-gen-contigs-database’. Then the programme ‘anvi-profile’ was used to process BAM files and generate an Anvi’o profile database that contained the coverage statistics of each *Sulfurimonas* genome in a given metagenome, summarizing gene coverage values across metagenomes and percentage read recruitment per genome with ‘anvi-summarize’. This percentage refers to the contribution of reads recruited by a genome to total reads recruited by all genomes for each metagenome. The table was then imported to the *Sulfurimonas* pangenome in Anvio’o using the programme ‘anvi-import-misc-data’ as ‘layers’. The quality-filtered short metatranscriptomic reads were mapped to ^U^*S. pluma* MAG-1 gene clusters (extracted from Anvi’o’s *Sulfurimonas* pangenome with ‘anvi-get-sequences-for-gene-clusters’) with the programme kallisto (v0.43.1)^105^. The expression of gene clusters is reported in TPM, correcting for the effective length of the transcript. The TPM of ^U^*S. pluma* MAG-1 genes were imported to the *Sulfurimonas* pangenome using the programme ‘anvi-import-misc-data’ as ‘items’.

### Statistical analysis

All statistical analyses were conducted in R using the core distribution (v3.5.2)^129^, the package vegan^130^ for ecotype analysis and the package edgeR^131^ for testing differences in the genes’ expression between Aurora and Polaris plumes, applying false discovery rate (FDR) criterion proposed by Benjamini and Hochberg^132^. Unless specified otherwise, plots were made using ggplot2^133^ and pooled, refined and labelled with Adobe Illustrator CS5.

### Reporting summary

Further information on research design is available in the Nature Portfolio Reporting Summary linked to this article.

## Supplementary information

Supplementary Information Supplementary Figs. 1 and 2, Tables 1–9, and Notes 1 and 2.

Reporting Summary

## Data Availability

The sequences generated in this study and the ^U^*Sulfurimonas pluma* genomes have been deposited in the European Nucleotide Archive (ENA) at EMBL-EBI under Bioproject PRJEB48226. All the sequences were archived using the data brokerage service of the German Federation for Biological Data (GFBio)^134^. The accession numbers for raw sequences and ^U^*S. pluma* MAGs are listed in Supplementary Table 6 and Supplementary Table 2, respectively. *Sulfurimonas* V3-V4 16S rRNA gene sequences were extracted from metabarcoding studies obtained from ENA (https://www.ebi.ac.uk/ena/browser/home), and the studies and sequences accession numbers are reported in Supplementary Tables 4 and Table 7. *Sulfurimonas* and *Sulfuricurvum kujiense* full-length 16S rRNA gene sequences used in this study were obtained from SILVA RefNR database (v138; https://www.arb-silva.de) and NCBI Genebank (https://www.ncbi.nlm.nih.gov/nucleotide; JN874148.1 and JN874176.1). Partial *Sulfurimonas* 16S rRNA gene sequences (250−1,400 bp) were retrieved from NCBI GenBank (https://www.ncbi.nlm.nih.gov/nucleotide; data accessed May 2020), and from metabarcoding studies obtained from ENA (https://www.ebi.ac.uk/ena/browser/home) and VAMPS (vamps.mbl.edu). Sequences and studies accession numbers are reported in Fig. 1. Functional gene sequences used for phylogenetic tree analysis were obtained either from UniProt (https://www.uniprot.org/uniprotkb?query=*; data accessed May 2020) or NCBI GenBank (https://www.ncbi.nlm.nih.gov/nucleotide; data accessed May 2020), and their accessions are provided in Fig. 4 and Extended Data Fig. 6. *Sulfurimonas* and *Sulfuricurvum* genomes used in this study are available via NCBI GenBank (https://www.ncbi.nlm.nih.gov/nucleotide; data accessed January 2020) and accessions are reported in Supplementary Table 9a. Metagenomic and metranscitptomic data used in metapangenomic analysis were obtained either from ENA (https://www.ebi.ac.uk/ena/browser/home; data accessed May 2020) or MG-RAST (https://www.mg-rast.org; data accessed May 2020), and study accession number is provided in Supplementary Table 9b. Source data are provided with this paper.

## Source data

Source Data Fig. 2 Statistical source data.
Source Data Fig. 3 Statistical source data.
Source Data Extended Data Fig. 1 Statistical source data.
Source Data Extended Data Fig. 2 Statistical source data.
Source Data Extended Data Fig. 3 Statistical source data.
