# Supplementary material for: A hydrogenotrophic Sulfurimonas is globally abundant in deep-sea oxygen-saturated hydrothermal plumes
Source: Nat Microbiol. 2023 Mar 9;8(4):651–65. doi: 10.1038/s41564-023-01342-w (PMC10066037; doi:10.1038/s41564-023-01342-w)
Supplement: Supplementary file 1 — Supplementary Figs. 1 and 2, Tables 1–9, and Notes 1 and 2. [file 41564_2023_1342_MOESM1_ESM.pdf]

nature microbiology

Article

https://doi.org/10.1038/s41564-023-01342-w

A hydrogenotrophic *Sulfurimonas* is globally abundant in deep-sea oxygen-saturated hydrothermal plumes

In the format provided by the authors and unedited

Supplementary Information

Supplementary Figures

Supplementary Fig. 1. Number of detected *Sulfurimonas* oligotypes depending on sampling effort (gamma diversity). The rarefaction curve was calculated based on selecting the specified number of samples 100x (if applicable) at random from the data set and counting the *Sulfurimonas* oligotypes in each of these subsets. The range of the results of the random subset is provided as boxplot. Boxplots show median (central black horizontal line) and interquartile range (upper and lower bounds of box). Whiskers show minimum and maximum excluding outliers (separate points), which were defined as being more than 1.5x the interquartile range removed from the upper or lower bound of the box.

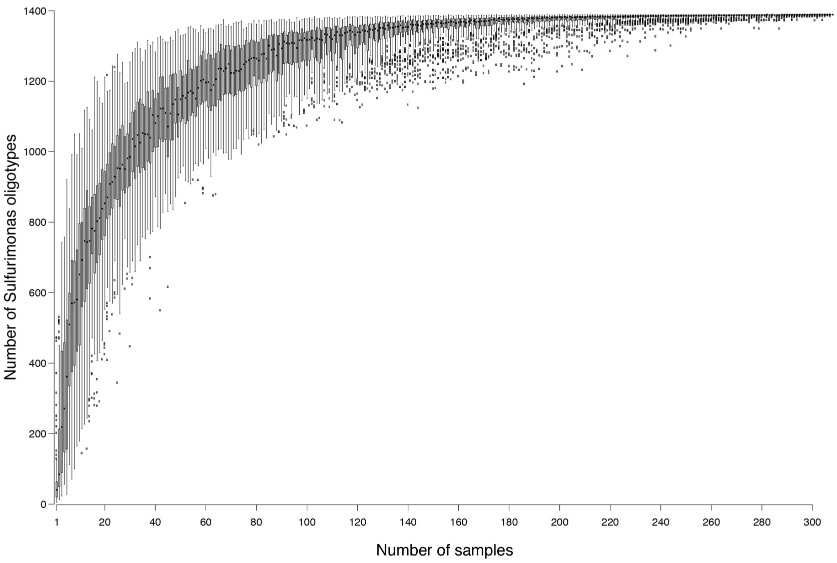

Supplementary Fig. 2. Syntenic gene analysis between ^{U}*S. pluma*'s contig containing caa-3 operon (ctaBCDEFG) and genomes of *S. authotrophica* and *Sulfurovum sp. AR*. The black bar indicates aligned region of the genomes. In red the genes that better aligned between ^{U}*S. pluma* and *Sulfurovum sp. AR*., in green the genes that better aligned between ^{U}*S. pluma* and *S. authotrophica*. The genome alignment has been carried out using the software package *Mauve* within the program *Geneious Prime*® 2022.1.1.

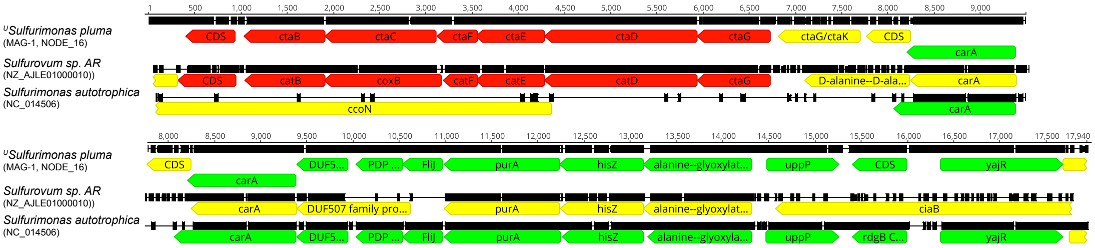

Supplementary Tables

Supplementary Table 1. Number of sequences (seq. n.) of Campylobacterota genera and their contribution to the total number of Campylobacterota sequences (%). a, based on 16S rRNA gene amplicon sequencing (Illumina MiSeq). b, based on 16S rRNA reads from metratrasciptomes (Illumina HiSeq). In parentheses the water depths' range and number of stations (n) are reported for different water types. un. = unclassified.

a

	SWIR		Gakkel Ridge: Aurora vents field								Gakkel Ridge: Polaris vents field									
	Background (2500-4320 m; n = 8)		Reference (2500-3200 m, n = 3)		Above plume (2000-2900 m, n = 4)		Plume (3360-3575 m, n = 5)		Below plume (3500-3855m, n = 4)		Reference (3042-3054 m, n =2)		Above plume (2336-2492 m, n = 4)		Plume (2574-2846 m, n = 8)		Below plume (2870-3107 m, n = 4)		Bottom water (3136-3282 m, n = 4)	
	seq. n.	%	seq. n.	%	seq. n.	%	seq. n.	%	seq. n.	%	seq. n.	%	seq. n.	%	seq. n.	%	seq. n.	%	seq. n.	%
Sulfurimonas	683-10505	> 99.5	1021-13446	> 99.7	1031-14050	> 99.6	6892-31195	100	5859-8966	100	34-42	66-77	0-38	0-88	854-8329	91-99	79-565	39-97	402-606	52-56
OTU1	551-8767	80-88	663-8137	61-71	474-7785	46-55	4432-19849	62-65	3704-5778	63-65	14-19	30-32	0-5	0-13	130-1115	12-17	33-305	13-26	232-331	28-32
OTU2	119-1618	11-17	357-5282	29-39	557-6226	44-54	2456-11281	35-38	2141-3179	34-37	18-23	36-41	0-37	0-86	724-6999	77-83	45-237	18-81	165-261	20-24
Arcobacter	1-22	< 0.5	1-7	< 0.3	3-5	< 0.4	1-5	< 0.1	2-4	< 0.1	9-22	21-34	0-11	0-78	54-86	1-9	8-620	3-61	363-487	44-48
Caminibacter	0	0	0	0	0-1	< 0.1	0-2	< 0.1	0-1	< 0.1	0	0	0	0	0-4	< 0.1	0-1	< 0.1	0-2	< 0.1
Sulfurospirillum	0	0	0	0	0	0	0	0	0	0	0-1	0-2	0	0	0	0	0	0	0	0
Nitratifractor	0	0	0	0	0	0	0	0	0	0	0	0	0	0	0-1	< 0.1	0	0	0-1	< 0.1
Hydrogenimonas	0	0	0	0	0	0	0	0	0	0	0	0	0	0	0-2	< 0.1	0	0	0	0

b

	Gakkel Ridge: Aurora vents field						Gakkel Ridge: Polari vents field							
	Above plume (2478-2690 m, n =2)		Background (3182 m, n =1)		Plume (2958-3183 m, n = 3)		Reference (2070-4841 m, n =8)		Above plume (2425 m, n = 2)		Plume (2635-2645 m, n = 6)		Bottom water (3360-3390 m, n = 4)	
	seq. n.	%	seq. n.	%	seq. n.	%	seq. n.	%	seq. n.	%	seq. n.	%	seq. n.	%
Sulfurimonas	99911-114270	100	202814	100	245047-240251	100	23-1841	8-98	1729-17498	95-98	31711-65036	98-99	2299-2893	35-40
Arcobacter	60-110	< 0.1	96	< 0.1	55-83	< 0.1	23-2359	1-91	69-366	2-4	404-673	1-2	3990-4189	57-63
Sulfurovum	89-135	< 0.1	201	< 0.1	223-246	< 0.1	1-5	0-1	6-14	< 0.1	21-41	< 0.1	129-142	2
Sulfurospirillum	4-7	< 0.1	7	< 0.1	6-26	< 0.1	0-5	< 0.1	0-1	< 0.1	0-3	< 0.1	7-10	< 0.1
Campylobacter	6-11	< 0.1	12	< 0.1	10-23	< 0.1	0	0	0	0	0	0	0	0
Sulfuricurvum	6-8	< 0.1	9	< 0.1	6-10	< 0.1	0	0	0	0	0	0	0	0
Caminibacter	0	0	0	0	0	0	0-3	< 0.1	0-23	< 0.1	19-54	< 0.1	0-3	< 0.1
Hydrogenimonas	0	0	0	0	0	0	0	0	2-11	< 0.1	18-27	< 0.1	0-2	< 0.1
Lebetimonas	0	0	0	0	0	0	0	0	1-5	< 0.1	3-14	< 0.1	0-1	< 0.1
Nautiliaceae un.	0	0	0	0	0	0	0-1	< 0.1	0-2	< 0.1	2-10	< 0.1	0	0
Total 16S RNA sequences	354038-356011		342402		342209-352516		331906-360370		345547-360887		349056-359510		348360-356154	

SWIR: SouthWest Indian Ridge
Seq. n: number of sequences
%: percentage of total number of Campylobacterota sequences

Supplementary Table 2. Statistics and quality report for the draft genomes of ^{U}*Sulfurimonas pluma* assembled from Gakkel Ridge's hydrothermal plumes. For comparison the same information is reported for the closest isolated species *Sulfurimonas autotrophica*.

	^{U}*S. pluma* MAG-1	^{U}*S. pluma* MAG-2	*S. autotrophica* DSM 16294
Assembly accession	CAOKNM000000000	CAOKTG000000000	GCA 000147355
No. contigs	39	25	1
Total length	1,773,918	1,689,359	2,153,198
GC content	34	34	35
Completeness			
CheckM (v.1.2.1)	98.28	98.28	100
CheckM2 (v.0.1.3)	99.93	99.95	99.99
BUSCO (v.5.2.2)	94.6	93.6	94.7
Redundancy			
CheckM (v.1.2.1)	1.72	1.72	1.72
CheckM2 (v.0.1.3)	2.74	1.31	0.07
BUSCO (v.5.2.2)	2.2	0.3	1.1
Strain Heterogeneity	0	0	0
N50	113080	127483	-
No. genes	1949	1894	2,227
No. rrna	7[a]	7[a]	12
No. trna (No. type)	44 (19)	43 (18)	43
Coding density	1.12	1.11	1.04

[a] 5S rRNA in 5 copies

Supplementary Table 3. Compilation of data from environments hosting large proportion of 16S rRNA gene sequences of *Sulfurimonas*. The data reported refer to samples of sediments and their porewaters or water for aquifer and hard substrate (benthic), or to samples of seawater (pelagic). The concentrations of chemical species refer to dissolved compounds in porewater and water if not stated otherwise.

Environments	Geographic region	*Sulfurimonas* %	Temperature °C	Free hydrogen sulfide (H_2S) µmol L^{-1}	H_2 µmol L^{-1}	O_2 µmol L^{-1}	NO^{3-} µmol L^{-1}	Fe µmol L^{-1}	Reference
Benthic									
Coastal brackish benthic redox cline	Baltic Sea	up to 84	6.5	na	na	25	3	1−9[a]	[1]
Coastal marine benthic redox cline	North Sea	0.1−2	9.6	0−100	na	10−100	0.5−1.3[b]	na	[2]
Subsurface fresh water	Sweden	up to 83	12−15	0.53−1.29	na	2.3−7.2	185−223	23.5−27.8	[3]
Hydrothermal vent microbial mat	Mariana Arc	9−13	18.7−30.5	<0.4−816	<0.01−0.02	na	na	<0.1−96.7	[4]
Hydrothermal vent rock/chimney	Manus Basin	<1−37	na	na	na	na	na	na	[5]
Hydrothermal vent water/fluid	Manus Basin	5−24	3.6−98.6	0−3761	0.02−0.8	0−310	na	na	[5]
	Axial Seamount	12−16	24.5−27.9	452	<1−1.4	<30[c]	7.2	na	[6]
Pelagic									
Marine pelagic redox cline	Baltic Sea	20[d]	5−6	1.3−30	na	0.6−41.4	1	na	[7]
Rainbow non-buoyant hydrothermal plume	Mid Atlantic Ridge	21−58	<4	na	na	255−275	18−22	0.04−0.36[e]	[8]
Irina plume (Logatchev field)	Mid Atlantic Ridge	~70	<4	bdl	0.014	226	na	na	[9]
Aurora non-buoyant hydrothermal plume	Gakkel Ridge	15−64	−0.89	na	Up to 0.300[f]	307	15.6	up to 0.150[e]	this study, [10,11]
Polaris non-buoyant hydrothermal plume	Gakkel Ridge	4−17	−0.74	nag	up to 0.360	303	14.8	bdl	this study, [12]

[a] total iron;
[b] pers. comm. / unpublished data, Jana Friedrich, Helmholtz-Zentrum hereon
[c] estimated from percent seawater;
[d] based on cell count;
[e] solid iron (Fe^{3+});
[f] calculated minimum concentration from thermodynamic equilibrium (based on the assumption of $\Delta G=0$ at 370°C for the H_2/CO_2-CH_4 couple) and dilution of non-buoyant hydrothermal plume;
[g] some samples had sulfidic smell;
na: not available; bdl: below detection limit.

Supplementary Table 4. Metadata and sequence statistics of the samples used in the analysis of *Sulfurimonas* oligotypes.

ENA study accession	ENA run accession	Longitude	Latitude	Environment category	Total number of sequences	Number of *Sulfurimonas* sequences	Percentage of *Sulfurimonas* sequences	Number of *Sulfurimonas* oligotypes
PRJNA322450	SRR3584436	16.58553428	57.89297088	brackish.benthic.coastal	65665	46674	71.08	109
PRJNA322450	SRR3584437	16.58553428	57.89297088	brackish.benthic.coastal	68748	55590	80.86	183
PRJNA322450	SRR3584447	16.58553428	57.89297088	brackish.benthic.coastal	44670	2781	6.23	22
PRJNA322450	SRR3584448	16.58553428	57.89297088	brackish.benthic.coastal	70210	2118	3.02	20
PRJNA322450	SRR3584501	16.58553428	57.89297088	brackish.benthic.coastal	57667	2092	3.63	32
PRJNA322450	SRR3584508	16.58553428	57.89297088	brackish.benthic.coastal	83276	61800	74.21	140
PRJNA322450	SRR3584509	16.58553428	57.89297088	brackish.benthic.coastal	75207	62854	83.57	176
PRJNA322450	SRR3584512	16.58553428	57.89297088	brackish.benthic.coastal	56896	65	0.11	28
PRJNA322450	SRR3584514	16.58553428	57.89297088	brackish.benthic.coastal	59471	70	0.12	27
PRJNA322450	SRR3584397	16.58553428	57.89297088	brackish.benthic.coastal	51516	90	0.17	39
PRJNA322450	SRR3584406	16.58553428	57.89297088	brackish.benthic.coastal	40780	2839	6.96	125
PRJNA322450	SRR3584408	16.58553428	57.89297088	brackish.benthic.coastal	27872	2000	7.18	152
PRJEB11384	ERR1056320	150.817953	-9.824311	HV-substrate.benthic.coastal.marine	57046	59	0.10	13
PRJEB11384	ERR1056334	150.817431	-9.823519	HV-substrate.benthic.coastal.marine	117541	191	0.16	16
PRJEB11384	ERR1056318	150.817836	-9.824219	HV-substrate.benthic.coastal.marine	247169	811	0.33	12
PRJNA282077	SRR1994695	-7.8254	36.9988	benthic.coastal.marine	4100	11	0.27	8
PRJNA282077	SRR1994769	-7.8254	36.9988	benthic.coastal.marine	6497	23	0.35	8
PRJNA282077	SRR1994770	-7.8254	36.9988	benthic.coastal.marine	8795	51	0.58	10
PRJNA282077	SRR1994771	-7.8254	36.9988	benthic.coastal.marine	9141	33	0.36	9
PRJNA282077	SRR1994772	-7.8254	36.9988	benthic.coastal.marine	14143	61	0.43	16
PRJNA282077	SRR1994773	-7.8254	36.9988	benthic.coastal.marine	13034	36	0.28	11
PRJNA282077	SRR1994774	-7.8254	36.9988	benthic.coastal.marine	6812	54	0.79	11
PRJNA282077	SRR1994759	-7.8254	36.9988	benthic.coastal.marine	7553	31	0.41	11
PRJNA282077	SRR2000144	-7.8254	36.9988	benthic.coastal.marine	10719	18	0.17	7
PRJNA282077	SRR2000194	-3.9766	48.7269	benthic.coastal.marine	8927	13	0.15	9
PRJNA282077	SRR2000197	-3.9766	48.7269	benthic.coastal.marine	12383	17	0.14	9
PRJNA282077	SRR2000198	-3.9766	48.7269	benthic.coastal.marine	8482	16	0.19	11
PRJNA282077	SRR1994760	-7.8254	36.9988	benthic.coastal.marine	10529	42	0.40	12
PRJNA282077	SRR2000199	-3.9766	48.7269	benthic.coastal.marine	9083	17	0.19	8
PRJNA282077	SRR2000200	-3.9569	48.6909	benthic.coastal.marine	20740	43	0.21	12
PRJNA282077	SRR2000201	-3.9569	48.6909	benthic.coastal.marine	10667	28	0.26	9
PRJNA282077	SRR2000202	-3.9569	48.6909	benthic.coastal.marine	7021	25	0.36	11
PRJNA282077	SRR2000203	-3.9569	48.6909	benthic.coastal.marine	13046	63	0.48	13
PRJNA282077	SRR2000204	-3.9569	48.6909	benthic.coastal.marine	8959	65	0.73	13
PRJNA282077	SRR2000212	-3.9766	48.7269	benthic.coastal.marine	9714	10	0.10	7
PRJNA282077	SRR2000215	-3.9569	48.6909	benthic.coastal.marine	14439	112	0.78	12
PRJNA282077	SRR2000217	-3.9569	48.6909	benthic.coastal.marine	16657	72	0.43	14
PRJNA282077	SRR2000235	-3.9569	48.6909	benthic.coastal.marine	18894	51	0.27	12
PRJNA282077	SRR2000237	-3.9569	48.6909	benthic.coastal.marine	17694	49	0.28	10
PRJNA282077	SRR2000238	-3.9569	48.6909	benthic.coastal.marine	15485	30	0.19	8
PRJNA282077	SRR1994763	-7.8254	36.9988	benthic.coastal.marine	6660	23	0.35	10
PRJNA282077	SRR1994764	-7.8254	36.9988	benthic.coastal.marine	12088	18	0.15	7
PRJNA282077	SRR1994765	-7.8254	36.9988	benthic.coastal.marine	11529	41	0.36	11
PRJNA282077	SRR1994767	-7.8254	36.9988	benthic.coastal.marine	8066	12	0.15	8
PRJNA282077	SRR1994768	-7.8254	36.9988	benthic.coastal.marine	12500	88	0.70	12
PRJEB18774	ERR1779777	5.578333	54.825	benthic.coastal.marine	101150	492	0.49	47
PRJEB18774	ERR1779778	5.578333	54.825	benthic.coastal.marine	77627	830	1.07	55
PRJEB18774	ERR1779779	5.578333	54.825	benthic.coastal.marine	16972	57	0.34	20
PRJEB18774	ERR1779780	5.578333	54.825	benthic.coastal.marine	10723	15	0.14	10
PRJEB18774	ERR1779761	4.7495	55.2585	benthic.coastal.marine	14768	20	0.14	7
PRJEB15554	ERR1665221	152.1331667	-3.858	HV-plume.pelagic.deep-sea.marine	105186	3176	3.02	405
PRJEB15554	ERR1665230	151.672122	-3.728875	HV-substrate.benthic.deep-sea.marine	328602	11637	3.54	515
PRJEB15554	ERR1665232	151.672519	-3.728279	HV-fluid.benthic.deep-sea.marine	18564	929	5.00	215
PRJEB15554	ERR1665233	151.6724167	-3.728333333	HV-fluid.benthic.deep-sea.marine	19925	901	4.52	213
PRJEB15554	ERR1665234	151.672394	-3.728333	HV-fluid.benthic.deep-sea.marine	14783	628	4.25	185
PRJEB15554	ERR1665235	151.6723802	-3.728289167	HV-fluid.benthic.deep-sea.marine	25114	1450	5.77	280
PRJEB15554	ERR1665236	151.672386	-3.728303	HV-fluid.benthic.deep-sea.marine	15695	1087	6.93	239
PRJEB15554	ERR1665237	151.672495	-3.728297833	HV-fluid.benthic.deep-sea.marine	13784	925	6.71	221
PRJEB15554	ERR1665238	151.672181	-3.728856	HV-fluid.benthic.deep-sea.marine	45370	1824	4.02	295
PRJEB15554	ERR1665239	151.672165	-3.728828	HV-fluid.benthic.deep-sea.marine	13474	1173	8.71	214
PRJEB15554	ERR1665222	152.1333333	-3.8585	HV-plume.pelagic.deep-sea.marine	447112	5278	1.18	448
PRJEB15554	ERR1665240	151.672157	-3.728789	HV-fluid.benthic.deep-sea.marine	19237	2525	13.13	296
PRJEB15554	ERR1665241	151.672427	-3.7283	HV-fluid.benthic.deep-sea.marine	14465	768	5.31	169
PRJEB15554	ERR1665242	151.672619	-3.728267	HV-fluid.benthic.deep-sea.marine	4989	180	3.61	75
PRJEB15554	ERR1665243	151.672205	-3.728794	HV-fluid.benthic.deep-sea.marine	17664	1210	6.85	211
PRJEB15554	ERR1665244	152.101826	-3.8014	HV-fluid.benthic.deep-sea.marine	37215	2087	5.61	370
PRJEB15554	ERR1665245	152.100281	-3.800638	HV-fluid.benthic.deep-sea.marine	131251	31782	24.21	386
PRJEB15554	ERR1665246	152.1008583	-3.799910667	HV-fluid.benthic.deep-sea.marine	136078	6509	4.78	295
PRJEB15554	ERR1665247	152.1008595	-3.799916333	HV-fluid.benthic.deep-sea.marine	41632	2297	5.52	297
PRJEB15554	ERR1665248	152.1008743	-3.7999335	HV-fluid.benthic.deep-sea.marine	83582	1416	1.69	202
PRJEB15554	ERR1665249	152.1013353	-3.799260833	HV-fluid.benthic.deep-sea.marine	26494	5114	19.30	371
PRJEB15554	ERR1665250	152.1014963	-3.800707167	HV-fluid.benthic.deep-sea.marine	28974	2663	9.19	300
PRJEB15554	ERR1665251	152.101383	-3.799288	HV-fluid.benthic.deep-sea.marine	83239	10644	12.79	409
PRJEB15554	ERR1665252	152.10094	-3.79996	HV-fluid.benthic.deep-sea.marine	14093	1119	7.94	226
PRJEB15554	ERR1665253	152.100477	-3.799855	HV-fluid.benthic.deep-sea.marine	38621	5707	14.78	323
PRJEB15554	ERR1665254	152.1007167	-3.7994	HV-substrate.benthic.deep-sea.marine	51501	7843	15.23	512
PRJEB15554	ERR1665255	152.1007167	-3.7994	HV-substrate.benthic.deep-sea.marine	134770	3746	2.78	416
PRJEB15554	ERR1665256	152.1007167	-3.7994	HV-substrate.benthic.deep-sea.marine	49714	10766	21.66	473
PRJEB15554	ERR1665257	152.1007167	-3.7994	HV-substrate.benthic.deep-sea.marine	39622	7500	18.93	473
PRJEB15554	ERR1665262	151.675333	-3.720648	HV-substrate.benthic.deep-sea.marine	542017	10562	1.95	472
PRJEB15554	ERR1665263	151.675333	-3.720648	HV-substrate.benthic.deep-sea.marine	70772	6051	8.55	514
PRJEB15554	ERR1665265	151.675313	-3.720639	HV-fluid.benthic.deep-sea.marine	53767	2710	5.04	357
PRJEB15554	ERR1665266	151.674553	-3.721194	HV-substrate.benthic.deep-sea.marine	322740	29050	9.00	514
PRJEB15554	ERR1665267	151.679793	-3.728119	HV-substrate.benthic.deep-sea.marine	216666	81253	37.50	563
PRJEB15554	ERR1665269	151.672749	-3.717165	HV-fluid.benthic.deep-sea.marine	22262	1060	4.76	250
PRJEB15554	ERR1665271	151.674185	-3.730529	HV-substrate.benthic.deep-sea.marine	523205	4223	0.81	463
PRJEB15554	ERR1665272	151.6720222	-3.7268755	HV-fluid.benthic.deep-sea.marine	8123	975	12.00	220
PRJEB15554	ERR1665273	151.6719672	-3.726450667	HV-fluid.benthic.deep-sea.marine	7800	1604	20.56	265
PRJEB15554	ERR1665274	151.672067	-3.726929	HV-fluid.benthic.deep-sea.marine	17327	2726	15.73	337
PRJEB15554	ERR1665275	151.672078	-3.726941	HV-fluid.benthic.deep-sea.marine	3723	516	13.86	165
PRJEB15554	ERR1665276	151.672144	-3.726833	HV-substrate.benthic.deep-sea.marine	278380	6010	2.16	507
PRJEB15554	ERR1665226	151.6693127	-3.728074167	HV-substrate.benthic.deep-sea.marine	61542	6294	10.23	450
PRJEB15554	ERR1665227	151.6693127	-3.728074167	HV-substrate.benthic.deep-sea.marine	70931	10754	15.16	486
PRJEB15554	ERR1665228	151.6720235	-3.7268905	HV-substrate.benthic.deep-sea.marine	54776	2790	5.09	398
PRJNA379939	SRR5363780	-61.362047	15.232771	HV-substrate.benthic.coastal.marine	93512	470	0.50	25
PRJNA379939	SRR5363779	-61.362047	15.232771	HV-substrate.benthic.coastal.marine	38872	40	0.10	9
PRJEB32776	ERR3341283	-93.24608333	22.02201667	benthic.deep-sea.marine	69957	5406	7.73	129
PRJEB32776	ERR3341288	-93.24608333	22.02201667	benthic.deep-sea.marine	166236	1043	0.63	50
PRJEB32776	ERR7440584	-93.4371	21.8994	benthic.deep-sea.marine	67208	1485	2.21	32
PRJEB32776	ERR7440582	-93.24608333	22.02201667	benthic.deep-sea.marine	71909	1067	1.48	19
PRJEB32776	ERR3341282	-93.24608333	22.02201667	benthic.deep-sea.marine	57719	2483	4.30	68
PRJEB32776	ERR7440580	-93.24608333	22.02201667	benthic.deep-sea.marine	112959	2659	2.35	23
PRJEB32776	ERR7440579	-93.24608333	22.02201667	benthic.deep-sea.marine	67132	129	0.19	24
PRJEB32776	ERR3341281	-93.24608333	22.02201667	benthic.deep-sea.marine	45353	1719	3.79	52
PRJEB32776	ERR3341280	-93.24608333	22.02201667	benthic.deep-sea.marine	30287	844	2.79	32
PRJEB32776	ERR3341293	-93.24608333	22.02201667	benthic.deep-sea.marine	34813	496	1.42	61
PRJEB32776	ERR3341292	-93.24608333	22.02201667	benthic.deep-sea.marine	40283	306	0.76	29
PRJEB32776	ERR3341291	-93.24608333	22.02201667	benthic.deep-sea.marine	49933	353	0.71	26
PRJEB32776	ERR3341290	-93.24608333	22.02201667	benthic.deep-sea.marine	26420	319	1.21	36
PRJEB32776	ERR3341289	-93.24608333	22.02201667	benthic.deep-sea.marine	51237	690	1.35	52
PRJEB10576	ERR2188824	16.65996	57.43319	subsurface.fresh water	31589	1475	4.67	15
PRJEB10576	ERR2188824	16.65996	57.43319	subsurface.fresh water	32639	1357	4.16	10
PRJEB10576	ERR2188824	16.65996	57.43319	subsurface.fresh water	58716	43276	73.70	19
PRJEB10576	ERR2188824	16.65996	57.43319	subsurface.fresh water	81386	19621	24.11	42
PRJEB10576	ERR2188824	16.65996	57.43319	subsurface.fresh water	22074	358	1.62	5
PRJEB10576	ERR2188824	16.65996	57.43319	subsurface.fresh water	21931	5306	24.19	38
PRJEB10576	ERR2188824	16.65996	57.43319	subsurface.fresh water	29442	1208	4.10	10
PRJEB10576	ERR2188824	16.65996	57.43319	subsurface.fresh water	17139	5488	32.02	30
PRJEB10576	ERR2188824	16.65996	57.43319	subsurface.fresh water	20805	1189	5.71	16
PRJEB10576	ERR2188824	16.65996	57.43319	subsurface.fresh water	27500	8871	32.26	38
PRJEB43086	ERR5293826	4.3295	79.01733	sediment trap.pelagic.deep-sea.marine	133671	2750	2.06	17
PRJEB43086	ERR5293805	4.3295	79.01733	sediment trap.pelagic.deep-sea.marine	57219	36615	63.99	36
PRJEB43086	ERR5293825	4.3295	79.01733	sediment trap.pelagic.deep-sea.marine	102137	2553	2.50	18
PRJEB23821	ERR2215447	13.0645	-52.4965	benthic.deep-sea.marine	46187	220	0.48	26
PRJEB48226	ERR7132489	55.93484	86.95334	HV-plume.pelagic.deep-sea.marine	55976	1883	3.36	42
PRJEB48226	ERR7132488	55.91866	86.95216	HV-plume.pelagic.deep-sea.marine	52947	2263	4.27	47
PRJEB48226	ERR7132487	55.94066	86.95396	HV-plume.pelagic.deep-sea.marine	45776	1905	4.16	49
PRJEB48226	ERR7132486	55.95684	86.95534	HV-plume.pelagic.deep-sea.marine	53614	2579	4.81	53
PRJEB48226	ERR7132483	55.67484	86.9585	HV-plume.pelagic.deep-sea.marine	14009	861	6.15	28
PRJEB48226	ERR7132482	55.679	86.95516	HV-plume.pelagic.deep-sea.marine	53114	2316	4.36	40
PRJEB48226	ERR7132553	55.83284	86.9605	HV-plume.pelagic.deep-sea.marine	66118	8578	12.97	57
PRJEB48226	ERR7132512	-6.246833	82.896	HV-plume.pelagic.deep-sea.marine	60698	29344	48.34	58
PRJEB48226	ERR7132510	-6.091	82.9085	HV-plume.pelagic.deep-sea.marine	45624	13527	29.65	55
PRJEB48226	ERR7132508	-6.254333	82.901333	HV-plume.pelagic.deep-sea.marine	50996	31152	61.09	59
PRJEB48226	ERR7132509	-6.254333	82.901333	HV-plume.pelagic.deep-sea.marine	52961	6859	12.95	54
PRJEB48226	ERR7132504	-6.231833	82.896667	HV-plume.pelagic.deep-sea.marine	43797	5863	13.39	54
PRJEB48226	ERR7132506	-6.231833	82.896667	HV-plume.pelagic.deep-sea.marine	54851	1041	1.90	37
PRJEB48226	ERR7132502	-6.279167	82.8965	HV-plume.pelagic.deep-sea.marine	72899	14057	19.28	55
PRJEB48226	ERR7132501	-6.279167	82.8965	HV-plume.pelagic.deep-sea.marine	51966	7353	14.15	55
PRJEB48226	ERR7132499	-6.268833	82.899167	HV-plume.pelagic.deep-sea.marine	77951	10662	13.68	58
PRJEB48226	ERR7132497	-2.466333	83.105167	HV-plume.pelagic.deep-sea.marine	57051	1024	1.79	35
PRJEB48226	ERR7132511	-6.246833	82.896	HV-plume.pelagic.deep-sea.marine	71753	9016	12.57	56
PRJEB48226	ERR7132507	-6.254333	82.901333	HV-plume.pelagic.deep-sea.marine	66100	7038	10.65	56
PRJEB48226	ERR7132505	-6.231833	82.896667	HV-plume.pelagic.deep-sea.marine	37896	6862	18.11	56
PRJEB48226	ERR7132503	-6.279167	82.8965	HV-plume.pelagic.deep-sea.marine	79479	8299	10.44	55
PRJEB48226	ERR7132500	-6.268833	82.899167	HV-plume.pelagic.deep-sea.marine	87864	14134	16.09	57

ENA study accession	ENA run accession	Longitude	Latitude	Environment category	Total number of sequences	Number of *Sulfurimonas* sequences	Percentage of *Sulfurimonas* sequences	Number of *Sulfurimonas* oligotypes
PRJEB23972	ERR2222747	13.278833	-52.4375	HV-plume.pelagic.deep-sea.marine	119466	3860	3.23	39
PRJEB23972	ERR2222745	13.278833	-52.4375	HV-plume.pelagic.deep-sea.marine	41516	2065	4.97	26
PRJEB23972	ERR2222746	13.278833	-52.4375	HV-plume.pelagic.deep-sea.marine	97351	4189	4.30	37
PRJEB23972	ERR2222749	12.526167	-52.6455	HV-plume.pelagic.deep-sea.marine	127628	4259	3.34	33
PRJEB23972	ERR2222750	15.738167	-52.232667	HV-plume.pelagic.deep-sea.marine	129495	10324	7.97	54
PRJEB23972	ERR2222752	15.738167	-52.232667	HV-plume.pelagic.deep-sea.marine	68613	3503	5.11	39
PRJEB23972	ERR2222751	15.738167	-52.232667	HV-plume.pelagic.deep-sea.marine	114905	5724	4.98	49
PRJEB33205	ERR3397401	-88.45003	-7.1263	plastic.benthic.deep-sea.marine	57307	8098	14.13	5
PRJEB33205	ERR3397399	-88.46875	-7.0835	plastic.benthic.deep-sea.marine	59858	6957	11.62	8
PRJEB48226	ERR7132491	55.57533	86.951	HV-plume.pelagic.deep-sea.marine	75207	3209	4.27	55
PRJEB48226	ERR7132492	55.57533	86.951	HV-plume.pelagic.deep-sea.marine	52946	594	1.12	34
PRJNA318932	SRR3416727	122.58	25.07	HV-substrate.benthic.deep-sea.marine	64631	103	0.16	27
PRJNA318932	SRR3416728	122.58	25.07	HV-substrate.benthic.deep-sea.marine	72335	846	1.17	97
PRJNA318932	SRR3417104	122.58	25.07	HV-substrate.benthic.deep-sea.marine	103161	1018	0.99	80
PRJNA318932	SRR3417109	122.7	24.85	benthic.deep-sea.marine	104134	153	0.15	35
PRJNA318932	SRR3417110	122.7	24.85	benthic.deep-sea.marine	87176	102	0.12	31
PRJNA352433	SRR5004721	144.042	21.488	HV-substrate.benthic.deep-sea.marine	509074	6369	1.25	161
PRJNA352433	SRR5004730	143.649	12.922	HV-substrate.benthic.deep-sea.marine	314345	16528	5.26	250
PRJNA352433	SRR5004731	143.649	12.922	HV-substrate.benthic.deep-sea.marine	195980	282	0.14	42
PRJNA352433	SRR5004732	143.649	12.922	HV-substrate.benthic.deep-sea.marine	65848	3449	5.24	119
PRJNA352433	SRR5004733	144.042	21.488	HV-substrate.benthic.deep-sea.marine	529278	9283	1.75	219
PRJNA352433	SRR5004734	143.649	12.922	HV-substrate.benthic.deep-sea.marine	78877	4233	5.37	100
PRJNA352433	SRR5004736	143.649	12.922	HV-substrate.benthic.deep-sea.marine	943675	102920	10.91	400
PRJNA352433	SRR5004737	144.778	14.601	HV-substrate.benthic.deep-sea.marine	565536	14113	2.50	250
PRJNA352433	SRR5004738	144.776	14.601	HV-substrate.benthic.deep-sea.marine	505576	63202	12.50	316
PRJNA352433	SRR5004739	143.619	12.952	HV-substrate.benthic.deep-sea.marine	298950	456	0.15	53
PRJNA352433	SRR5004740	143.649	12.922	HV-substrate.benthic.deep-sea.marine	541285	30857	5.70	250
PRJNA352433	SRR5004722	143.619	12.952	HV-substrate.benthic.deep-sea.marine	506807	885	0.17	65
PRJNA352433	SRR5004741	144.042	21.488	HV-substrate.benthic.deep-sea.marine	552945	49708	8.99	195
PRJNA352433	SRR5004742	144.042	21.488	HV-substrate.benthic.deep-sea.marine	145712	1688	1.16	116
PRJNA352433	SRR5004723	143.649	12.922	HV-substrate.benthic.deep-sea.marine	177283	950	0.54	80
PRJNA352433	SRR5004724	144.042	21.488	HV-substrate.benthic.deep-sea.marine	421412	15386	3.65	170
PRJNA352433	SRR5004725	143.649	12.922	HV-substrate.benthic.deep-sea.marine	147492	2840	1.93	84
PRJNA352433	SRR5004726	144.041	21.487	HV-substrate.benthic.deep-sea.marine	351336	5035	1.43	110
PRJNA352433	SRR5004727	144.042	21.488	HV-substrate.benthic.deep-sea.marine	1619588	52837	3.26	281
PRJNA352433	SRR5004728	144.778	14.601	HV-substrate.benthic.deep-sea.marine	527521	32739	6.21	298
PRJNA352433	SRR5004729	144.042	21.488	HV-substrate.benthic.deep-sea.marine	617347	12610	2.04	178
PRJNA360358	SRR5150126	2.93	51.23	plastic.benthic.coastal.marine	175032	571	0.33	60
PRJNA360358	SRR5150240	2.93	51.23	plastic.benthic.coastal.marine	86481	671	0.78	70
PRJNA360358	SRR5150243	2.93	51.23	plastic.benthic.coastal.marine	128963	223	0.17	49
PRJNA360358	SRR5150244	2.93	51.23	plastic.benthic.coastal.marine	26232	123	0.47	32
PRJNA360358	SRR5150245	2.93	51.23	plastic.benthic.coastal.marine	53132	57	0.11	27
PRJNA360358	SRR5150246	2.93	51.23	plastic.benthic.coastal.marine	61576	451	0.73	53
PRJNA360358	SRR5150247	2.93	51.23	plastic.benthic.coastal.marine	67475	338	0.50	53
PRJNA360358	SRR5150249	2.99	51.85	plastic.benthic.coastal.marine	36304	42	0.12	14
PRJNA360358	SRR5150252	2.93	51.23	plastic.benthic.coastal.marine	33200	145	0.44	41
PRJNA360358	SRR5150253	2.93	51.23	plastic.benthic.coastal.marine	44106	154	0.35	35
PRJNA360358	SRR5150258	2.93	51.23	plastic.benthic.coastal.marine	28462	52	0.18	13
PRJNA360358	SRR5150137	2.93	51.23	plastic.benthic.coastal.marine	86024	546	0.63	63
PRJNA360358	SRR5150260	2.93	51.23	plastic.benthic.coastal.marine	68736	186	0.27	29
PRJNA360358	SRR5150262	2.99	51.85	plastic.benthic.coastal.marine	47651	108	0.23	21
PRJNA360358	SRR5150266	2.93	51.23	plastic.benthic.coastal.marine	104760	146	0.14	42
PRJNA360358	SRR5150268	2.93	51.23	plastic.benthic.coastal.marine	32132	33	0.10	22
PRJNA360358	SRR5150138	2.93	51.23	plastic.benthic.coastal.marine	51201	180	0.35	36
PRJNA360358	SRR5150271	2.93	51.23	plastic.benthic.coastal.marine	68908	248	0.36	56
PRJNA360358	SRR5150273	2.93	51.23	plastic.benthic.coastal.marine	53972	76	0.14	31
PRJNA360358	SRR5150139	2.99	51.85	plastic.benthic.coastal.marine	94063	276	0.29	31
PRJNA360358	SRR5150140	2.99	51.85	plastic.benthic.coastal.marine	88565	174	0.20	29
PRJNA360358	SRR5150141	2.93	51.23	plastic.benthic.coastal.marine	63873	77	0.12	38
PRJNA360358	SRR5150142	2.99	51.85	plastic.benthic.coastal.marine	28447	269	0.95	32
PRJNA360358	SRR5150143	2.93	51.23	plastic.benthic.coastal.marine	31597	100	0.32	31
PRJNA360358	SRR5150127	2.93	51.23	plastic.benthic.coastal.marine	12398	427	3.44	44
PRJNA360358	SRR5150146	2.93	51.23	plastic.benthic.coastal.marine	24651	904	3.67	81
PRJNA360358	SRR5150150	2.93	51.23	plastic.benthic.coastal.marine	36309	140	0.39	30
PRJNA360358	SRR5150151	2.99	51.85	plastic.benthic.coastal.marine	10004	42	0.42	12
PRJNA360358	SRR5150154	2.93	51.23	plastic.benthic.coastal.marine	73343	202	0.28	58
PRJNA360358	SRR5150128	2.99	51.85	plastic.benthic.coastal.marine	91305	971	1.06	54
PRJNA360358	SRR5150155	2.93	51.23	plastic.benthic.coastal.marine	1364	4	0.29	3
PRJNA360358	SRR5150157	2.93	51.23	plastic.benthic.coastal.marine	234306	1348	0.58	78
PRJNA360358	SRR5150158	2.93	51.23	plastic.benthic.coastal.marine	72963	390	0.53	55
PRJNA360358	SRR5150160	2.93	51.23	plastic.benthic.coastal.marine	71536	79	0.11	31
PRJNA360358	SRR5150161	2.99	51.85	plastic.benthic.coastal.marine	61121	96	0.16	18
PRJNA360358	SRR5150163	2.93	51.23	plastic.benthic.coastal.marine	47487	945	1.99	51
PRJNA360358	SRR5150168	2.93	51.23	plastic.benthic.coastal.marine	57610	104	0.18	34
PRJNA360358	SRR5150174	2.93	51.23	plastic.benthic.coastal.marine	60198	239	0.40	55
PRJNA360358	SRR5150130	2.93	51.23	plastic.benthic.coastal.marine	39863	914	2.29	51
PRJNA360358	SRR5150176	2.99	51.85	plastic.benthic.coastal.marine	17087	35	0.20	10
PRJNA360358	SRR5150177	2.93	51.23	plastic.benthic.coastal.marine	78172	2141	2.74	65
PRJNA360358	SRR5150179	2.93	51.23	plastic.benthic.coastal.marine	56475	158	0.28	42
PRJNA360358	SRR5150184	2.93	51.23	plastic.benthic.coastal.marine	62303	100	0.16	24
PRJNA360358	SRR5150185	2.93	51.23	plastic.benthic.coastal.marine	48419	2803	5.79	79
PRJNA360358	SRR5150186	2.93	51.23	plastic.benthic.coastal.marine	116267	330	0.28	51
PRJNA360358	SRR5150194	2.99	51.85	plastic.benthic.coastal.marine	139764	175	0.13	29
PRJNA360358	SRR5150200	2.93	51.23	plastic.benthic.coastal.marine	43525	58	0.13	25
PRJNA360358	SRR5150205	2.93	51.23	plastic.benthic.coastal.marine	127373	179	0.14	42
PRJNA360358	SRR5150207	2.93	51.23	plastic.benthic.coastal.marine	31478	107	0.34	31
PRJNA360358	SRR5150208	2.93	51.23	plastic.benthic.coastal.marine	68020	672	0.99	73
PRJNA360358	SRR5150216	2.93	51.23	plastic.benthic.coastal.marine	42348	314	0.74	47
PRJNA360358	SRR5150219	2.93	51.23	plastic.benthic.coastal.marine	79758	316	0.40	54
PRJNA360358	SRR5150220	2.93	51.23	plastic.benthic.coastal.marine	61521	517	0.84	61
PRJNA360358	SRR5150221	2.99	51.85	plastic.benthic.coastal.marine	88701	651	0.73	56
PRJNA360358	SRR5150222	2.93	51.23	plastic.benthic.coastal.marine	73740	661	0.90	64
PRJNA360358	SRR5150223	2.93	51.23	plastic.benthic.coastal.marine	20396	973	4.77	65
PRJNA360358	SRR5150227	2.93	51.23	plastic.benthic.coastal.marine	29248	104	0.36	26
PRJNA360358	SRR5150237	2.99	51.85	plastic.benthic.coastal.marine	117942	223	0.19	36
PRJNA386676	SRR5569842	-61.373123	15.245846	HV-substrate.benthic.coastal.marine	53841	191	0.35	33
PRJNA386676	SRR5569835	-61.373123	15.245846	benthic.coastal.marine	35417	58	0.16	22
PRJNA414441	SRR6179418	71	-26	HV-plume.pelagic.deep-sea.marine	806248	9053	1.12	195
PRJNA414441	SRR6179419	70.04	-25.31	HV-substrate.benthic.deep-sea.marine	530387	36005	6.79	272
PRJNA414441	SRR6179421	70.04	-25.31	HV-plume.pelagic.deep-sea.marine	546738	5568	1.02	60
PRJNA414441	SRR6179422	71	-26	HV-plume.pelagic.deep-sea.marine	603466	8556	1.42	76
PRJNA414441	SRR6179410	70.04	-25.31	HV-substrate.benthic.deep-sea.marine	845082	5845	0.69	148
PRJNA414441	SRR6179411	71	-26	HV-substrate.benthic.deep-sea.marine	825326	10490	1.27	128
PRJNA414441	SRR6179412	71	-26	HV-substrate.benthic.deep-sea.marine	580694	93931	16.18	409
PRJNA414441	SRR6179413	71	-26	HV-substrate.benthic.deep-sea.marine	562476	345126	61.36	382
PRJNA414441	SRR6179414	70.04	-25.31	HV-substrate.benthic.deep-sea.marine	544450	8384	1.54	17
PRJNA414441	SRR6179415	70.04	-25.31	HV-fluid.benthic.deep-sea.marine	711728	33489	4.71	449
PRJNA414441	SRR6179417	71	-26	HV-fluid.benthic.deep-sea.marine	899677	58843	6.54	582
PRJNA485064	SRR7659209	-6.97	35.44	HV-substrate.benthic.deep-sea.marine	35834	293	0.82	15
PRJNA485064	SRR7659211	-6.97	35.44	HV-substrate.benthic.deep-sea.marine	38862	301	0.77	19
PRJNA485064	SRR7659212	-6.97	35.44	HV-substrate.benthic.deep-sea.marine	32218	248	0.77	17
PRJNA485064	SRR7659214	-6.97	35.44	HV-substrate.benthic.deep-sea.marine	37060	99	0.27	14
PRJNA511010	SRR8361279	-86.17	27.47	benthic.deep-sea.marine	87195	808	0.93	44
PRJNA511010	SRR8361280	-86.24	27.52	benthic.deep-sea.marine	109795	136	0.12	3
PRJNA511010	SRR8361281	-86.01	27.43	benthic.deep-sea.marine	163159	658	0.40	45
PRJNA511010	SRR8361306	-86.62	27.8	benthic.deep-sea.marine	29484	51	0.17	5
PRJNA511010	SRR8361322	-89.7	28.23	benthic.deep-sea.marine	37245	189	0.51	19
PRJNA511010	SRR8361180	-86.11	27.47	benthic.deep-sea.marine	165108	173	0.10	5
PRJNA511010	SRR8361209	-86.54	27.72	benthic.deep-sea.marine	106346	222	0.21	9
PRJNA511010	SRR8361211	-85.9	27.27	benthic.deep-sea.marine	25768	27	0.10	8
PRJNA524261	SRR8639477	122.6379	37.161266	benthic.coastal.marine	33680	79	0.23	12
PRJNA524261	SRR8639487	122.60099	37.182907	benthic.coastal.marine	30134	322	1.07	43
PRJNA524261	SRR8639488	122.60099	37.182907	benthic.coastal.marine	19770	186	0.94	38
PRJNA524261	SRR8639491	122.60552	37.185394	benthic.coastal.marine	26708	30	0.11	4
PRJNA524261	SRR8639492	122.60552	37.185394	benthic.coastal.marine	34044	48	0.14	3
PRJNA524261	SRR8639493	122.60099	37.182907	benthic.coastal.marine	33525	65	0.19	23
PRJNA524261	SRR8639494	122.60099	37.182907	benthic.coastal.marine	27414	118	0.43	25
PRJNA524261	SRR8639495	122.618126	37.1785	benthic.coastal.marine	24290	70	0.29	27
PRJNA524261	SRR8639478	122.618126	37.1785	benthic.coastal.marine	31791	186	0.59	11
PRJNA524261	SRR8639496	122.618126	37.1785	benthic.coastal.marine	20321	79	0.39	28
PRJNA524261	SRR8639497	122.618126	37.1785	benthic.coastal.marine	17891	40	0.22	17
PRJNA524261	SRR8639498	122.618126	37.1785	benthic.coastal.marine	20049	99	0.49	29
PRJNA524261	SRR8639500	122.618126	37.1785	benthic.coastal.marine	23824	52	0.22	4
PRJNA524261	SRR8639501	122.60099	37.182907	benthic.coastal.marine	20998	45	0.21	5
PRJNA524261	SRR8639503	122.60099	37.182907	benthic.coastal.marine	30705	53	0.17	8
PRJNA524261	SRR8639479	122.6379	37.161266	benthic.coastal.marine	32796	83	0.25	20
PRJNA524261	SRR8639507	122.60552	37.185394	benthic.coastal.marine	32995	57	0.17	10
PRJNA524261	SRR8639508	122.60552	37.185394	benthic.coastal.marine	28040	58	0.21	8
PRJNA524261	SRR8639509	122.60552	37.185394	benthic.coastal.marine	28862	48	0.17	10
PRJNA524261	SRR8639510	122.60552	37.185394	benthic.coastal.marine	16910	41	0.24	14
PRJNA524261	SRR8639480	122.6379	37.161266	benthic.coastal.marine	24775	158	0.64	30
PRJNA524261	SRR8639481	122.618126	37.1785	benthic.coastal.marine	22169	33	0.15	15
PRJNA524261	SRR8639483	122.618126	37.1785	benthic.coastal.marine	31599	83	0.26	23
PRJNA524261	SRR8639485	122.60099	37.182907	benthic.coastal.marine	30355	205	0.68	10
PRJNA549457	SRR9320285	-155.2	18.6	HV-substrate.benthic.deep-sea.marine	391907	501	0.13	18
PRJNA549457	SRR9320286	-155.2	18.6	HV-substrate.benthic.deep-sea.marine	552627	6780	1.23	96
PRJNA549457	SRR9320288	-155.2	18.6	HV-substrate.benthic.deep-sea.marine	236252	730	0.31	30
PRJNA549457	SRR9320277	-155.2	18.6	HV-substrate.benthic.deep-sea.marine	875676	52487	5.99	69
PRJNA549457	SRR9320281	-155.2	18.6	HV-substrate.benthic.deep-sea.marine	413592	1747	0.42	54
PRJNA549457	SRR9320282	-155.2	18.6	HV-substrate.benthic.deep-sea.marine	185288	2213	1.19	56
PRJNA563517	SRR10061065	49.6	-37.8	HV-substrate.benthic.deep-sea.marine	59265	110	0.19	15
PRJNA563517	SRR10061066	-102.5	-3.2	HV-substrate.benthic.deep-sea.marine	51342	650	1.27	14
PRJNA563517	SRR10061067	-14.5	-13.8	HV-substrate.benthic.deep-sea.marine	70937	238	0.34	41
PRJNA563517	SRR10061068	-14.4	-13.2	HV-plume.pelagic.deep-sea.marine	57721	1533	2.66	72
PRJNA563517	SRR10061069	-14.4	-13.2	HV-plume.pelagic.deep-sea.marine	40989	2122	5.18	102
PRJNA563517	SRR10061074	-14.4	-13.2	HV-plume.pelagic.deep-sea.marine	49016	8971	18.30	193
PRJNA299110	SRR2720402	126.97	27.55	HV-substrate.benthic.deep-sea.marine	179607	316	0.18	44
PRJNA299110	SRR2720405	126.97	27.55	HV-substrate.benthic.deep-sea.marine	126901	154	0.12	37
PRJNA330786	SRR3939031	151.6699	-3.728671	HV-substrate.benthic.deep-sea.marine	39488	75	0.19	36
PRJNA330786	SRR3939032	151.6774	-3.734023	HV-substrate.benthic.deep-sea.marine	42923	113	0.26	40
PRJNA330786	SRR3939033	151.8805	-3.712902	HV-substrate.benthic.deep-sea.marine	38120	154	0.40	41
PRJNA330786	SRR3939034	151.8639	-3.679219	HV-substrate.benthic.deep-sea.marine	43643	135	0.31	56

Supplementary Table 5. Comparison of marker genes for main metabolic pathways between known *Sulfurimonas* isolates and ^{U}*Sulfurimonas pluma*. The number of gene copies is given in parentheses.

Pathway	Enzime	Short name	*Sd*	*Sh*	*Sg*	*Sa*	*Sp1*
Hydrogen oxidation	[NiFe]-hydrogenase Group 1b, large subunit	HydB	+ (1)	+ (2)	+ (3)	+ (1)	+ (1)
	[NiFe]-hydrogenase Group 2d, large subunit	HupV / HyaB	+ (1)	+ (1)	+ (1)		
	[NiFe]-hydrogenase Group 4e, large subunit	EchE			+ (1)		
	[NiFe]-hydrogenase IV, large subunit	HycE				+ (1)	
Sulfide oxidation	flavocytochrome c sulfide dehydrogenase	FccB	+ (1)	+ (2)	+ (1)	-	+ (1)
		FccA	-	-	-	-	-
	sulfide:quinone reductase	Sqr	+ (3)	+ (5)	+ (5)	+ (5)	+ (1)
Sulfur oxidation	sulfur oxidation protein	SoxA*	+ (1)	+ (1)	+ (1)	+ (1)	+ (1)
		SoxB*	+ (1)	+ (1)	+ (1)	+ (1)	+ (1)
		SoxC*	+ (1)	+ (1)	+ (1)	+ (1)	+ (1)
		SoxD	+ (1)	+ (1)	+ (1)	+ (1)	+ (1)
		SoxY*	+ (2)	+ (2)	+ (2)	+ (2)	+ (2)
		SoxX*	+ (1)	+ (1)	+ (1)	+ (1)	+ (1)
		SoxZ	+ (2)	+ (2)	+ (2)	+ (2)	+ (2)
Sulfite oxidation	sulfite dehydrogenase	SorA	-	+ (1)	+ (1)	+ (1)	+ (1)
Assimilatory sulfate reduction	adenylylsulfate reductase (APSR)	CysD*	+ (1)	+ (1)	+ (2)	+ (1)	+ (1)
		CysN	+ (1)	+ (3)	+ (2)	+ (1)	+ (1)
	phosphoadenosine phosphosulfate reductase (PAPS)	CysH	+ (1)	-	+ (1)	-	+ (1)
	adenylylsulfate kinase	CysC	-	+ (4)	+ (2)	+ (2)	-
	sulfite reductase	CysI / Sir	+ (1)	-	+ (1)	-	+ (1)
Dissimilatory sulfide oxidation	dissimilatory adenylylsulfate reductase (APSR)	AprA	-	-	-	-	-
		AprB	-	-	-	-	-
	ATP:sulfate adenylyltransferase (ATPS)	Sat*	+ (1)	+ (1)	+ (1)	+ (1)	+ (1)
Sulfur reduction	polysulfide reductase (PSR)	PsrA*	+ (1)	+ (1)	+ (1)	+ (3)	+ (2)
		PsrB*	+ (1)	+ (1)	+ (1)	+ (1)	+ (2)
		PsrC* / NrfD*	+ (1)	+ (1)	+ (1)	+ (1)	+ (1)
Dissimilatory nitrate/nitrite reduction	periplasmatic nitrate reductase	NapA	+ (1)	+ (1)	+ (1)	+ (1)	-
		NapB	+ (1)	+ (1)	+ (1)	+ (1)	-
	nitrite reductase	NirS	+ (1)	+ (1)	+ (1)	+ (1)	-
	membrane-bound nitric oxide reductase	NorB	+ (1)	+ (1)	+ (1)	+ (1)	-
Assimilatory nitrate/nitrite reduction	Nitrate/nitrite transporter	NarK	+ (2)	-	+ (1)	+ (1)	+ (1)
	ferredoxin--nitrate reductase	NarB	-	-	+ (1)	+ (1)	+ (1)
	ferredoxin--nitrite reductase	NirA	+ (1)	+ (1)	+ (2)	+ (2)	+ (1)
rTCA cicle	Oxoglutarate:ferredoxin oxireductase (OOR)	OorA	+ (1)	+ (2)	+ (2)	+ (1)	+ (1)
		OorB	+ (1)	+ (2)	+ (2)	+ (1)	+ (1)
		OorC	+ (1)	+ (1)	+ (1)	+ (1)	+ (1)
		OorD	+ (1)	+ (1)	+ (1)	+ (1)	+ (1)
		OorE	-	-	-	-	+ (1)
	Pyruvate:ferredoxin oxireductase (POR)	PorA	+ (1)	+ (1)	+ (1)	+ (1)	+ (1)
		PorB	+ (1)	+ (1)	+ (1)	+ (1)	+ (1)
		PorC	+ (1)	+ (1)	+ (1)	+ (1)	+ (1)
		PorD	+ (1)	+ (1)	+ (1)	+ (1)	+ (1)
		PorE	-	-	-	-	+ (1)
	ATP-dependent citrate lyase (ACL)	AclA*	+ (1)	+ (1)	+ (1)	+ (1)	+ (1)
		AclB*	+ (1)	+ (1)	+ (1)	+ (1)	+ (1)
	Fumarate reductase	FdrA*	+ (2)	+ (2)	+ (3)	+ (2)	+ (3)
		FdrB*	+ (2)	+ (2)	+ (2)	+ (2)	+ (2)
		FdrC*	+ (1)	+ (1)	+ (2)	+ (1)	+ (1)
Oxygen reduction	Cytochrome *c* oxidase, cbb3-type	CcoN	+ (1)	+ (1)	+ (1)	+ (1)	+ (2)
		CcoO	+ (1)	+ (1)	+ (1)	+ (1)	+ (2)
		CcoQ	+ (1)	+ (1)	+ (1)	+ (1)	+ (2)
		CcoP	+ (1)	+ (1)	+ (1)	+ (1)	+ (2)
	Cytochrome *c* oxidase, caa3-type	QoxA / CoxA / CtaD	+ (1)	-	-	+ (1)	+ (1)
		QoxB / CoxB / CtaC	-	-	-	-	+ (1)
		QoxC / CoxC / CtaE	-	-	-	-	+ (1)
		QoxD / CoxD	-	-	-	-	+ (1)
Oxygen binding / sensing	Bacteriohemerythrin	Hhe-like*	+ (2)	+ (1)	+ (5)	+ (1)	+ (2)
Oxidative stress	Iron/manganese Superoxide dismutase	Sod2	+ (1)	+ (1)	+ (1)	+ (1)	-
	Superoxide reductase	1Fe-SOR*	+ (1)	+ (1)	+ (1)	+ (1)	+ (1)
		TAT-1Fe-SOR	-	-	-	-	+ (1)
	Catalase		+ (1)	+ (2)	-	-	-
	Peroxiredoxin (thiol peroxidase)	Tpx*	+ (4)	+ (4)	+ (4)	+ (3)	+ (3)
	Di-haem cytochrome c peroxidase	Ccp	+ (2)	+ (3)	+ (1)	+ (4)	+ (1)
Iron and manganese assimiliation	Fe^{2+} uptake	FeoA/FeoB*	+ (2)	+ (2)	+ (2)	+ (4)	+ (2)
	Mn^{2+} / Zn^{2+} uptake	ZucA / ZnuB* / ZnuC*	+ (3)	+ (3)	+ (3)	+ (3)	+ (3)
	Siderophore uptake	TonB / ExbB* / ExbD*	+ (9)	+ (9)	+ (7)	+ (8)	+ (6)
	Iron uptake regulation	Fur*	+ (1)	+ (3)	+ (2)	+ (2)	+ (1)
	Iron storage (Ferritin)	Cft	+ (1)	+ (1)	-	-	+ (1)
	Ferrous-iron efflux	FieF*	+ (1)	+ (1)	+ (1)	+ (2)	+ (1)

Sd: *Sulfurimonas denitrificans*; Sh: *Sulfurimonas hongkongensis*; Sg: *Sulfurimonas gotlandica*; Sa: *Sulfurimonas autotrophica*; Sp1: ^{U}*Sulfurimonas pluma* MAG-1.
* Core genes.

Supplementary Table 6. List of stations and sequencing approaches applied in this study.

Ridge	Area	Station	Sampling Date	Latitude	Longitude	Depth (m)	Water type	Sampling Device	Sequencing	ENA run accession
Indian	South-West	PS81-631	26.11.13	-52.438	13.279	2500	Background	CTD	16S iTag	ERR2222745
		PS81-631	26.11.13	-52.438	13.279	4130	Background	CTD	16S iTag	ERR2222746
		PS81-631	26.11.13	-52.438	13.279	4320	Background	CTD	16S iTag	ERR2222747
		PS81-645	30.11.13	-52.233	15.738	4119	Background	CTD	16S iTag	ERR2222750
		PS81-645	30.11.13	-52.233	15.738	4154	Background	CTD	16S iTag	ERR2222751
		PS81-645	30.11.13	-52.233	15.738	4169	Background	CTD	16S iTag	ERR2222752
		PS81-663	06.12.13	-52.646	12.526	2501	Background	CTD	16S iTag	ERR2222748
		PS81-663	06.12.13	-52.646	12.526	3651	Background	CTD	16S iTag	ERR2222749
Gakkel	Aurora	PS86-23	13.07.14	82.896	-6.247	3290	Plume	CTD	16S iTag	ERR7132512
		PS86-23	13.07.14	82.896	-6.247	3955	Bottom water	CTD	16S iTag	ERR7132511
		PS86-33	15.07.14	82.899	-6.334	2690	Above Plume	In situ pump	MT, MG	ERR7132538, ERR7132550
		PS86-33	15.07.14	82.899	-6.334	3182	Background	In situ pump	MT, MG	ERR7132537, ERR7132549
		PS86-40	17.07.14	82.918	-6.377	3183	Plume	In situ pump	MT, MG	ERR7132536, ERR7132548
		PS86-43	18.07.14	82.908	-6.233	3004	Plume	In situ pump	MT, MG	ERR7132535, ERR7132547
		PS86-52	22.07.14	82.909	-6.091	3200	Internal Reference	CTD	16S iTag	ERR7132510
		PS86-55	22.07.14	82.901	-6.254	2501	Above Plume	CTD	16S iTag	ERR7132509
		PS86-55	22.07.14	82.901	-6.254	2478	Above Plume	In situ pump	MT, MG	ERR7132534, ERR7132546
		PS86-55	22.07.14	82.901	-6.254	3400	Plume	CTD	16S iTag	ERR7132508
		PS86-55	22.07.14	82.901	-6.254	2958	Plume	In situ pump	MT, MG	ERR7132533, ERR7132545
		PS86-55	22.07.14	82.901	-6.254	3949	Bottom water	CTD	16S iTag	ERR7132507
		PS86-57	22.07.14	82.897	-6.232	2000	Above Plume	CTD	16S iTag	ERR7132506
		PS86-57	22.07.14	82.897	-6.232	3360	Plume	CTD	16S iTag	ERR7132505
		PS86-57	22.07.14	82.897	-6.232	3500	Below Plume	CTD	16S iTag	ERR7132504
		PS86-66	25.07.14	82.897	-6.279	2500	Above Plume	CTD	16S iTag	ERR7132503
		PS86-66	25.07.14	82.897	-6.279	3575	Plume	CTD	16S iTag	ERR7132502
		PS86-66	25.07.14	82.897	-6.279	3933	Bottom water	CTD	16S iTag	ERR7132501
		PS86-69	26.07.14	82.899	-6.269	2900	Above Plume	CTD	16S iTag	ERR7132500
		PS86-69	26.07.14	82.899	-6.269	3517	Plume	CTD	16S iTag	ERR7132499
		PS86-74	27.07.14	83.105	-2.466	2500	External Reference	CTD	16S iTag	ERR7132498
		PS86-74	27.07.14	83.105	-2.466	2983	External Reference	CTD	16S iTag	ERR7132497
Gakkel	Polaris	PS101-55	14.09.16	85.292	60.179	2070	External Reference	In situ pump	MT	ERR7132532
		PS101-55	15.09.16	85.293	60.184	3054	External Reference	CTD	16S iTag	ERR7132496
		PS101-55	14.09.16	85.292	60.179	3060	External Reference	In situ pump	MT	ERR7132531
		PS101-55	14.09.16	85.292	60.179	3070	External Reference	In situ pump	MT	ERR7132530
		PS101-55	14.09.16	85.292	60.179	3870	External Reference	In situ pump	MT	ERR7132529
		PS101-112	20.09.16	86.984	58.221	2051	Internal Reference	In situ pump	MT	ERR7132528
		PS101-112	21.09.16	86.984	58.237	3042	Internal Reference	CTD	16S iTag	ERR7132495
		PS101-112	20.09.16	86.984	58.221	3051	Internal Reference	In situ pump	MT	ERR7132527
		PS101-112	20.09.16	86.984	58.221	4051	Internal Reference	In situ pump	MT	ERR7132526
		PS101-112	20.09.16	86.984	58.221	4841	Internal Reference	In situ pump	MT	ERR7132525
		PS101-139	23.09.16	86.952	55.601	2338	Above Plume	CTD	16S iTag	ERR7132494
		PS101-139	23.09.16	86.952	55.594	2574	Plume	CTD	16S iTag	ERR7132493
		PS101-139	23.09.16	86.951	55.580	3107	Below Plume	CTD	16S iTag	ERR7132492
		PS101-139	23.09.16	86.951	55.575	3282	Bottom water	CTD	16S iTag	ERR7132491
		PS101-159	28.09.16	86.953	55.927	2336	Above Plume	CTD	16S iTag	ERR7132490
		PS101-159	28.09.16	86.953	55.935	2604	Plume	CTD	16S iTag	ERR7132489
		PS101-159	28.09.16	86.952	55.919	2653	Plume	CTD	16S iTag	ERR7132488
		PS101-159	28.09.16	86.954	55.941	2827	Plume	CTD	16S iTag	ERR7132487
		PS101-159	28.09.16	86.955	55.957	2846	Plume	CTD	16S iTag	ERR7132486
		PS101-159	28.09.16	86.951	55.907	3006	Below Plume	CTD	16S iTag	ERR7132485
		PS101-159	28.09.16	86.948	55.855	3150	Bottom water	CTD	16S iTag	ERR7132484
		PS101-177	03.10.16	86.959	55.675	2640	Plume	CTD	16S iTag	ERR7132483
		PS101-177	03.10.16	86.955	55.679	2666	Plume	CTD	16S iTag	ERR7132482
		PS101-177	03.10.16	86.957	55.677	2870	Below Plume	CTD	16S iTag	ERR7132556
		PS101-177	03.10.16	86.954	55.680	3136	Bottom water	CTD	16S iTag	ERR7132555
		PS101-181	03.10.16	86.951	55.740	2425	Above Plume	In situ pump	MT	ERR7132524
		PS101-181	03.10.16	86.951	55.740	2635	Plume	In situ pump	MT, MG	ERR7132523, ERR7132544
		PS101-181	03.10.16	86.951	55.740	2645	Plume	In situ pump	MT	ERR7132522
		PS101-181	03.10.16	86.951	55.740	2665	Plume	In situ pump	MT	ERR7132521
		PS101-188	05.10.16	86.949	55.678	2425	Above Plume	In situ pump	MT	ERR7132520
		PS101-188	05.10.16	86.968	55.951	2492	Above Plume	CTD	16S iTag	ERR7132554
		PS101-188	05.10.16	86.949	55.678	2635	Plume	In situ pump	MT, MG	ERR7132519, ERR7132543
		PS101-188	05.10.16	86.949	55.678	2645	Plume	In situ pump	MT, MG	ERR7132518, ERR7132542
		PS101-188	05.10.16	86.949	55.678	2665	Plume	In situ pump	MT, MG	ERR7132517, ERR7132541
		PS101-188	05.10.16	86.961	55.833	2716	Plume	CTD	16S iTag	ERR7132553
		PS101-188	05.10.16	86.967	55.922	3056	Below Plume	CTD	16S iTag	ERR7132552
		PS101-226	10.10.16	86.961	55.691	3219	Bottom water	CTD	16S iTag	ERR7132551
		PS101-230	10.10.16	86.945	55.673	3360	Bottom water at vents	In situ pump	MT, MG	ERR7132516, ERR7132540
		PS101-230	10.10.16	86.945	55.673	3379	Bottom water at vents	In situ pump	MT, MG	ERR7132515, ERR7132539
		PS101-230	10.10.16	86.945	55.673	3380	Bottom water at vents	In situ pump	MT	ERR7132514
		PS101-230	10.10.16	86.945	55.673	3390	Bottom water at vents	In situ pump	MT	ERR7132513

16S iTag: 16S rRNA gene amplicon Illumina sequencing (MiSeq); MT: metatranscriptomes, single-end Illumina sequencing (HiSeq); MG: metagenomes, pared-end Illumina sequencing (MiSeq). Background seawater: seawater without physico-chemical signatures for hydrothermal plume, but potentially affected by hydrothermal plume. Reference seawater: seawater without physico-chemical signatures for hydrothermal plume, and not affected by hydrothermal plume.

Supplementary Table 7. Data sets used for oligotyping of *Sulfurimonas*, including accession number, sample origin, and related publication if available.

ENA study accession	Geographic region	Sequences obtained from	Reference (DOI)
PRJEB10576	Sweden	Author	Hubalek et al. 2016 (doi:10.1038/ismej.2016.94)
ERP013833	Baltic Sea	ENA	Hu et al. 2016 (doi: 10.3389/fmicb.2016.00679)
ERP017367	Manus Basin	Author	Meier et al. 2017 (doi:10.1038/ismej.2017.37)
PRJEB23972	South-West Indian Ridge	Author	This study
PRJEB48226	Polaris Vent Field	Author	This study
PRJEB48226	Aurora Vent Field	Author	This study
PRJEB43086	Fram Strait	Author	unpublished
PRJEB11384	Papua New Guinea	Author	Hassenrück et al. 2016 (doi: 10.1093/femsec/fiw027)
PRJEB14127	Papua New Guinea	Author	Hassenrück et al. 2017 (doi.org/10.1093/icesjms/fsw204)
PRJEB42060-PRJEB42159	North Sea	Author	Miksch et al. 2021 (doi.org/10.1038/s43705-021-00028-w)
PRJEB18774	North Sea	Author	Probandt et al. 2018 (doi:10.1038/ismej.2017.197)
PRJEB23821	South-West Indian Ridge	Author	Varliero et al. 2019 (doi.org/10.3389/fmicb.2019.00665)
PRJEB27168	Galapagos Archipelago	ENA	Guevara et al. 2018 (doi.org/10.7717/peerj.5984)
PRJEB31776	Luso Hydrothermal Vent Field	ENA	NA
PRJEB32776	Gulf of Mexico	Author	Laso-Perez et al. 2019 (doi.org/10.1128/mBio.01814-19)
PRJEB32776	Gulf of Mexico	Author	Wegner unpublished
PRJEB33205	South-East Pacific	Author	Krause et al. 2020 (doi: 10.1038/s41598-020-66361-7)
PRJEB7448	Baltic Sea	ENA	unpublished
PRJNA282077	France, Portugal	ENA	Cucio et al. 2016 (doi.org/10.3389/fmicb.2016.00440)
PRJNA299110	Okinawa Trench	ENA	Sun et al. 2016 (doi.org/10.1371/journal.pone.0154359)
PRJNA318932	Okinawa Trench	ENA	Wang et al. 2016 (doi.org/10.1016/j.jmarsys.2016.11.012)
PRJNA322450	Baltic Sea	ENA	Broman et al. 2017 (DOI 10.1186/s40168-017-0311-5)
PRJNA330786	Manus Basin	ENA	unpublished
PRJNA331054	California Bight	ENA	unpublished
PRJNA341261	North Atlantic Subtropical Gyre	ENA	Shoemaker et al. 2019 (doi:10.1111/1462-2920.14723)
PRJNA349764	South China Sea	ENA	Zhang et al. 2017 (doi.org/10.1016/j.margeo.2017.06.010)
PRJNA352433	Mariana Arc	ENA	Hager et al. 2017 (doi.org/10.3389/fmicb.2017.01578)
PRJNA360358	North Sea	ENA	De Tender et al. 2017 (doi.org/10.1021/acs.est.7b00697)
PRJNA379939	Dominica Island	Author	Gomez-Saez et al. 2017 (doi.org/10.1038/s41598-018-37914-8)
PRJNA386676	Dominica Island	ENA	Pop-Ristova et al. 2017 (doi.org/10.3389/fmicb.2017.02400)
PRJNA401268	Mid Atlantic	ENA	Coykendall et al. 2019 (doi.org/10.1371/journal.pone.0211616)
PRJNA414441	Indian Ridge	ENA	Han et al. 2018 (DOI:10.1038/s41598-018-28613-5)
PRJNA434752	Gulf of Mexico	ENA	Godoy-Lozano et al. 2018 (doi.org/10.3389/fmicb.2018.00279)
PRJNA485064	Gulf of Cadiz	ENA	Rincon-Tomas et al. 2019 (doi.org/10.5194/bg-16-1607-2019)
PRJNA498402	Southern India	ENA	Rajeev et al. 2019 (doi.org/10.1016/j.scitotenv.2019.02.171)
PRJNA511010	Gulf of Mexico	ENA	unpublished
PRJNA524261	North China	ENA	Liang et al. 2019 (doi.org/10.1016/j.scitotenv.2019.07.200)
PRJNA549457	Loihi Seamount	ENA	unpublished
PRJNA563517	Indian Ridge, Mid-Atlantic Ridge, East-Pacific Rise	ENA	unpublished
PRJNA564579	Okinawa Island	ENA	Ares et al. 2019 (doi.org/10.1101/801886)

NA: not available

Supplementary Table 8. Groups of amplicon Illumina sequencing data sets based on environmental categories used in oligotyping analysis to identify ecotypes.

Salinity	Zone	Water depth	Hydrothermal influence	Artificial environment
Fresh water	Subsurface		none	
Brackish	Benthic	Coastal	none	
Marine	Benthic	Coastal	none	
	Benthic	Coastal	none	Plastic
	Benthic	Coastal	HV-substrate	
	Benthic	Deep-sea	none	
	Benthic	Deep-sea	none	Plastic
	Benthic	Deep-sea	HV-fluid	
	Benthic	Deep-sea	HV-substrate	
	Pelagic	Deep-sea	HV-plume	
	Pelagic	Deep-sea	none	Sediment trap

Supplementary Table 9. a, information for isolate genome and MAG. **b**, information for hydrothermal vent meta-genomes and -transcriptomes used in this study.

a

Name	Source	GenBank assembly accession	GenBank genome accession	Total length	Number of contigs	Completeness (%)	Redundancy (%)	Isolation source
Sulfurimonas autotrophica, strain DSM 16294	Culture	GCA 000147355	CP002205	2,153,198	1	100	1.7	Deep-sea sediments at the Hatoma Knoll in the Mid-Okinawa Trough hydrothermal field (Pacific Ocean)
Sulfurimonas hongkongensis, strain AST-10	Culture	GCA 000445475	na	2,302,023	28	100	1.7	Coastal sediment; Hong Kong (China)
Sulfurimonas gotlandica, strain GD1	Culture	GCA 000242915	AFRZ01000001	2,952,682	1	100	0.0	Sulfidic area of central Baltic Sea station 271 at 215 m water depth
Sulfurimonas gotlandica, strain GD1	Culture	GCA 000156095	na	2,946,312	12	99	0.0	Type strain of Sulfurimonas gotlandica
Sulfurimonas denitrificans, strain DSM 1251	Culture	GCA 000012965	CP000153	2,201,561	1	98	3.5	Type strain of Sulfurimonas denitrificans
Sulfuricurvum kujiense, strain DSM 16994	Culture	GCF 000183725	na	2,819,357	5	98	1.7	Isolated from an underground crude-oil storage cavity at Kuji in Iwate (Japan)
Sulfuricurvum sp. IAE1	Culture	GCA 004347735	na	2,338,235	65	100	0.0	Sediments, Shenyang (China)
Sulfurimonas sp. NW8N	Culture	GCA 009192995	na	2,093,483	52	100	0.0	Chimney sulfide, 2928 m (Indian Ocean)
Sulfurimonas sp. NW10	Culture	GCA 009068765	na	2,342,011	1	100	1.7	Chimney sulfide, 2946 m (Indian Ocean)
Sulfurimonas crateris, strain SN118	Culture	GCA 005217605	na	2,209,279	11	98	1.7	Terrestrial mud volcano; Taman Peninsula (Russia)
Sulfurimonas sp. 1-1N	Culture	GCA 009258045	na	1,916,242	1	98	1.7	Sulfur enrichment, Mountain Gunyin (Fujian, China)
Sulfurimonas sp. NORP9	MAGs	GCA 002733945	na	2,494,119	24	98	1.7	Marine subsurface aquifer 70-332 mbsf, North pond (Atlantic Ocea)
Sulfurimonas sp. UBA2011	MAGs	GCA 002335055	na	1,596,058	32	98	1.7	Von Damm hydrothermal vent plume at depth 2041m, Mid Cayman Rise (Atlantic Ocean)
Sulfurimonas sp. GWF2_37_8	MAGs	GCA 001829625	na	1,906,285	227	93	0.0	Rifle well CD01 at time point 6 / F; 5m depth; 0.2 filter; Rifle, CO (USA)
Sulfurimonas sp. UBA6014	MAGs	GCA 002428085	na	2,192,659	74	97	1.7	Water, microbial mat metagenome
Sulfurimonas sp. CG12_big_fil_rev_8_21_14_0_65_36_1453	MAGs	GCA 002781885	na	2,414,593	109	100	0.0	Groundwater, 2 m depth; Crystal Geyser near Green River, Utah (USA)
Sulfurimonas sp. CG02_land_8_20_14_3_00_36_67	MAGs	GCA 002780815	na	2,406,264	98	100	0.0	Groundwater, 2 m depth; Crystal Geyser near Green River, Utah (USA)
Sulfurimonas sp. CG_4_9_14_0_8_um_filter_36_384	MAGs	GCA 002789515	na	2,425,098	108	100	0.0	Groundwater, 2 m depth; Crystal Geyser near Green River, Utah (USA)
Sulfurimonas sp. BM502	MAGs	GCA 002899765	na	2,391,647	45	98	0.0	Enrichment from estuary sediment; Berkeley, Ca, marina (USA)
Sulfurimonas sp. UBA6795	MAGs	GCA 002452895	na	2,043,644	50	97	3.5	Waste water; Medicine Hat Glauconitic field (Canada)
Sulfurimonas sp. BM702	MAGs	GCA 002869525	na	1,891,063	135	95	2.7	Enrichment from estuary sediment; Berkeley, Ca, marina (USA)
Sulfurimonas sp. RIFOXYD2_FULL_34_21	MAGs	GCA 001830805	na	2,360,463	15	100	1.7	Rifle well CD01 at 16ft depth, 1.2 micron filter at time point D; Rifle, CO (USA)
Sulfurimonas sp. RIFOXYD12_FULL_33_39	MAGs	GCA 001829785	na	2,342,198	13	100	1.7	Rifle well CD01 at 16ft depth, 1.2 micron filter at time point D; Rifle, CO (USA)
Sulfurimonas sp. RIFCSPLOWO2_12_FULL_34_6	MAGs	GCA 001829695	na	1,723,710	367	89	0.0	Rifle well FP-101 under low O2 conditions, 1.2 micron filter; Rifle, CO (USA)
Sulfurimonas sp. RIFOXYB12_FULL_35_9	MAGs	GCA 001829715	na	2,501,187	128	98	3.6	Rifle well CD01 at 16ft depth, 1.2 micron filter at time point B; Rifle, CO (USA)
Sulfurimonas sp. RIFCSPLOWO2_12_FULL_36_74	MAGs	GCA 001829705	na	1,865,831	444	90	0.9	Rifle well FP-101 under low O2 conditions, 1.2 micron filter; Rifle, CO (USA)
Sulfurimonas sp. RIFCSPHIGHO2_12_FULL_36_9	MAGs	GCA 001830775	na	1,815,804	141	91	0.0	Rifle well FP-101 under high O2 conditions, 1.2 micron filter; Rifle, CO (USA)
Sulfurimonas sp. RIFCSPLOWO2_12_36_12	MAGs	GCA 001829675	na	2,182,863	69	98	0.0	Rifle well FP-101 under low O2 conditions, 1.2 micron filter; Rifle, CO (USA)

b

Source	ENA run accession	Vent Site	Environmnet	Latitude	Longitude	depth (m)	Activity	Fluid Temperature	Tectonic setting	Ocean	Region	Category	Fluid type
metagenome	SRR2046235	Von Damm	plume	na	na	2041	Active	na	mid-ocean ridge	Caribbean Sea	Mid-Cayman Rise	ultra-slow spreading ridge	na
metagenome	SRR2046236	Von Damm	plume	na	na	2238	Active	na	mid-ocean ridge	Caribbean Sea	Mid-Cayman Rise	ultra-slow spreading ridge	na
metagenome	SRR7168053	Ruby	plume	15.48155	144.5971674	3625	Active	na	arc volcano	North Pacific	Mariana Arc	back-arc spreading center	Sulfide-rich
metagenome	SRR3577362	Southern Trough	plume	27.025	-111.4	na	Active	na	mid-ocean ridge	North Pacific	Guaymas Basin	slow spreading ridge	Methane-rich
metagenome	ERR2021511	Anemone (Axial Volcano, ASHES)	plume	45.9335667	-130.013667	1500	Active	348	mid-ocean ridge	North Pacific	Juan de Fuca Ridge	fast spreading ridge	Sulfide-rich
metagenome	SRR2046238	Beebe	plume	18.544783	-81.7195	4869	Active	398	mid-ocean ridge	Caribbean Sea	Mid-Cayman Rise	ultra-slow spreading ridge	Sulfide-Metal-rich
metagenome	SRR2080392	Beebe	plume	18.544783	-81.7195	4900	Active	398	mid-ocean ridge	Caribbean Sea	Mid-Cayman Rise	ultra-slow spreading ridge	Sulfide-Metal-rich
metagenome	SRR2046222	Von Damm	background seawater	na	na	2327	not applicable	not applicable	mid-ocean ridge	Caribbean Sea	Mid-Cayman Rise	ultra-slow spreading ridge	not applicable
metagenome	SRR2046237	Von Damm	background seawater	18.544783	-81.7195	4946	not applicable	not applicable	mid-ocean ridge	Caribbean Sea	Mid-Cayman Rise	ultra-slow spreading ridge	not applicable
metagenome	SRR2046221	Von Damm	background seawater	na	na	4950	not applicable	not applicable	mid-ocean ridge	Caribbean Sea	Mid-Cayman Rise	ultra-slow spreading ridge	not applicable
metagenome	ERS1370013	Old Man Tree (Von Damm)	seawarer hydrothermal vent	18.375	-81.7976	2373	Active	114	mid-ocean ridge	Caribbean Sea	Mid-Cayman Rise	ultra-slow spreading ridge	na
metagenome	ERS714917	Ginger Castle (Von Damm)	seawarer hydrothermal vent	18.3769	-81.7979	2308	Active	47	mid-ocean ridge	Caribbean Sea	Mid-Cayman Rise	ultra-slow spreading ridge	na
metagenome	ERS714918	Shrimp Gulley #2, (Piccard)	seawarer hydrothermal vent	18.5466	-81.7177	4940	Active	108	mid-ocean ridge	Caribbean Sea	Mid-Cayman Rise	ultra-slow spreading ridge	na
metagenome	ERS1370022	near Main Orifice (Von Damm)	seawarer hydrothermal vent	18.3767	-81.7981	2293	na	na	mid-ocean ridge	Caribbean Sea	Mid-Cayman Rise	ultra-slow spreading ridge	na
metagenome	ERS1370017	Main Orifice (Von Damm)	seawarer hydrothermal vent	18.3767	-81.7981	2297	Active	109	mid-ocean ridge	Caribbean Sea	Mid-Cayman Rise	ultra-slow spreading ridge	na
metagenome	ERS1370024	Twin Peaks (Von Damm)	seawarer hydrothermal vent	na	na	2369	Active	140	mid-ocean ridge	Caribbean Sea	Mid-Cayman Rise	ultra-slow spreading ridge	na
metagenome	ERS1370026	Hot Cracks #2 (Von Damm)	seawarer hydrothermal vent	18.375	-81.7974	2369	Active	29	mid-ocean ridge	Caribbean Sea	Mid-Cayman Rise	ultra-slow spreading ridge	na
metagenome	ERS1370025	Shrimp Buttery (Von Damm)	seawarer hydrothermal vent	na	na	2369	Active	131	mid-ocean ridge	Caribbean Sea	Mid-Cayman Rise	ultra-slow spreading ridge	na
metagenome	ERS1370027	Old Man Tree (Von Damm)	seawarer hydrothermal vent	18.375	-81.7976	2370	Active	114	mid-ocean ridge	Caribbean Sea	Mid-Cayman Rise	ultra-slow spreading ridge	na
metagenome	ERS1370023	Shrimp Hole (Von Damm)	seawarer hydrothermal vent	18.3747	-81.7973	2372	Active	50	mid-ocean ridge	Caribbean Sea	Mid-Cayman Rise	ultra-slow spreading ridge	na
metagenome	ERS1370015	Shrimp Hole (Von Damm)	seawarer hydrothermal vent	18.3747	-81.7973	2376	Active	50	mid-ocean ridge	Caribbean Sea	Mid-Cayman Rise	ultra-slow spreading ridge	na
metagenome	ERS1370014	Ravelin #2 (Von Damm)	seawarer hydrothermal vent	18.3752	-81.7971	2388	Active	86	mid-ocean ridge	Caribbean Sea	Mid-Cayman Rise	ultra-slow spreading ridge	na
metagenome	ERS1003593	Marker (Axial Volcano)	seawater	45.9892	-130.0272	1200	Active	292	mid-ocean ridge	North Pacific	Juan de Fuca Ridge	fast spreading ridge	Sulfide-rich
metagenome	ERR2021505	Anemone (Axial Volcano, ASHES)	diffuse fluid	45.9332	-130.0139	1541	Active	348	mid-ocean ridge	North Pacific	Juan de Fuca Ridge	fast spreading ridge	Sulfide-rich
metagenome	ERS1003599	Anemone (Axial Volcano, ASHES)	diffuse fluid	45.9892	-130.0272	1539	Active	291	mid-ocean ridge	North Pacific	Juan de Fuca Ridge	fast spreading ridge	Sulfide-rich
metagenome	ERR2021507	Marker (Axial Volcano)	diffuse fluid	45.9332	-129.9822	1518	Active	323	mid-ocean ridge	North Pacific	Juan de Fuca Ridge	fast spreading ridge	Sulfide-rich
metagenome	ERS1003595	Marker (Axial Volcano)	diffuse fluid	45.9892	-130.0272	1521	Active	291	mid-ocean ridge	North Pacific	Juan de Fuca Ridge	fast spreading ridge	Sulfide-rich
metagenome	ERS1003599	Marker (Axial Volcano)	diffuse fluid	45.9892	-130.0272	1514	Active	291	mid-ocean ridge	North Pacific	Juan de Fuca Ridge	fast spreading ridge	Sulfide-rich
metagenome	ERR2021503	Marker (Axial Volcano)	diffuse fluid	45.9266	-129.9795	1518	Active	323	mid-ocean ridge	North Pacific	Juan de Fuca Ridge	fast spreading ridge	Sulfide-rich
metagenome	ERS614331	Marker (Axial Volcano)	diffuse fluid	45.9892	-130.0272	1516	Active	291	mid-ocean ridge	North Pacific	Juan de Fuca Ridge	fast spreading ridge	Sulfide-rich
metagenome	ERS614325	Marker (Axial Volcano)	diffuse fluid	45.9892	-130.0272	1530	Active	291	mid-ocean ridge	North Pacific	Juan de Fuca Ridge	fast spreading ridge	Sulfide-rich
metagenome	ERS614333	Marker (Axial Volcano)	diffuse fluid	45.9892	-130.0272	1917	Active	291	mid-ocean ridge	North Pacific	Juan de Fuca Ridge	fast spreading ridge	Sulfide-rich
metagenome	ERR2021509	Marker (Axial Volcano)	diffuse fluid	45.9332	-129.9822	1716	Active	323	mid-ocean ridge	North Pacific	Juan de Fuca Ridge	fast spreading ridge	Sulfide-rich
metagenome	ERR1679395	North Su (Susu Knolls, Solwara 1)	fluid	-3.799916333	152.1008595	1199	Active	332	arc volcano	South Pacific	Manus Basin	back-arc spreading center	Metal-rich
metagenome	ERR1679396	Fenway (Pacmanus, Solwara 4)	fluid	-3.728297833	151.672495	1705	Active	276	arc volcano	South Pacific	Manus Basin	back-arc spreading center	Sulfide-Metal-rich
metagenome	ERR1679397	Fenway (Pacmanus, Solwara 4)	fluid	-3.728333	151.672394	1709	Active	276	arc volcano	South Pacific	Manus Basin	back-arc spreading center	Sulfide-Metal-rich
metagenome	ERR1679394	North Su (Susu Knolls, Solwara 1)	fluid	-3.799916333	152.1008595	1155	Active	332	arc volcano	South Pacific	Manus Basin	back-arc spreading center	Metal-rich
metagenome	ERR1679398	Roman Ruins (Pacmanus, Solwara 4)	fluid	-3.720639	151.675313	1685	Active	276	arc volcano	South Pacific	Manus Basin	back-arc spreading center	Metal-rich
metagenome	SRR10294826	Lost City	Chimney	30.125	-42.1183	na	Active	90	mid-ocean ridge	North Atlantic	Mid-Atlantic Ridge	slow spreading ridge	Hydrogen-rich
metagenome	SRR6439354	na	Chimney	-49.48	37.99	2328	na	na	mid-ocean ridge	IndianOcean	Southwest Indian Ridge	ultra-slow spreading ridge	na
metagenome	SRR9822614	Old City	Chimney	-27.843633	64.593817	3136	Active	na	mid-ocean ridge	IndianOcean	Southwest Indian Ridge	ultra-slow spreading ridge	Hydrogen-rich
metagenome	SRR6439370	Mothra	Chimney	47.9233	-129.1082	2182	Active	320	mid-ocean ridge	North Pacific	Juan de Fuca Ridge	fast spreading ridge	Sulfide-rich
metagenome	SRR6439364	na	Chimney	-15.16	-13.35	2194	na	na	mid-ocean ridge	South Atlantic	Mid-Atlantic Ridge	slow spreading ridge	Sulfide-Hydrogen-rich
metagenome	SRR6439362	na	Chimney	-15.1666	-13.3558	2891	na	na	mid-ocean ridge	South Atlantic	Mid-Atlantic Ridge	slow spreading ridge	Sulfide-Hydrogen-rich
metagenome	SRR6439358	na	Chimney	-49.48	37.99	1711	na	na	mid-ocean ridge	IndianOcean	Southwest Indian Ridge	ultra-slow spreading ridge	na
metagenome	SRR6439359	na	Chimney	-49.48	37.99	2328	na	na	mid-ocean ridge	IndianOcean	Southwest Indian Ridge	ultra-slow spreading ridge	na
metagenome	SRR9822615	na	Chimney	-51.73	37.47	1592	na	na	mid-ocean ridge	IndianOcean	Southwest Indian Ridge	ultra-slow spreading ridge	na
metagenome	SRR5560800	Deyin-1	Chimney	-15.16	-13.35	2770	Active	na	mid-ocean ridge	South Atlantic	Mid-Atlantic Ridge	slow spreading ridge	Sulfide-Hydrogen-rich
metagenome	ERR2639421	Satanic Mills (Solwara 4)	inactive solid surface 1 (mineral)	-3.726833	151.672144	1689	na	na	arc volcano	South Pacific	Manus Basin	back-arc spreading center	Metal-rich
metagenome	SRR7968113	Lucky Strike	sulfide rock	37.2904	-32.2765	na	Active	333	mid-ocean ridge	North Atlantic	Mid-Atlantic Ridge	slow spreading ridge	Sulfide-Hydrogen-rich
metagenome	SRR7968105	na	sulfide rock	-22.1802	-176.6008	na	na	309 to 363°C	arc volcano	South Pacific	Lau Basin	back-arc spreading center	Sulfide-rich
metagenome	SRR8439171	na	sulfide rock	9.8384	-104.2913	na	Active	386	mid-ocean ridge	South Pacific	East Pacific Rise	fast spreading ridge	Sulfide-rich
metagenome	SRR8542060	Main Endeavour	sulfide rock	47.9487	-129.0981	na	Active	402	mid-ocean ridge	North Pacific	Juan de Fuca Ridge	fast spreading ridge	Sulfide-rich
metagenome	SRR7968109	Southern Trough	sulfide rock	27.025	-111.4	na	na	na	mid-ocean ridge	North Pacific	Guaymas Basin	slow spreading ridge	Methane-rich
metagenome	SRR8439172	Lucky Strike	sulfide rock	37.2917	-32.2779	na	Active	333	mid-ocean ridge	North Atlantic	Mid-Atlantic Ridge	slow spreading ridge	Sulfide-Hydrogen-rich
metagenome	SRR8439019	na	sulfide rock	-20.761	-176.1909	na	na	309 to 363°C	arc volcano	South Pacific	Lau Basin	back-arc spreading center	Sulfide-rich
metagenome	SRR8439053	na	sulfide rock	-21.9879	-176.5676	na	na	309 to 363°C	arc volcano	South Pacific	Lau Basin	back-arc spreading center	Sulfide-rich
metagenome	SRR7968125	Southern Trough	sulfide rock	27.0148	-111.4109	na	active	359	mid-ocean ridge	North Pacific	Guaymas Basin	slow spreading ridge	Methane-rich
metagenome	SRR5560801	Deyin-1	Sulfide sedimnents	-15.16	-13.35	2730	Active	na	high	South Atlantic	Mid-Atlantic Ridge	slow spreading ridge	Sulfide-Hydrogen-rich
metagenome	SRR7216773	Deyin-1	Sulfide sedimnents	-15.1666	-13.3558	2721	Active	na	mid-ocean ridge	South Atlantic	Mid-Atlantic Ridge	slow spreading ridge	Sulfide-Hydrogen-rich
metagenome	SRR7207567	Deyin-1	Sulfide sedimnents	-15.1666	-13.3558	2807	Active	na	mid-ocean ridge	South Atlantic	Mid-Atlantic Ridge	slow spreading ridge	Sulfide-Hydrogen-rich
metagenome	SRR7168054	Perseverance (Limpet Canyon)	na	15.47989247	144.5075395	3913	Active	265	arc volcano	North Pacific	Mariana Trough	back-arc spreading center	Sulfide-rich
metagenome	SRR7168051	Alba (Hafa Adai)	na	16.957	144.868	3277	Active	339	arc volcano	North Pacific	Mariana Trough	back-arc spreading center	Sulfide-rich
metagenome	SRR7168042	Hula Array (Hafa Adai)	na	16.957	144.868	3278	Active	339	arc volcano	North Pacific	Mariana Trough	back-arc spreading center	Sulfide-rich
metagenome	SRR7168048	Snail 001 (Alice Springs Field)	na	18.2103	144.7073	3611	Active	287	arc volcano	North Pacific	Mariana Trough	back-arc spreading center	Sulfide-rich
metagenome	SRR7168049	Snail Pit (18.2 N)	na	18.108	144.738	3631	Active	na	arc volcano	North Pacific	Mariana Trough	back-arc spreading center	Sulfide-rich
metatranscriptome	SRR7168043	Ruby	Plume	15.48155	144.5971674	3625	Active	na	arc volcano	North Pacific	Mariana Trough	back-arc spreading center	Sulfide-rich
metatranscriptome	SRR452448	Guaymas Basin	Plume	na	na	1950			mid-ocean ridge	North Pacific	Guaymas Basin	slow spreading ridge	Methane-rich
metatranscriptome	SRR453184	Guaymas Basin	background seawater	na	na	na	na	na	mid-ocean ridge	North Pacific	Guaymas Basin	slow spreading ridge	Methane-rich
metatranscriptome	ERS1372603	Old Man Tree (Von Damm)	seawarer hydrothermal vent	18.375	-81.7976	2373	Active	114	mid-ocean ridge	Caribbean Sea	Mid-Cayman Rise	ultra-slow spreading ridge	na
metatranscriptome	ERS1372604	Shrimp Hole (Von Damm)	seawarer hydrothermal vent	18.3747	-81.7973	2376	Active	50	mid-ocean ridge	Caribbean Sea	Mid-Cayman Rise	ultra-slow spreading ridge	na
metatranscriptome	ERS1372610	Shrimp Hole (Von Damm)	seawarer hydrothermal vent	18.3747	-81.7973	2372	Active	50	mid-ocean ridge	Caribbean Sea	Mid-Cayman Rise	ultra-slow spreading ridge	na
metatranscriptome	ERS1372612	Old Man Tree (Von Damm)	seawarer hydrothermal vent	18.375	-81.7976	2370	Active	114	mid-ocean ridge	Caribbean Sea	Mid-Cayman Rise	ultra-slow spreading ridge	na
metatranscriptome	ERS1372605	Ginger Castle (Von Damm)	seawarer hydrothermal vent	18.376929	-81.797906	2308	Active	47	mid-ocean ridge	Caribbean Sea	Mid-Cayman Rise	ultra-slow spreading ridge	na
metatranscriptome	ERS1372611	Twin Peaks (Von Damm)	seawarer hydrothermal vent	na	na	2369	Active	140	mid-ocean ridge	Caribbean Sea	Mid-Cayman Rise	ultra-slow spreading ridge	na
metatranscriptome *	4551443.3	Axial Caldera (ASHES)	diffusive fluid	-45.933352	130.013697	3495	na	na	mid-ocean ridge	North Pacific	Juan de Fuca Ridge	fast spreading ridge	Sulfide-rich
metatranscriptome *	4551453.3	Axial Caldera (ASHES)	seawater	-45.933352	130.013697	3060	na	na	mid-ocean ridge	North Pacific	Juan de Fuca Ridge	fast spreading ridge	Sulfide-rich
metatranscriptome *	4551455.3	Axial Caldera (ASHES)	diffusive fluid	-45.933352	130.013697	3234	na	na	mid-ocean ridge	North Pacific	Juan de Fuca Ridge	fast spreading ridge	Sulfide-rich
metatranscriptome *	4565465.3	Axial Caldera (ASHES)	intra-filed fluid	-45.933352	130.013697	3007	na	na	mid-ocean ridge	North Pacific	Juan de Fuca Ridge	fast spreading ridge	Sulfide-rich
metatranscriptome *	4565467.3	Axial Caldera (ASHES)	diffusive fluid	-45.933352	130.013697	3494	na	na	mid-ocean ridge	North Pacific	Juan de Fuca Ridge	fast spreading ridge	Sulfide-rich
metatranscriptome *	4565470.3	Axial Caldera (ASHES)	intra-filed fluid	-45.933352	130.013697	3239	na	na	mid-ocean ridge	North Pacific	Juan de Fuca Ridge	fast spreading ridge	Sulfide-rich
metatranscriptome *	4565471.3	Axial Caldera (ASHES)	diffusive fluid	-45.933352	130.013697	3471	na	na	mid-ocean ridge	North Pacific	Juan de Fuca Ridge	fast spreading ridge	Sulfide-rich

* downloaded from MG-RAST

* downloaded from MG-RAST; na: not available

Supplementary Notes

Supplementary Note 1: Hydrogen oxidation in the presence of oxygen. The presence and high expression of genes encoding for the membrane-bound group 1b [NiFe]-hydrogenase suggest that hydrogen is the main energy source for ^{U}*S. pluma* in the plume (**Table 1** and **Fig. 2**). This type of hydrogenase is present in all genomes of isolated *Sulfurimonas* strains[13] (**Supplementary Table 5**) with the exception of *S. paralvinellae*[14]. Neither *S. autotrophica* nor natural *Sulfurimonas* populations from hydrothermal vents have been shown to oxidize hydrogen under aerobic condition[15,14]. Yet, others *Sulfurimonas* strains from hydrothermal vents and some isolates closest related to *S. autotrophica* (99.9% 16S rRNA gene sequence similarity) obtained from Iheya North deep-sea hydrothermal field oxidized H_2 using O_2 as electron acceptor at a temperature of 25−37 °C[17,14]. Our findings showed for the first time overexpression of the genes for the membrane-bound [NiFe]-hydrogenase by natural populations of *Sulfurimonas* permanently exposed to O_2 concentrations > 300 µM and to temperatures < 0 °C. Group 1b [NiFe]-hydrogenase is described as the prototypical oxygen-sensitive hydrogenase[18]. It is therefore interesting that hydrothermal plume ^{U}*S. pluma* uses this enzyme under aerobic conditions and low hydrogen concentrations (i.e. nM), and does not have other types of hydrogenases more tolerant to oxygen, like group 1d[19] found in another hydrogen-oxidizing chemolithotrophs living in hydrothermal plumes (i.e. SUP05)[20], and group 2d present in *S. denitrificans*, *S. hongkongensis*, *S. gotlantica* and in some *Sulfurimonas* isolates from hydrothermal vents suggested to be relevant under low hydrogen concentrations[13,14]. The ability of ^{U}*S. pluma* to use hydrogen as electron donor in the presence of oxygen seems not to be related to chemical and structural features of the hydrogenase, but rather to the presence of effective O_2 protection systems that reduce

the cytoplasmic O_2 concentrations (i.e. cytochrome aa3 oxidase, antioxidant enzymes) or availability (i.e. hemerythrin).

Supplementary Note 2: Sulfide oxidation pathways. Two main pathways for sulfide oxidation exist in chemolitotrophic bacteria: one involving a sulfide:quinone reductase (SQR) and a second one involving a two subunit complex called flavocytochrome c sulfide dehydrogenase (FCC). This FCC complex consists of two subunits: a flavoprotein (fccB) and a cytochrome c (fccA). Previous studies have only reported the presence of genes encoding for five different types of SQR (Types II − VI) in known *Sulfurimonas* genomes[13] (**Supplementary Table 5**), suggesting that this enzyme is crucial for sulfide oxidation in this genus. Furthermore, sulfide oxidation by SQR provides more energy than sulfide oxidation by FCC[21], suggesting the SQR is more suitable for growing in the hydrothermal plumes, as documented for other plume sulfur-oxidizing bacteria[20]. Yet, ^{U}*S. pluma*' MAGs contain only one gene encoding for the SQR Type VI (SplumaMAG1_01467 and SplumaMAG2_00985), which is shorter than it should be and showed very low levels of expression (**Fig. 2**). The synteny between ^{U}*S. pluma* and *S. autotrophica* showed that SQR Type VI is not truncated, as suggested by conserved sequence regions at both ends of the gene and because the gene is flanked by conserved genes.

Instead, ^{U}*S. pluma* contains a gene (SplumaMAG1_00665 and SplumaMAG1_01610) annotated as flavocytochrome c sulfide dehydrogenase (*fccB*), which highly expressed in Gakkel hydrothermal plumes (**Fig. 2**). We checked the presence of *fccB* in previous *Sulfurimonas* genomes, and we could detect the presence of previously unreported *fccB* genes in them (except for *S. autotrophica*), usually annotated as FAD-dependent oxidoreductase (**Table 1**). In all of these genomes, including ^{U}*S. pluma*, the

cytochrome c subunit (*fccA*) was missing. Since *fccB* and *SQR* both belong to Group I flavoprotein disulfide reductases (FDR)[22], we decided to build a phylogenetic tree for their amino acid sequences to resolve the position of the *fccB* genes (**Extended data Fig. 6**). The *fccB* sequences of ^{U}*S. pluma* form a cluster with other fccB sequences from *Sulfurimonas hongkongensis* and *Sulfurovum*. This cluster is closely related to a second cluster of *Sulfurimonas fccB* that also contains sequences from *Aquifex aeolicus* and *Arcobacter mytili*. These two clusters are more related to the canonical *fccB* than to the SQR, but they do not have a *fccA* subunit. However adjacent to *fccB* gene there is a protein annotated as small subunit of arsenite:cytochrome c oxidoreductase (*aioB*, 28.7% amino acid identity), involved in the oxidation of arsenic[23]. This protein has a Rieske 2fe-2S cluster domain that would transfer arsenite-derived electron from the molybdenum centre of the catalytic subunit (*aioB*) to the cytochrome c[24]. This gene is present in all members of putative FCC clusters, thus we hypothesize that it may act as the electron carrier for f*ccB*. However, we cannot exclude that *fccB* transfer the electron directly to the quinone pool as in the canonical SQR, as well as unknown function for *fccB* and *aioB*. Therefore, whether this non-canonical *fccB* is involved in sulfide oxidation and whether the electron can be used to generate only proton motive force or also reducing power for carbon dioxide fixation remain open questions.

A further relevant question is why ^{U}*S. pluma* has lost canonical SQRs, typical of all other members of *Sulfurimonas* and hydrothermal plume sulfur-oxidizing SUP05 (**Extended data Fig. 6**), in favor of a new SQR or a non-canonical FCC. The similarity between *fccB* and *aioA* of ^{U}*S. pluma* and sequences from other aerobic environments (i.e. aerobic marine sediment enrichment[25] and oxic subsurface aquifer;

Extended data Fig. 6) suggest that the acquisition of this enzyme may be an adaptation to aerobic environments.

References

1. Broman, E., Sjöstedt, J., Pinhassi, J. & Dopson, M. Shifts in coastal sediment oxygenation cause pronounced changes in microbial community composition and associated metabolism. *Microbiome* **5**, 96 (2017).
2. Probandt, D. *et al.* Permeability shapes bacterial communities in sublittoral surface sediments. *Environ. Microbiol.* **19**, 1584–1599 (2017).
3. Hubalek, V. *et al.* Erratum: Connectivity to the surface determines diversity patterns in subsurface aquifers of the Fennoscandian shield. *ISME J.* **10**, 2556 (2016).
4. Hager, K. W., Fullerton, H., Butterfield, D. A. & Moyer, C. L. Community Structure of Lithotrophically-Driven Hydrothermal Microbial Mats from the Mariana Arc and Back-Arc. *Frontiers in Microbiology* **8**:1578 (2017).
5. Meier, D. V *et al.* Niche partitioning of diverse sulfur-oxidizing bacteria at hydrothermal vents. *ISME J.* **11**, 1545–1558 (2017). doi:10.1038/ismej.2017.37
6. Akerman, N., Butterfield, D. & Huber, J. Phylogenetic diversity and functional gene patterns of sulfur-oxidizing subseafloor Epsilonproteobacteria in diffuse hydrothermal vent fluids. *Front. Microbiol.* **4**:185 (2013).
7. Rogge, A., Vogts, A., Voss, M. & Labrenz, M. Success of chemolithoautotrophic SUP05 and Sulfurimonas GD17 cells in pelagic Baltic Sea redox zones is facilitated by their lifestyles as K- and r-strategists. *Environ Microbiol.* **19**(6), 2495–2506 (2017).
8. Haalboom, S., Price, D. M., Mienis, F., Bleijswijk, J. D. L. Van & Stigter, H. C. De. Patterns of (trace) metals and microorganisms in the Rainbow hydrothermal vent plume at the Mid-Atlantic Ridge. *Biogeosciences* **17**, 2499–2519 (2020).
9. Perner, M. *et al.* In situ chemistry and microbial community compositions in five deep-sea hydrothermal fluid samples from Irina II in the Logatchev field. *Environ. Microbiol.* **15**, 1551–1560 (2013).
10. German, C. R. et al. Volcanically hosted venting with indications of ultramafic influence at Aurora hydrothermal field on Gakkel Ridge. *Nat. Commun.* **13**, 6517 (2022).
11. Boetius, A. The Expedition PS86 of the Research Vessel POLARSTERN to the Arctic Ocean in 2014. Reports on polar and marine research, Bremerhaven, Alfred Wegener Institute for Polar and Marine Research, 685, 133 (2015). doi:doi:10.2312/BzPM_0685_2015
12. Boetius, A. & Purser, A. The Expedition PS101 of the Research Vessel POLARSTERN to the Arctic Ocean in 2016. Reports on polar and marine research, Bremerhaven, Alfred Wegener Institute for Polar and Marine Research, 706, 230 (2017). doi:doi: 10.2312/BzPM_0706_2017

13. Han, Y. & Perner, M. The globally widespread genus *Sulfurimonas*: versatile energy metabolisms and adaptations to redox clines. *Front. Microbiol.* **6**, 989 (2015).

14. Wang, S. *et al.* Characterization of *Sulfurimonas hydrogeniphila* sp . nov., a Novel Bacterium Predominant in Deep-Sea Hydrothermal Vents and Comparative Genomic Analyses of the Genus Sulfurimonas. *Front. Microbiol.* **12**:626705 (2021).

15. Inagaki, F., Takai, K., Kobayashi, H., Nealson, K. H. & Horikoshi, K. *Sulfurimonas autotrophica* gen. nov., sp. nov., a novel sulfur-oxidizing e-proteobacterium isolated from hydrothermal sediments in the Mid-Okinawa Trough. *Int. J. Syst. Evol. Microbiol.* **53**, 1801–1805 (2003).

16. Perner, M., Petersen, J. M., Zielinski, F., Gennerich, H. & Seifert, R. Geochemical constraints on the diversity and activity of H2-oxidizing microorganisms in diffuse hydrothermal fluids from a basalt- and an ultramafic-hosted vent. *FEMS Microbiol. Ecol.* **74**, 55–71 (2010).

17. Nakagawa, S. *et al.* Distribution, phylogenetic diversity and physiological characteristics of epsilon-Proteobacteria in a deep-sea hydrothermal field. *Environ. Microbiol.* **7**(10), 1619–1632 (2005).

18. Greening, C. *et al.* Genomic and metagenomic surveys of hydrogenase distribution indicate H_2 is a widely utilised energy source for microbial growth and survival. *ISME J.* **10**, 761–777 (2015).

19. Pandelia, M., Lubitz, W. & Nitschke, W. Evolution and diversification of Group 1 [NiFe] hydrogenases. Is there a phylogenetic marker for O(2)-tolerance? *Biochim Biophys Acta.* **1817**, 1565–1575 (2012).

20. Anantharaman, K., Breier, J. A., Sheik, C. S. & Dick, G. J. Evidence for hydrogen oxidation and metabolic plasticity in widespread deep-sea sulfur-oxidizing bacteria. *PNAS* **110**, 330–335 (2013).

21. Griesbeck, C., Hauska, G. & Schütz, M. Biological Sulfide Oxidation: Sulfide-Quinone Reductase (SQR), the Primary Reaction. in *Recent Research Developments in Microbiology* (ed. Pandalai, S. G.) **4**, 179–203 (Research Signpost, Trivadrum , India., 2000).

22. Argyrou, A. & Blanchard, J. S. Flavoprotein Disulfide Reductases: Advances in Chemistry and Function. *Prog. Nucleic Acid Res. Mol. Biol.* **78**, 89–142 (2004).

23. Watson, C. *et al.* Electron transfer through arsenite oxidase: Insights into Rieske interaction with cytochrome c. *BBA - Bioenerg.* **1858**, 865–872 (2017).

24. Ellis, P. J., Conrads, T., Hille, R., Kuhn, P. & Synchrotron, S. Crystal Structure of the 100 kDa Arsenite Oxidase from Alcaligenes faecalis in Two Crystal Forms at 1.64 Å and 2.03 Å. *Structure* **9**, 125–132 (2001).

25. Park, B. *et al.* Cultivation of Autotrophic Ammonia-Oxidizing Archaea from Marine Sediments in Coculture with Sulfur-Oxidizing Bacteria. *Appl. Environ. Microbiol.* **76**, 7575–7587 (2010).
