# Supplementary material for: A hydrogenotrophic Sulfurimonas is globally abundant in deep-sea oxygen-saturated hydrothermal plumes
Source: Nat Microbiol. 2023 Mar 9;8(4):651–65. doi: 10.1038/s41564-023-01342-w (PMC10066037; doi:10.1038/s41564-023-01342-w)
Supplement: Supplementary file 2 — Reporting Summary [file 41564_2023_1342_MOESM2_ESM.pdf]

nature portfolio

Corresponding author(s): Massimiliano Molari

Last updated by author(s): Jan 10, 2023

Reporting Summary

Nature Portfolio wishes to improve the reproducibility of the work that we publish. This form provides structure for consistency and transparency in reporting. For further information on Nature Portfolio policies, see our Editorial Policies and the Editorial Policy Checklist.

Statistics

For all statistical analyses, confirm that the following items are present in the figure legend, table legend, main text, or Methods section.

n/a	Confirmed	
☐	☒	The exact sample size (*n*) for each experimental group/condition, given as a discrete number and unit of measurement
☐	☒	A statement on whether measurements were taken from distinct samples or whether the same sample was measured repeatedly
☐	☒	The statistical test(s) used AND whether they are one- or two-sided<br>*Only common tests should be described solely by name; describe more complex techniques in the Methods section.*
☒	☐	A description of all covariates tested
☐	☒	A description of any assumptions or corrections, such as tests of normality and adjustment for multiple comparisons
☒	☐	A full description of the statistical parameters including central tendency (e.g. means) or other basic estimates (e.g. regression coefficient) AND variation (e.g. standard deviation) or associated estimates of uncertainty (e.g. confidence intervals)
☒	☐	For null hypothesis testing, the test statistic (e.g. *F*, *t*, *r*) with confidence intervals, effect sizes, degrees of freedom and *P* value noted<br>*Give P values as exact values whenever suitable.*
☒	☐	For Bayesian analysis, information on the choice of priors and Markov chain Monte Carlo settings
☒	☐	For hierarchical and complex designs, identification of the appropriate level for tests and full reporting of outcomes
☒	☐	Estimates of effect sizes (e.g. Cohen's *d*, Pearson's *r*), indicating how they were calculated

Our web collection on statistics for biologists contains articles on many of the points above.

Software and code

Policy information about availability of computer code

Data collection	No softwares were used for data collection
Data analysis	R v 3.5.2; IQ-Tree v2.0; Anvi'o v6.2; iTOL v6; RAxML v8.2.4; ARB v6.0.6; bbduk v34; TRIMMOMATIC v0.35; SortMeRNA v2.0; phyloFlash v3.0 beta 1; cutadapt v1.9.1; PEAR v0.9.6; swarm v.2.2.2; decompose v2.1; MEGAHIT v1.1.2; SPAdes v3.9.0; bwa v0.7.12; SAMtools v1.5; CONCOCT v1.1.0; dRep v2.3.2; Prodigal v2.6.3; HMMER v3.2.1; DIAMOND v0.9.14; RNAmmer v1.2; bwa v0.7.12; SAMtools v1.5; CheckM v.1.2.1; ARAGORN v1.2.36; Prokka v. 1.11; kallisto v0.43.1; MUSCLE v3.8.1551; blastp v2.9.0+; Bowtie2 v2.3.2; CheckM2 v.0.1.3; BUSCO v.5.2.2; MMseq2 v13.45111; CAT v.5.2.3; bwa-mem2 v.2.2.1; HTSeq v.2.0.2; Galaxy platform (https://galaxyproject.org); https://github.com/edgraham/GhostKoalaParser; edgeR v3.24.3; Adobe Illustrator CS5; Vegan v.2.5.-6; ggplot2 3.2.1.

For manuscripts utilizing custom algorithms or software that are central to the research but not yet described in published literature, software must be made available to editors and reviewers. We strongly encourage code deposition in a community repository (e.g. GitHub). See the Nature Portfolio guidelines for submitting code & software for further information.

Data

Policy information about availability of data

All manuscripts must include a data availability statement. This statement should provide the following information, where applicable:

- Accession codes, unique identifiers, or web links for publicly available datasets
- A description of any restrictions on data availability
- For clinical datasets or third party data, please ensure that the statement adheres to our policy

The sequences generated in this study and the USulfurimonas pluma genomes have been deposited in the European Nucleotide Archive (ENA) at EMBL-EBI under Bioproject PRJEB48226. All the sequences were archived using the data brokerage service of the German Federation for Biological Data (GFBio).

Sulfurimonas V3-V4 16S rRNA gene sequences were extracted from metabarcoding studies obtained from ENA (https://www.ebi.ac.uk/ena/browser/home), and the studies accession numbers are reported in Supplementary Table 5. Sulfurimonas and Sulfuricurvum kujiense full-length 16S rRNA gene sequences used in this study were obtained from SILVA RefNR database (version 138; https://www.arb-silva.de) and NCBI Genebank (https://www.ncbi.nlm.nih.gov/nucleotide; JN874148.1 and JN874176.1). Partial Sulfurimonas 16S rRNA gene sequences (250–1400 bp) were retrieved from NCBI GenBank (https://www.ncbi.nlm.nih.gov/nucleotide; data accessed May 2020), and from metabarcoding studies obtained from ENA (https://www.ebi.ac.uk/ena/browser/home) and VAMPS (vamps.mbl.edu). Sequences and studies accession numbers are reported in Fig.1. Functional genes sequences used for phylogenetic tree analysis were obtained either from UniProt (https://www.uniprot.org/uniprotkb?query=*; data accessed May 2020) or NCBI GenBank (https://www.ncbi.nlm.nih.gov/nucleotide; data accessed May 2020), and their accessions are provided in the Figure 4 and Extended Data Figure 6. Sulfurimonas and Sulfuricurvum genomes used in this study are available via NCBI GenBank (https://www.ncbi.nlm.nih.gov/nucleotide; data accessed January 2020) and accessions are reported in Supplementary Table 6a. Metagenomic and metranscitptomic data used in metapangenomic analysis were obtained either from ENA (https://www.ebi.ac.uk/ena/browser/home; data accessed May 2020) or MG-RAST (https://www.mg-rast.org; data accessed May 2020), and study accession number is provided in Supplementary Table 6b.

Field-specific reporting

Please select the one below that is the best fit for your research. If you are not sure, read the appropriate sections before making your selection.

☐ Life sciences ☐ Behavioural & social sciences ☒ Ecological, evolutionary & environmental sciences

For a reference copy of the document with all sections, see nature.com/documents/nr-reporting-summary-flat.pdf

Ecological, evolutionary & environmental sciences study design

All studies must disclose on these points even when the disclosure is negative.

Study description	Using omics' approaches, we compared the diversity and distribution of Sulfurimonas from Gakkel Ridge hydrothermal plumes with those from different Mid Ocean Ridges and other Sulfurimonas-hosting environments.
Research sample	Microbes studied in this study come form seawater samples collected in the valley of the South-West Indian Ridge (SWIR segment 10°–17°E; Atlantic sector), and at the Aurora and Polaris vent sites of the Gakkel Ridge (Central Arctic Ocean) during RV Polarstern expeditions PS81 (9th November – 16th December 2013), PS86 (7th July – 3rd August 2014), and PS101 (9th September – 23rd October 2016). Existing 16S rRNA and proteins sequences and genomes were retrieved from ENA, PubMed, SILVA SSU r138 RefNR, GeneBank, vamps.mbl.edu, UniProt, NCBI, MG-RAST.
Sampling strategy	Seawater samples were collected in the hydrothermal plume and in the surrounding waters: above the plume, below the plume, bottom water and background water (i.e. seawater without physico-chemical signatures for hydrothermal plume). Seawater samples were also collected at reference stations (i.e. not affected by hydrothermal plume) located 2 km (inside the ridge; "Internal Reference") and 56 km (outside the ridge; "External Reference") away from Aurora field, and 16 km (inside the ridge; "Internal Reference") and 190 km (outside the ridge; "External Reference") away from Polaris field.
Data collection	DNA and RNA were extracted in MPI laboratory using Powerwater DNA isolaiton Kit (Mo Bio) and mirVAna mRNA Isolation Kit (Ambion), respectively. RNA extracts were treated with the TURBO DNA-free Kit (Ambion) to remove co-extracted DNA, and purified and concentrated using RNeasy MinElute Kit (Qiagen), and only RNA with integrity number > 7 was used for sequencing. The hypervariable V3–V4 region of the bacterial 16S rRNA gene was amplified using bacterial primers S-D-Bact-0341-b-S-17 and S-D-Bact-0785-a-A-21. Sequences were obtained on the Illumina MiSeq platform in a 2 × 300 bp paired-end run with a number of reads per sample >50 000 (CeBiTec Bielefeld, Germany), following the standard instructions of the 16S Metagenomic Sequencing Library Preparation protocol (Illumina). For metagenomes paired-end libraries were prepared with the TruSeq DNA PCR-Free Sample Prep Kit (Illumina) and sequencing of libraries was performed on a MiSeq 2500 instrument (Illumina; 2 × 300 paired reads) using the v3 sequencing chemistry (CeBiTec laboratory). For metatranscriptomes the libraries were sequenced on a HiSeq1500 platform (Illumina), in a 1×150 bases single-end run and with a total number of reads per sample > 20 million.
Timing and spatial scale	Deep-sea seawater samples were collected in the summer and/or early autumn in the Arctic and Antarctic regions, as this is the time of year when there is the lowest sea ice coverage and better marine weather conditions, allowing for ship navigation and collection of deep water samples. Samples were collected based on the presence/absence of hydrothermal plume chemical-physical signal (at scale of tens of hundreds of metres), and at reference stations (from kilometres to hundreds of kilometres).
Data exclusions	No data were excluded from the analyses
Reproducibility	"Methods" section contains all information to reproduce this work, including: location of sampling sites and devices, type and number of seawater samples, protocols for sample conservation, DNA and RNA extractions and sequencing, detailed description of workflow applied for data analysis and all the softwares, and references and/or accession numbers for sequences downloaded from public databases.
Randomization	In this comparative study of Arctic Ocean and global deep-sea microbiota, we identified a new aerobic Sulfurimonas species inhabiting neutrally buoyant hydrothermal plumes and fluids, and subsurface aquifers. Therefore randomization is not applicable to this study.
Blinding	It is not applicable here, as this study does not include clinical trials.

Did the study involve field work? ☒ Yes ☐ No

Field work, collection and transport

Field conditions	Seawater samples were collected at water depth > 2000 m, and the parameters mostly affecting the samples were those related to presence of hydrothermal plumes (i.e. anomalies in temperature, turbidity and redox). Sampling activities were mostly affected by marine weather conditions at South Atlantic Ocean, and by sea-ice drift in the Arctic Central Ocean.
Location	Seawater samples were collected at South-West Indian ridge (LAT. from -52.646 to -52.233, LONG. from 12.526 to 15.738; water depth 2500-4320 m) and at the Gakkel Ridge in Aurora (LAT. from 82.896 to 83.105, LONG. -6.377 to -2.466; water depth 2000-3955 m) and Polaris (LAT. from 85.292 to 86.984, LONG. from 55.575 to 60.184; water depth 2051-4841 m) hydrothermal fields.
Access & import/export	All samples obtained are from the High Seas, except the samples from Auora from Greenland EEZ for wich diplomatic permission was obtained. No further permission was needed to collect and use the samples. The research vessel complies with all environmental standards in polar seas, and the participants sign a compliance clause for sustainable sampling and data management. There were no specific import clauses for the water samples obtained.
Disturbance	Sampling activities included exclusively in situ physiochemical measurements by sensors, collection of seawater (tens of litres) and in situ seawater filtration (hundreds of litres), therefore causing only limited disturbance to the environment and organisms.

Reporting for specific materials, systems and methods

We require information from authors about some types of materials, experimental systems and methods used in many studies. Here, indicate whether each material, system or method listed is relevant to your study. If you are not sure if a list item applies to your research, read the appropriate section before selecting a response.

Materials & experimental systems

n/a	Involved in the study
☒	☐ Antibodies
☒	☐ Eukaryotic cell lines
☒	☐ Palaeontology and archaeology
☒	☐ Animals and other organisms
☒	☐ Human research participants
☒	☐ Clinical data
☒	☐ Dual use research of concern

Methods

n/a	Involved in the study
☒	☐ ChIP-seq
☒	☐ Flow cytometry
☒	☐ MRI-based neuroimaging
